# Development and Evaluation of a Novel Set of EST-SSR Markers Based on Transcriptome Sequences of Black Locust (*Robinia pseudoacacia* L.)

**DOI:** 10.3390/genes8070177

**Published:** 2017-07-07

**Authors:** Qi Guo, Jin-Xing Wang, Li-Zhuo Su, Wei Lv, Yu-Han Sun, Yun Li

**Affiliations:** 1 Beijing Advanced Innovation Center for Tree Breeding by Molecular Design, National Engineering Laboratory for Tree Breeding, College of Biological Sciences and Technology, Beijing Forestry University, Beijing 100083, China; Guoqi0529@126.com (Q.G.); Xing521-zi@126.com (J.-X.W.); 18037935780@163.com (W.L.); sunyuhan@bjfu.edu.cn (Y.-H.S.); 2 State-Owned Linghai Hongqi Forest, Jinzhou 121228, China; scarlett-2@163.com

**Keywords:** *Robinia pseudoacacia*, EST-SSR markers, polymorphism, Illumina sequencing

## Abstract

Black locust (*Robinia pseudoacacia* L. of the family Fabaceae) is an ecologically and economically important deciduous tree. However, few genomic resources are available for this forest species, and few effective expressed sequence tag-derived simple sequence repeat (EST-SSR) markers have been developed to date. In this study, paired-end sequencing was used to sequence transcriptomes of *R. pseudoacacia* by the Illumina HiSeq TM2000 platform, and EST-SSR loci were identified by de novo assembly. Furthermore, a total of 1697 primer pairs were successfully designed, from which 286 primers met the selection screening criteria; 94 pairs were randomly selected and tested for validation using polymerase chain reaction amplification. Forty-five primers were verified as polymorphic, with clear bands. The polymorphism information content values were 0.033–0.765, the number of alleles per locus ranged from 2 to 10, and the observed and expected heterozygosities were 0.000–0.931 and 0.035–0.810, respectively, indicating a high level of informativeness. Subsequently, 45 polymorphic EST-SSR loci were tested for amplification efficiency, using the verified primers, in an additional nine species of Leguminosae, 23 loci were amplified in more than three species, of which two loci were amplified successfully in all species. These EST-SSR markers provide a valuable tool for investigating the genetic diversity and population structure of *R. pseudoacacia*, constructing a DNA fingerprint database, performing quantitative trait locus mapping, and preserving genetic information.

## 1. Introduction

Black locust (*Robinia pseudoacacia* L.), a model species of *Robinia*, is a deciduous forest tree that has significant economic and ecologic value in China, due to its rapid growth, excellent drought resistance, and good adaptability to the local environment [1,2]. It originated in the southeast region of North America and was first introduced to Nanjing in 1877–1878. The black locust was planted widely in China at the end of the 19th century, and it developed successfully as an exotic species [3,4]. Currently, *R. pseudoacacia* is distributed widely in China, particularly in regions north of the Yangtze River, and plays an essential role in afforestation and environmental improvement. Therefore, successful management and development of this resource requires understanding of the genetic diversity and population structure of natural populations, to maximize the selection, conservation, and utilization of elite germplasms [5]. However, the limited number of efficient molecular markers, particularly codominant markers such as simple sequence repeats (SSRs), greatly obstructs its use in breeding studies.

Microsatellite markers (or SSRs) are prevalent molecular markers used for studying the population genetics of plants and animals [6,7,8]. SSR markers are superior to other traditional DNA-based molecular markers (i.e., restriction fragment length polymorphism (RFLP), random amplification of polymorphic DNA (RAPD), amplified fragment length polymorphism (AFLP), sequence-related amplified polymorphism (SRAP), and inter simple sequence repeats (ISSR)) [9,10,11,12,13,14] as they display codominance, high information content, and locus specificity. With the development of high-throughput sequencing technologies, a large number of single nucleotide polymorphism (SNP) markers have been developed in recent years [15]. However, SSR markers remain a powerful tool in studying genetic diversity, determining population structure, constructing DNA fingerprint databases, generating genetic maps, and predicting molecular marker-assisted breeding, due to their good reproducibility and cost-effectiveness [16]. SSR markers applied to black locust are rare; Lian and Hogetsu [17] isolated seven polymorphic microsatellite loci using a dual-suppression-PCR technique, and Mishima et al. [18] isolated 11 microsatellite loci using an enrichment method [19] with some modifications. Such limited SSR marker development was based on genomic DNA levels and not expressed sequence tags (ESTs). Genomic DNA-derived SSRs (G-SSRs), and EST-derived SSRs (EST-SSRs), are two types of SSR markers. Abundant EST sequences and molecular markers can be generated by transcriptome sequencing, which involves constructing a complementary DNA (cDNA) library, followed by sequencing using the Illumina HiSeq sequencing platform [20,21]. EST-SSRs have an advantage over G-SSR markers in that EST-SSRs are derived from the coding regions of genomes [22,23,24,25,26]; therefore, they demonstrate significant amplification efficiency and reveal conserved sequences among related species [27,28]. Although EST-SSRs are widely developed and have been applied to numerous species, reports on EST-SSR markers for black locust using transcriptome data are scarce, greatly limiting research on genetic variation, germplasm preservation, and molecular breeding in this species.

In the present study, we developed EST-SSRs from the transcriptome sequencing of *R. pseudoacacia* by carefully selecting 45 EST-SSRs primer pairs with high-resolution and high polymorphism, to assess the genetic diversity and population structure. Moreover, this technique facilitated the construction of a DNA fingerprint database of black locust, providing tools for understanding the genetic variation, developing appropriate germplasm conservation strategies, and laying the foundation for future molecular genetic research.

## 2. Materials and Methods

### 2.1. Unigene Acquisition

The unigenes used to develop SSR makers in this study came from the transcriptome sequencing data acquired by Wang et al. [29] (accession number: PRJNA260115), and are listed in Supplementary Materials S1.

### 2.2. EST-SSR Detection and Primer Design

Potential microsatellite repeats were detected from 36,533 unigenes using the MIcroSAtellite perl script [29]. The SSR motifs were searched for mono-, di-, tri-, tetra-, penta- and hexa-nucleotides, with a minimum number of repeat units of 12, 6, 5, 5, 4, and 4, respectively. Mono-nucleotide repeats were removed from the analysis, because the sequencing itself generates stretch errors [30]. An online software, BatchPrimer3, was used to design SSR-specific primers [31]. The major parameters for primer pair design were as follows: a minimum number of SSR pattern repeats of 10 for di-nucleotides, seven for tri-nucleotides, four for tetra-nucleotides, four for penta-nucleotides and three for hexa-nucleotides; minimum and maximum product sizes of 100–500 bp (optimal: 150 bp); primer length of 18–25 bases (optimal: 21 bases); GC content of 30–70% (optimal: 50%); annealing temperatures of 50–60 °C (optimal: 56 °C); and default values for the other parameters.

### 2.3. EST-SSR Identification and Validation

For polymorphism analyses of the EST-SSRs, 32 individuals of *R. pseudoacacia* (Table S1) were collected from four artificial planting distribution regions in China: Luoning county, Luoyang, Henan (34°23′44″ N, 111°39′7″ E); Ji County, Linfen, Shangxi (36°6′16″ N, 111°41′21″ E); Fei County, Linyi, Shangdong Province (35°16′17″ N, 117°58′47″ E); and Pingquan, Chengde, Hebei, (41°1′25″ N, 118°42′33″ E). Genomic DNA was isolated from the 32 dried samples using a plant genomic DNA kit (Tiangen, Beijing, China). DNA quality and concentration were verified via 1% agarose gel electrophoresis. The concentration and purity of the total DNA were further quantified using the NanoDrop 2000 (Thermo Fisher Scientific, Wilmington, DE, USA). The total DNA was diluted and aliquoted at equal concentrations (20 ng/μL) with elution buffer TB (Tiangen), and stored immediately at −80 °C for PCR amplification.

PCR technology was used to validate the quantity of synthetic primer pairs in a volume of 20 μL, which contained 2 μL genomic DNA (20 ng/μL), 10 μL of 2x TSINGKE^®^ Master Mix (blue) (Beijing TsingKe Biotech Co., Ltd., Beijing, China), 4 μL of M13 primer (1 μM; 5′-TGTAAAACGACGGCCAGT-3′) labeled at the 5′ end of the forward primer with fluorescent-dye (FAM, HEX, ROX and TAMRA), using a technique that could easily identify the four fluorescent labels [32], 0.8 μL of the forward primer (1 μM), and 3.2 μL of the reverse primer (1 μM). PCR conditions were as follows: denaturation at 94 °C for 4 min followed by 28 cycles at 94 °C for 30 s, 55–59 °C for 30 s (optimal annealing temperatures are given in Table 1), and 72 °C for 1 min, followed by 10 cycles at 94 °C for 30 s, 50 °C for 30 s, 72 °C for 45 s, and a final extension at 72 °C for 10 min using a BIO-RAD T100 thermal cycler [32,33]. PCR products were subjected to analysis using an ABI 3730XL DNA Sequencer (Applied Biosystems, Foster City, CA, USA). The alleles of SSRs were confirmed using the GeneMarker version 2.2.0 software package (SoftGenetics LLC, State College, PA, USA).

POPGENE version 1.32 software [34] was used to evaluate population genetic parameters including the observed number of alleles (Na), effective number of alleles (Ne), Shannon’s Information index (I), observed heterozygosity (Ho), expected heterozygosity (He), Polymorphism information content (PIC) and Hardy–Weinberg equilibrium (HWE) [34]. PIC was calculated using PIC-CALC version 0.6 [35] and the null allele frequency (Null freq.) using Cervus 3.0 software [36].

### 2.4. EST-SSR Amplification in Related Species

EST-SSR markers are often quite well-conserved among congeneric species. Six genera of Leguminosae from Beijing Forestry University Campus, China (40°0′22″ N, 116°21′1″ E) were used to evaluate the potential value of the developed set of 45 EST-SSR markers in other related species, including *Gleditsia* (Caesalpinioideae), *Cercis* (Caesalpinioideae), *Wisteria* (Papilionoideae), *Trifolium* (Papilionoideae), *Amorpha* (Papilionoideae), *Sophora* (Papilionoideae), and *Robinia* (Papilionoideae). A total of nine species were selected in the genera *Sophora*, including *Sophora japonica* and *Sophora japonica* var. *pendula*, and *Robinia*, including *R. pseudoacacia* ‘Frisia’ and *R. pseudoacacia* var. *decaisneana*. The genomic DNA extraction, PCR amplification, and fluorescence modification, were performed as described above, except that the annealing temperature was re-optimized for each locus.

## 3. Results and Discussion

A total of 5072 potential EST-SSR loci were identified from 36,533 unigenes, the tri-nucleotides were the most abundant type of repeat (2321, 45.761%), followed by di-nucleotide repeats (1889, 37.244%), tetra-nucleotide repeats (270, 5.323%), and the penta- and hexa-nucleotide repeats at approximately equal frequencies (Table 2). In total, 2486 primer pairs were successfully designed from 4781 potential EST-SSR loci, of which 789 primers targeted the same unigenes, and we obtained 1697 SSR primer pairs targeting unique unigenes. These primers met a series of rigorous screening criteria described in the previous section. Subsequently, 286 primers were successfully selected, of which 94 pairs were randomly selected and synthesized by Sangon Biotech (Shanghai, China) for validation. Ultimately, these 94 primer pairs were used to evaluate whether the potential EST-SSRs were polymorphic and informative for use in germplasm conservation and population genetics. This set of 94 primer pairs targeting EST-SSRs was further filtered by analyzing a group of germplasms containing 32 black locust individuals collected from four different regions in China.

Of the 94 primer pairs, 45 (47.9%) amplified a pure fragment of the expected size, whereas 11 (11.7%) yielded a weak amplification, 16 (17.0%) failed to amplify any product, and 22 (23.4%) amplified nonspecific products. Of the 45 loci amplified, 3, 10, 8, 5, and 19 contained di-, tri-, tetra-, penta-, and hexa-nucleotide repeats, respectively. The polymorphism frequencies of these di-, tri-, tetra-, penta-, and hexa-nucleotide repeat markers were 6.7%, 22.2%, 17.8%, 11.1%, and 42.2%, respectively.

Detailed characteristics, primer sequences, and genetic information regarding the novel set of 45 polymorphic EST-SSR markers are presented in Table 1. The sequences of the affected unigenes are presented in Supplementary Materials S1. A total of 194 alleles were detected in 32 individuals. The Na per locus ranged from 2 to 10, with an averaging 4.267 alleles per locus. The Ne was 1.035–4.845, averaging 2.123 over all loci. The Ho ranged from 0.000 at locus Rp-08 to 0.931 at locus Rp-12, averaging 0.379 over all loci. The He ranged from 0.035 at loci Rp-25 and Rp-27 to 0.810 at locus Rp-04, averaging 0.447 over all loci. I was 0.087–1.802, with a mean of 0.836 per marker. The PIC ranged from 0.033 at loci Rp-16, Rp-25 and Rp-27 to 0.765 at locus Rp-04, with a mean of 0.379 per marker. The PIC of the Rp-15 primer pairs exceeded 0.500, showing a high level of informativeness. HWE and the Null freq. at each locus showed significant differences. The Ho and He values were quite different from each other at many loci, indicating a significant deviation from HWE. At the same time, due to the presence of ineffective alleles, it also caused the deficiency or excess of observed heterozygotes. Here, there are three cases that explain the null allele frequency: (1) When the microsatellite flanking sequence is mutated, one (or both) primer(s) do not bind to their target site at a particular allele, resulting in the locus failing to amplify; (2) When the difference in the size of the alleles is greater than 150 bp, the advantage of amplifying the smaller allele is significantly larger than that of the large fragment—a few samples show only the small fragment allele, resulting in site band deletion or an excess of homozygous individuals; (3) If one of the two alleles in the heterozygous state of a given locus is a null allele, only a single band appears after PCR, and the locus is therefore mistakenly interpreted as being homozygous. Therefore, a high incidence of null alleles always causes an excess of homozygotes (i.e., heterozygote deficiency).

SSR markers of *R. pseudoacacia* have been developed and used in other related species [17,18]. The Na ranged from 4 to 12, with an average of 8.2; Ho and He ranged from 0.333 to 0.821 and 0.489 to 0.867, respectively (based on 11 SSR markers from 39 individuals) [18]. In the red clover (*Trifolium pretense* L.), the Na ranged from 2 to 25, and the average Ho and He values were 0.71 and 0.88, respectively (based on 27 SSR markers from 24 individuals) [35]. In *Pisum sativum* (Leguminosae), the Na ranged from 1 to 7, and the Ho and He ranged from 0 to 0.889 and 0 to 0.840, respectively (based on 41 SSR markers from 32 individuals) [36].

The results of amplification of the EST-SSRs in related species are summarized in Table 3. The amplification efficiency varied from 0 to 100% in nine Leguminosae species. Compared with other primers, the primer pair targeting locus Rp-24 showed the lowest (2.2%) amplification efficiency, and the other 44 primer pairs produced a moderate level of amplification. There were 23 (51.1%) loci (Rp-01, Rp-04, Rp-05, Rp-07, Rp-10, Rp-11, Rp-15, Rp-16, Rp-17, Rp-19, Rp-23, Rp-25, Rp-26, Rp-27, Rp-28, Rp-29, Rp-31, Rp-32, Rp-33, Rp-37, Rp-38, Rp-39, Rp-40, and Rp-43) that could be amplified in more than three species in the family Leguminosae, of which two loci (Rp-01 and Rp-29) were amplified successfully in all nine species tested. These results suggest that the novel set of EST-SSR markers developed from transcriptomic sequences of *R. pseudoacacia* in this study could be useful, not only for genetic studies of *R. pseudoacacia* and other related species, but also for identification of this economically and ecologically important plant species.

In summary, these 45 primer pairs targeting SSRs identified abundant polymorphisms that can be used to evaluate the genetic diversity and population structure of this species, and to provide a practical strategy for selecting elite germplasms for conservation and utilization. Furthermore, we report the development, synthesis, and verification of SSR markers using Illumina paired-end sequencing of *R. pseudoacacia*. Using this set of EST-SSR markers, additional research can be implemented to investigate the relationships of inter- and intra-species construct genetic linkage maps and association maps of *R. pseudoacacia*.

## 4. Conclusions

A novel set of EST-SSR markers in black locust was successfully developed and characterized via transcriptome sequencing. The 45 SSR primer pairs displayed abundant polymorphisms that can be used to evaluate the genetic diversity and population structure of this species, construct a DNA fingerprint database, and provide a practical strategy for selecting elite germplasms for conservation and utilization.

## Figures and Tables

**Table 1:** Characteristics of the 45 polymorphic expressed sequenced tags-derived simple sequence repeats (EST-SSR) markers in 32 individuals from *Robinia pseudoacacia*.

Locus	Primer Sequence (5′-3′)	Repeat Motif	Size (bp)	Ta (°C)	Na	Ne	I	Ho	He	PIC	HWE	Null Freq
Rp-01	F: TGCAGAAAGAGAAAGCAGAGG	(TGTGAA)_4_	140	58	6	2.713	1.195	0.724	0.643	0.573	ns	−0.0953
	R: CCGAACCCTTTCTGGTTAGTC											
Rp-02	F: GCTGCGTTTAATTTTGTCAGG	(GAAT)_4_	170	58	6	1.193	0.376	0.138	0.165	0.156	***	+0.1532
	R: TCAATCCATCAAAGAGGAAACA											
Rp-03	F: GTGAGAAGTGGTTAGGGTTTT	(CTC)_7_	186	55	4	2.740	1.094	0.633	0.646	0.562	ns	+0.0057
	R: TCAAGATCACCAACGTACAA											
Rp-04	F: CTCGTGATGATGGTGTTGATG	(AATGGT)_4_	146	58	10	4.845	1.802	0.480	0.810	0.765	***	+0.2622
	R: AATGGTCCAAACAACACGAAG											
Rp-05	F: CCTTGCACATTTATCCCAGAA	(TCTGGC)_3_	158	57	6	3.254	1.364	0.333	0.707	0.641	ns	+0.3662
	R: CGACCTCGATCTTTTCTTGTG											
Rp-06	F: TGGACAAAACATCATCGTGTG	(TGAGTT)_4_	147	59	5	3.492	1.370	0.536	0.723	0.669	***	+0.1492
	R: CTCTCTTCTTTCTGCCCCTCA											
Rp-07	F: TTTTTCTCCCAACGAAACAAA	(CT)_10_	144	56	5	3.565	1.436	0.296	0.733	0.682	***	+0.4159
	R: TGATGTGTTGTACGGAGGTGA											
Rp-08	F: TCAGGTGCATAAGCTCATTACTTC	(AAAAT)_4_	152	56	3	2.074	0.810	0.000	0.528	0.418	***	+0.9969
	R: GGTTGTCAGATGAAATGCACA											
Rp-09	F: CGTTTAGAAGCTGAGGCAGAA	(CTTT)_5_	153	58	6	3.174	1.377	0.793	0.697	0.632	***	−0.1080
	R: TGAGATATCTTAGTGCAGGAGCA											
Rp-10	F: GGCATGTGGCTATGAAGATGT	(CCTTT)_4_	154	57	2	1.212	0.318	0.194	0.178	0.160	ns	−0.0441
	R: TCAGTGGGACTTGGTTTCTTG											
Rp-11	F: GAAGCTATCACCGCAAATGAA	(AG)_10_	150	57	8	3.867	1.675	0.593	0.755	0.715	***	+0.1241
	R: GTCGAAGTGCGTCCTAGATCA											
Rp-12	F: AAGAGTCATCACGGAGACCAA	(AGCAGA)_4_	150	56	4	3.885	1.371	0.931	0.756	0.695	***	−0.1117
	R: GGAGTCCAATTAAGTGCGAGA											
Rp-13	F: CATTTCCGATTTCCAATTCCT	(CTCTTC)_4_	151	56	4	1.602	0.755	0.444	0.383	0.352	ns	−0.1136
	R: GCCGAGGACTCGGTAGAAGT											
Rp-14	F: TTAGCACGAACCTGGTTATGG	(TGCAAC)_4_	151	56	3	1.198	0.346	0.107	0.168	0.156	*	+0.2087
	R: CACTTCATTTGGTTCCTTGAGA											
Rp-15	F: TTAACTAATGCGGCGAGAAGA	(TCAC)_5_	119	56	9	1.560	0.909	0.313	0.365	0.351	***	+0.1258
	R: GAGAGGAAGTGTGCGAAACAA											
Rp-16	F: TATGAGACAGTGTTGGTTGGT	(TTCAGT)_4_	175	56	2	1.035	0.087	0.313	0.365	0.033	ns	−0.0026
	R: CGTGCCAGAAGAGTATAACAG											
Rp-17	F: GTAAGTCTGCAAAGAAGACCA	(AACCA)_4_	150	56	3	2.078	0.890	0.129	0.527	0.463	***	+0.5965
	R: GCTTTTCACCTATCAACTCAA											
Rp-18	F: GGATGAACTTTGGCAATCCTT	(GGTCAG)_4_	158	55	6	3.016	1.310	0.792	0.683	0.611	***	−0.0916
	R: AATTTGTTGGGAATGCTGTTG											
Rp-19	F: CAGGAGTGGCAGCATTAGTGT	(AGGCTG)_4_	123	56	4	2.237	0.948	0.357	0.563	0.473	ns	+0.2144
	R: CACAACAAGCACATTTTGCAC											
Rp-20	F: TTTCTTGGCTTGCTTTTGCTA	(GCAGCT)_3_	145	56	3	1.070	0.169	0.033	0.066	0.064	***	+0.4243
	R: TCTTGGATACGCAAGGTTGTC											
Rp-21	F: TATGATCACGTCCCCTAATGC	(CCA)_7_	146	57	10	1.817	1.119	0.438	0.457	0.438	***	−0.0054
	R: AAGTGGAAAGAAATGGGATGG											
Rp-22	F: GGTAAGGTGAAGGAGGTGGAG	(AGGGTT)_4_	150	56	4	2.003	0.961	0.571	0.510	0.464	ns	−0.0674
	R: AGCTTGGTCTCCTAGGTCGTC											
Rp-23	F: GGAGGAGCAACCATCTGTGTA	(AGAAGT)_4_	146	56	2	1.960	0.683	0.714	0.499	0.370	*	−0.1864
	R: CTCCCTCTTCATCCTCACCTC											
Rp-24	F: TGCACATATTTGCCTGGTTTA	(AATA)_4_	160	56	2	1.039	0.095	0.039	0.039	0.037	ns	−0.0031
	R: AAAATGAGCATGACACAACCA											
Rp-25	F: CGGCAACAAGTTGAGAAGAAC	(AAAG)_5_	139	56	2	1.035	0.087	0.035	0.035	0.033	ns	−0.0026
	R: GGCTCACAAACCAACCTATGA											
Rp-26	F: GCTGCAAGCAAAGGATCTTAC	(ATGATA)_4_	139	56	3	1.286	0.428	0.250	0.227	0.205	ns	−0.0607
	R: CCTCATCATCCTCGTCATCAT											
Rp-27	F: TGGACAAAACATCATCGTGTG	(TGAGTT)_4_	147	57	2	1.035	0.087	0.035	0.035	0.033	ns	−0.0026
	R: CTCTCTTCTTTCTGCCCCTCA											
Rp-28	F: CTTGGTCTAGAAAGTCCTGCT	(CAG)_7_	151	56	5	2.288	1.101	0.552	0.573	0.522	ns	+0.0274
	R: GGTCATCAAGGTTAGTTGGAT											
Rp-29	F: CCTGATGATCAAAACGACGAC	(GATC)_4_	148	56	4	1.180	0.365	0.033	0.155	0.148	***	+0.6003
	R: GGAGGTGACCCCTCTTATCCT											
Rp-30	F: TTGAACCAAAACTGGAAGAGC	(GCT)_8_	151	56	5	1.977	0.950	0.103	0.503	0.445	***	+0.6526
	R: GCACCGTACAGTTACCCTATCC											
Rp-31	F: GACCCCATTTTTCTCAAGGAC	(ATT)_7_	140	56	3	1.362	0.487	0.270	0.271	0.239	ns	+0.0347
	R: TTGGATAAGTCGGTGAAGGTG											
Rp-32	F: CCACGTGGTTCTTCAAACATT	(GTG)_7_	163	57	4	2.176	0.900	0.625	0.549	0.449	ns	−0.0649
	R: CAACAACAACCCACAAACACA											
Rp-33	F: CAAACAGTCTCATGGAAATGGA	(ATC)_7_	141	56	2	1.533	0.532	0.448	0.354	0.287	ns	−0.1253
	R: GGGTTGGTATTGTTGGGAAAT											
Rp-34	F: AGGATATTAGCCAAGTCCATC	(TGGTGA)_4_	164	57	3	1.640	0.701	0.259	0.398	0.352	**	+0.1915
	R: AGTAACCATCACCACAATCAC											
Rp-35	F: TCAGACGTGGTAGAGCAGTGTT	(CACAC)_4_	152	58	3	2.718	1.049	0.286	0.644	0.560	***	+0.3910
	R: ATTTGTTTTTGGGGGAGATTG											
Rp-36	F: CGTTTCAGCCATTGATTTTGT	(GAATC)_5_	141	57	4	2.475	1.049	0.519	0.607	0.533	ns	+0.0857
	R: GATCATCACCGTCCACCTTC											
Rp-37	F: TGTCGTCATTTTATTTTACCC	(GAACGA)_4_	152	56	6	2.230	1.157	0.643	0.562	0.522	***	−0.1265
	R: CTCACCCTTTTTATTTCCATT											
Rp-38	F: TCCATTCCCTGGTTTCTTCTT	(TC)_10_	150	56	10	3.896	1.641	0.767	0.756	0.704	***	−0.0357
	R: AGCACAATTTCCTCAGTGCAG											
Rp-39	F: TTAAAGAATGTTCCGTTCAGA	(AAGAGG)_3_	152	56	4	2.198	0.901	0.462	0.556	0.449	***	+0.0679
	R: GAGAAGATAGCCTCCTAGCTG											
Rp-40	F: TCATTGGACATCCCTCCATAA	(TAA)_8_	139	56	2	1.427	0.476	0.300	0.305	0.255	ns	−0.0009
	R: GGCTCGACATGGTTGATTTT											
Rp-41	F: AACTCACCCAATTGCACACTC	(CCA)_7_	143	56	4	1.985	0.872	0.654	0.506	0.431	**	−0.1463
	R: GAGCAAGAGCTAAAGCAGCAA											
Rp-42	F: CTTCGCAATCCTCACTCTTTG	(AATC)_4_	169	55	2	1.882	0.662	0.250	0.477	0.359	*	+0.3043
	R: CTTACCCAGAAGCCAACAATG											
Rp-43	F: CAAAGCAGAGAGAATGTATGG	(CAAAAT)_4_	155	57	4	1.909	0.879	0.548	0.484	0.434	ns	−0.0678
	R: ATCCCTTGCTCCTTGTAATAG											
Rp-44	F: TATCTGGGAGAATCGAGAGCA	(ATCA)_5_	145	57	2	1.127	0.227	0.120	0.115	0.106	ns	−0.0219
	R: CCACCATGGTTGTCCTTCTAA											
Rp-45	F: GGGTTGAGGAAGAGAGGAGAA	(TTC)_7_	156	57	3	1.561	0.630	0.296	0.366	0.316	***	+0.0830
	R: AAAAATCGAATCGTGTTGGTG											
Mean					4.267	2.123	0.836	0.379	0.447	0.397		0.1110
												

Na, Observed number of alleles; Ne, Effective number of alleles; I, Shannon’s Information index; Ho, Observed heterozygosity; He, Expected heterozygosity; PIC, Polymorphism information content; HWE: Deviation from Hardy–Weinberg equilibrium, ns = not significant; * *p* < 0.05; ** *p* < 0.01; *** *p* < 0.001.

**Table 2:** Length distribution of the EST-SSRs based on the number of *R. pseudoacacia*nucleotide repeat units.

Number of Repeats	Mono-	Di-	Tri-	Tetra-	Penta-	Hexa-	Total	Percentage (%)
4					133	149	282	5.560
5			1351	158	19		1528	30.126
6		603	624	112			1339	26.400
7		375	296				671	13.229
8		289	50				339	6.684
9		252					252	4.968
10		242					242	4.771
11		115					115	2.267
12	122	13					135	2.662
13	66						66	1.301
14	46						46	0.907
15	23						23	0.453
16	7						7	0.138
17	4						4	0.079
18	3						3	0.059
19	1						1	0.020
20	5						5	0.099
21	1						1	0.020
22	4						4	0.079
23	8						8	0.158
24	1						1	0.020
Total	291	1889	2321	270	152	149	5072	
Percentage (%)	5.737	37.244	45.761	5.323	2.997	2.938		

**Table 3:** Amplification efficiency in related species of Leguminosae using the 45 EST-SSR-targeting primers developed based on transcriptomic sequences of *R. pseudoacacia*.

Locus	*Gleditsia sinensis*	*Cercis chinensis*	*Wisteria sinensis* (Sims) Sweet	*Trifolium repens*	*Amorpha fruticosa*	*Sophora japonica*	*Sophora japonica* var. *pendula*	*Robinia pseudoacacia* ‘Frisia’	*Robinia pseu-doacacia* var. *decaisneana*	Total Species
Rp-01	+	+	+	+	+	+	+	+	+	9
Rp-02	−	−	+	−	−	+	−	−	+	3
Rp-03	−	−	−	−	−	−	−	+	+	2
Rp-04	−	−	+	−	−	+	−	+	+	4
Rp-05	−	−	+	+	+	+	+	−	+	6
Rp-06	−	−	−	+	−	−	−	+	+	3
Rp-07	+	−	+	−	+	−	−	−	+	4
Rp-08	−	−	−	−	−	−	−	+	+	2
Rp-09	−	+	−	−	−	−	−	+	+	3
Rp-10	+	+	+	+	+	−	+	+	+	8
Rp-11	−	−	+	−	+	−	+	+	+	5
Rp-12	−	−	+	+	+	−	+	+	+	6
Rp-13	−	−	−	−	−	−	+	+	+	3
Rp-14	−	−	−	−	−	−	−	+	+	2
Rp-15	+	+	+	+	−	+	+	+	+	8
Rp-16	−	−	+	+	+	+	−	+	+	6
Rp-17	+	+	+	+	+	+	−	+	+	8
Rp-18	−	−	−	−	−	−	−	+	+	2
Rp-19	−	+	+	+	+	+	+	+	+	8
Rp-20	−	−	−	−	−	−	−	+	+	2
Rp-21	−	−	−	−	−	−	+	+	+	3
Rp-22	−	−	−	−	−	−	−	+	+	2
Rp-23	−	+	−	−	−	−	+	+	+	4
Rp-24	−	-	−	−	−	−	−	−	−	0
Rp-25	+	+	+	−	−	+	+	+	+	7
Rp-26	+	+	+	+	+	−	+	+	+	8
Rp-27	+	−	+	+	+	+	+	+	+	8
Rp-28	−	−	+	+	+	+	−	+	+	6
Rp-29	+	+	+	+	+	+	+	+	+	9
Rp-30	−	−	−	−	−	-	−	+	+	2
Rp-31	+	+	−	−	−	+	−	+	−	4
Rp-32	+	−	+	−	+	+	+	−	+	6
Rp-33	+	+	+	+	+	−	−	+	+	7
Rp-34	−	−	−	−	−	−	−	+	+	2
Rp-35	+	+	−	−	−	−	−	−	+	3
Rp-36	−	+	−	−	−	−	−	+	−	2
Rp-37	−	−	+	+	−	+	−	+	+	5
Rp-38	+	−	+	−	+	+	−	+	−	5
Rp-39	−	−	+	+	−	+	+	−	+	5
Rp-40	+	+	−	−	−	+	+	+	+	6
Rp-41	−	−	−	−	−	−	−	+	+	2
Rp-42	−	−	−	−	−	−	−	−	+	1
Rp-43	+	+	+	−	+	+	−	+	+	7
Rp-44	+	−	−	−	−	−	−	+	−	2
Rp-45	+	−	−	−	−	−	−	+	+	3
No. of loci	18	16	23	16	17	19	17	37	40	

“+” indicates successful PCR amplification, and “−” indicates failed PCR amplification.

## Supplementary Materials

The following are available online at www.mdpi.com/2073-4425/8/7/177/s1. Supplementary Materials S1: List of unigenes used to develop SSR markers in this study. Table S1: List of the experimental individuals of *Robinia pseudoacacia* L.
