# Supplementary material for: Development and Evaluation of a Novel Set of EST-SSR Markers Based on Transcriptome Sequences of Black Locust (Robinia pseudoacacia L.)
Source: Genes (Basel). 2017 Jul 7;8(7):177. doi: 10.3390/genes8070177 (PMC5541310; doi:10.3390/genes8070177)
Supplement: Supplementary file 1 [file genes-08-00177-s001.zip › Supplementary Materials S1.docx]

Accession NO. PRJNA260115

>Rp-01

ATCTGGGGACATAATAGTAATTTAACGGTGAATTCTTTCTTACCTTCAATTTTAATATCCTGCCTGCTACGGTGGAGTGCGTGTGGGCGAGTCTAGATTCTATCGATGGCGGCTGCATTT

ACATTCACAACACCAACACTGTCAAATGTTGCAGAAAGAGAAAGCAGAGGCAGAATCAGAGCATTGAACCCTAGCCTTACCCCTGCTATCGGGGTGGTTAGGTTTGGTAGGCGTGTGAATGTGAATGTGAATGTGAATGGTTGTTGTCGACTAACCAGAAAGGGTTCGGGAGTCTTCCGGGTGAGGGCCAGTGGCAGTGGCAGTGGCAGTTCGGAGGAACCGGCAGCGGCGGCTCTACAGTCGAAAGTGACTCACAAAGTATACTTTGATATCAGCATTGGGAATCCAGTTGGGAAGCTTGCAGGAAGGATTGTAATTGGACTTTACGGTGACGATGTGCCCCAAACAGCTGAGAACTTCCGTGCCCTTTGTACTGGAGAGAAGGGCTTTGGCTACAAGGGCTCCACCTTCCATCGTGTCATCAAGGATTTCATGATTCAAGGAGGCGACTTTGACAAAGGAAATGGAACTGGGGGCAAAAGTATATATGGTCGTACTTTCAAAGATGAGAACTTTAAATTGTCTCATACTGGACCAGGAGTTCTTAGCATGGCAAATGCAGGTCCCAACACAAATGGGAGTCAGTTTTTCATATGCACTGTGAAGACACCATGGCTGGATCAGAGACATGTTGTATTTGGCCAAGTTTTGGAAGGCATGGACATTGTTAGGTTGATTGAATCACAGGAGACAGATCGGGGTGACCGACCTAGAAAGAGGGTGGTTATCAGTGACTCTGGCGAGCTTCCAATTGCTTGAGGTGGTTCTTGTGTATCTTGTTGATCATGGTTTCTATTTGTTTCTTGCTGCTGAGAAGGGAAGATAAAAAAAGAAAGGAAGAATCTCCTGCTTTCTGGAAATTTTTGTTTGACTATTCTATAAGATGAGTTCAGCACCTTTTTATTTTCATACTGAACCTACAAACTTTTAGCAGCTATCTGTATTCTATAACAGAATTGAAGTTCGAGTTTGTCAGGTTGGAAAATTTCATATCCAATCTAAATGATATCCGTAAGCCAAAATGCAGTTTCCTTGTGGAATCGGATGGTGAGA

>Rp-02

GGTTACATGCCACTTCACGTCACTGCCCGTTGTAGCCACACTATCCCCATTATTATTATCAACAACACAAACCAAACCACAGTCTCTTCTCTTGTATCTGCCTTACTTTTTGCAAAGAGAATGGCCACCACACCAGCTTTCTCCGGCGCCACCGTGAGCACCTCCTTCCTCCGGAGGCAGCCAGTGAGCACCAGCCTCAAGGCATTTCCCAACGTGAGTTCTGTGTTCGGTGTAAAAGGTGGACGTGGTGGTCGCATCACTGCCATGGCTGCTTACAAGGTGAAGCTGCTCACCCCAGATGGACCAGTAGAATATGAATGCCCAGATGATGTTTACATTCTTGACCAGGCTGAGGAGGAAGGTATTGACCTTCCTTACTCATGCAGAGCTGGTTCTTGCTCTTCCTGTACTGGCAAAGTTGTTGAAGGGGAGGTAGACCAGTCTGATAATAGCTTCCTTGATGATGAACAAATGAATGGTGGATTTGTTCTCACTTGTGTTGCTTACCCAAGGTCTAACGTTGTTATTGAGACCCACAAGGAGGATGACCTTTAAATGTCACTCACTACACTATCATTGCTTTTAAGTATCATCTTATTGTTCCTCTGATCATGTCTATCTGTTGCTGCGTTTAATTTTGTCAGGATATTATTATTATTATATATGGTTGTTTATGTTTCATCAGAATGAATGAATGAATGGATGAGTAACTTATTATGCATTTATTACTTCACCTGTAGTACCCAGTGAACTTCTTGTAGCCTTTTAATACTGTTTCCTCTTTGATGGATTGAATAAGAATTCTGATTTTCTCAGTAATTTATTTAGATGC

>Rp-03

GCCCCCCCTCTGCCCTCCTTACCAAATCAAAAACTGAAAAAAAATTAAAATTAAAATAATTTTTTTTCCCAGTTTCTCCGCCGTCACTTTTACCCTGTCTGCCGCCACTTCTGTTCGGTTCTCCGGCGGGTCTATTATCCTATTTTATCTTCTCCCTCAAGAGAAAAAGCAAAATCTAAAAAAAAAGATTTCTCAAAGTTATCTTTGTGAGAAGTGGTTAGGGTTTTTTAGTTCATCAGAGTTTTATAAGTGTTGATGGCGCAGATTCAGGTGCAACATCAAAGTCCAGTGGCCGTTTCGGCTCCTCCTCCTCCTCCTCCTCAGAACGGTGTTGCTCCCGCTCCGAATACGAACCAGTTCGTTACGACGTCGTTGTACGTTGGTGATCTTGACCAGAATGTCAACGATTCGCAGCTCTACGATCTTTTTAACCAGGTCGGCCAAGTCGTTTCGGTTCGTGTCTGTAGGGATTTGACGACCCGTCGTTCACTCGGTTACGGTTATGTTAATTACAGTAACCCTCAGGATGCGGCAAGGGCATTGGATGTGCTGAATTTTACTCCCCTAAACACCAAATCCATTAGGATCATGTATTCTCATCGGGATCCTAGTATAAGGAAGAGTGGTACTGCAAATATTTTTATCAAGAATTTGGATAAGGCAATTGATCACAAGGCTTTACATGATACTTTTTCTTCTTTTGGGAATATTCTTTCTTGCAAAATAGCTACTGATGGCTCTGGCCAGTCTAAGGGCTATGGTTTTGTTCAATTCGAGAATGAGGAATCTGCACAGAATGCAATTGACAAGTTAAATGGCATGCTGATCAATGATAAGCAAGTCTATGTAGGTCATTTCCTACGAAAACAGGATAGAGAGAATGCTGTTAGTAAGACAAAATTCAATAATGTCTATGTGAAAAACCTGTCGGATTCAATGACAGATGAAGACTTAAAGAAAACTTTTGGAGAGTATGGGACCATTACGAGTGCTGTTCTAATGAGAGATGCAGATGGTAAATCAAAGTGCTTTGGTTTTGTCAATTTTGATAACCCAGATGATGCCGCTAAAGCCGTTGAGGCACTGAATGGAAAAAAATTTGATGACAAGGAGTGGTATGTTGGGAAAGCCCAGAAAAAGTCTGAACGAGAGCTTGAACTCAAAGGACGATTTGAGCAGAGTATAAAGGAAGCTGCTGACAAATATCAAGGTGTGAACTTGTATCTCAAAAACTTGGATGATACCATCAGTGATGAAAAACTTAAAGAAATGTTCTCCGAGTTTGGTACCATAACCTCATGCAAGGTTATGCGAGACCCAAATGGAATCAGTAGAGGATCAGGATTTGTTGCATTTTCAACTTCTGAGGAAGCAACACGAGCTCTGGGAGAGATGAATGGTAAAATGATTGCTGGCAAACCTCTGTATGTTGCCCTTGCACAGAGAAAAGAAGAGAGAAGAGCAAGGTTACAGGCACAATTTTCACAAATGAGGCCTGTTGCAATTACTCCTTCTGTTGCACCCCGAATGCCCCTCTACCCTCCTGGCGCACCTGGTTTAGGACAACAATTTTTGTATGGGCAAGGACCCCCTGCCATGATGCCTCCACAAGCTGGATTTGGTTACCAGCAGCAACTTGTTCCAGGGATGAGACCTGGTGGTGCTCCAATGCCAAGCTTCTTTGTTCCCATGGTTCAGCAGGCCCAACAAGGCCAGCGCCCAGGTGGACGCCGAGGAGCAGGTCCTGTACAACAGCCCCAGCAGCCTGTGCCATTGATGCAGCAGCAGATGCTTCCAAGGGGACGTGTCTATCGCTACCCTCCGGGTCGCAACATGCAAGATGTTCCACTGCCAGGAGTAGCTGGAGGAATGATGTCAGTTCCTTATGACATGGGTGGTCTGCCAGTCCGGGATACCGTGGGACAACCAATGCCCATTCATGCTTTGGCTACAGCCCTTGCAAATGCTCCTCCTGAACAGCAGAGGACTATGCTTGGTGAAGCTTTATACCCGCTAGTAGATCAACTGGAACATGATGCAGCAGCTAAGGTTACAGGCATGCTTTTGGAGATGGACCAGCCTGAAGTATTGCATCTGATTGAGTCACCAGATGCTCTCAAGGCGAAAGTTGCTGAAGCCATGGATGTGTTGAGAAATGTTTCTCAACAGCAAGCCCCAGCCGATCAACTAGCCTCACTCTCTCTCAATGACAATCTCGTCTCTTAAGTTTCTGTTAGTCGGTTAACAGGAATTCACACCCTCTGGTCATTTCCCATCTCCTGATGTTCCCCACCCCACCACCCCATCTTCTGGTTTTATTTCCAGGTCTTTTAGACGGTACTTAGGAAGCTTGTTATCTTCTTTGTACTTGACGTTTTTGGTTTCTGGCTTGATGTAGGATTAGCAACACTTGTTGGATTTATTAGTTTATGGATTTACGGTTTGATTTATATTCTCCTCGGTACCGCAGGATTGTTTGTGTTATTGCTGGAATCTTTGATTGAGCCTTTTTTTCTGCCTTATAACTAATCGTGGTTTCCATGTTTTCCC

>Rp-04

CTCTCTCTCTCTCTCTCTCTTATATGTAAAGACAAAACCAAAAGCAAAACCAAAACCGTGGATCTGCCTCCTCAACCCTCCATTACCCATTCCCATCTCTCCCTCTCTCTCCCTCTCCTCCTTCATCCTCTTCCCCCAAGTGAAACCAAGGCATGATTATGCTTGAACCGTTGAGAATCATAGATCTGGCCGAAACCCACTTCGCCAATTGCTGAAATTCGAAACCTTTCTGATCCGATCAGAGAAATTGCGATCTGTTTCAACAAACCCAGATGAGTTCTCTACGCGCAGATCAAGGTGAACCAGATGGGTATGAAATGGACCACTTCGATCGCCTCCCAGATTCTCTTCTTCTTCTCATATTCAACAAGATCGGGGACGTTAAAGCCCTAGGTCGATGCTGCGTCGTTTCGCGTCGCTTCCATTCGCTGGTCCCACAGGTCGAAAACGTCGTCGTTCGTGTGGATTGTGTGATTTCTGATGACGATTCATCTTCCTCCGCTGCTTCCTCTGACAAATCTCGTGGCCCCTTCTGGAACCTTCTACGGTTAGTCTTCGGTGGAATCGCGAAGCCCATCCAGACTTTGGGCCAGTTCTTGGGCCCAAAACGGGCTTCATCTTCGGGCTCTTCTTCCTCTCCTCTCGCCGTCGGGAGCGAAGATGACGGCGACGGCGGCGTCACCCACCACTCTCCGACTCAGGTTTTGAAGAATTTCAACGAGATTCGGTTGCTTAGGATCGAGCTTCCAAGTGGAGAATTAGGGATTGAAGATGGGGTTTTGTTGAAATGGAGAGCTGATTTTGGATCAACTTTGGATAATTGTGTGATTCTAGGTGCTTCTTCTGTTTTTCAACCTAAATCTCGTGATGATGGTGTTGATGGTTCTTCATGTGGTGGTAATGGTAACAATGGTAATGGTAATGGTAATGGTAATGATGATAATGGAAGCATACCTGATTCGTTTTACACTAACGGTGGTCTAAAACTTCGTGTTGTTTGGACCATTAGTTCTTTGATTGCTGCATCAGCAAGGCATTACTTGCTTCAACCAATCATATCAGAGCATAGGACCTTGGATAATTTGGTTTTGACTGATGCTGATGGTCAAGGAGTGTTGTATATGAATAGGGATCAGCTTGAGGAGCTTAGGGTGAAGCCACTTTCTGCTTCTTCTGCTTCAAAGAGGACTCTTGTTCCTGCACTTAATATGAGGCTTTGGTATGCACCCCACTTGGAGTTGCCTGATGGGGTTGTTTTGAAAGGTGCTACTCTTGTTGCTATTAGGCCAAGTGAGCAATCTCCCTCCACTGCTAAGAAGGAGGGTTCTGATTTGTCTTGGGTTTCAACAGCATTTGATGAGCCTTATAGGACTGCAGCCACAATGCTTGTGAAGAGGAGGACTTATTGCCTTGAGATGAACTCCTTCTGATGCTTGCTTTGTGGTTCCATTCATTTAAAAGGGAATGCAGAAAATGGGGAACTTTAAGTTCCTCATAGAAGGAATCAGAAAATAGAAATTTTCTTTGGGCGTACATGAAGTGGTAGCAGTTCGGCCACCTGAGGAATCTTGCTCTGTCTGGCTTCTGAAACTGTGCCCCACATTTGAGTAGTTATTAAAAGAGCTACAAATCATTAACCTATCATGCTTGAACTGAAAACTGAATGCATCTGCTGGTTTTTCAAATTTGAGGCAATGCTTGTAAGAGTGCACGCCTGCAACTAGGATGATCTTGTAAGAGCTTTGAGTTTGTAGTCATCTGCCCTTTTCAGGTCTCCCTCTGTGAATATTCTAGGTATCAGTTTGGTTTGCTTTTGTGATATATTTTTCTGAAGTCAAATGAGCAATGTACTTTTGCTTC

>Rp-05

ATTATTATTATTAAGAACAGATGGCAAACGAGGGTGCGACCGTTTATCGTAAGGCAAATATTTTTTTTGACCGTGTACAATTACTTTATTGGCACAGCATAAAATACTCAATTGCTATTA

ATCAACTTATAAATCTGAAGGAGACATCATATATAAAGGCCTCCCGCAAAATTTCCCACGGGAAAGCCCGTCGGAAGCTTCTAAATCTTAGTGCACTTATGAAGGTTGACAGGTGCATAGGAACAGTGAATCCCAGATTCGTGGACCGTAGTTTTGTGGATTTACATTCCTCAAACGCAACGCAACGTTAAACCCTAAAATTTAAAACGGAAAGCTTGGTGTGCTATACATAACTTACCCAGCAAGGCACATATACAACATTTCCACGTTAGTCTGCTGCCCCACTAAAGATTAACTAGCGCTCCACATTTTGCATGGCTGTTTCAGTGTCAGTTAAACCTCCAGACATTGGTTGGCTAGTTCGACTTTCCTGCATCTATGCCATGGTGCCCTTGCACATTTATCCCAGAAATTCGTCATCAGGGTTCAACTGGCTCCGGCTCCTGTTCTGGCTCTGGCTCTGGCTCCGGCTCTTGTTCCTCTTCATCTGCAAGGGCGTACACCACTGCTGTTCGAGAAGGTGCATACACAAGAAAAGATCGAGGTCGGTCATCATACCATCCTTCAGCGGTGAAGTACTCAGCAGAGTGATTGAGCCTATTGAAGCCACCAAACAAGGCGTCATCTGAATCCAAGACAATCTTGTATTTCCCTGGGGTTAAACAGCCAACTCTGTAATCTGAATAGCTGTTGGTCCAATGAAAATTGAAGACAAAGATGAGGTTGCCCCTTTCAAAGACTATAATTTTGTCACCTTCATTTTTCCGGGAAATATATTGGTGCTCAGAAGTCATGAAACCAAACCTTTCTTCTAGATGCTGCATAGCTTGGTCAAATTCTTGCATCCCCCGATATCTTAGATAGACTGCATCCCCCAAGTCAAATCTACGCCTGCATTTATCGTAACTGTTGTTATTTCCAGGAACTACTTTCCCATTAGGAAGATGTTGATCACCCCTTGGAAAATCAATCCACTCAGGATGGCCGAATTCATTCCCCATAAAATTCAAATACCCTTCACCACCAAGACCCATAGTAATAAGCCTAATCATCTTGTGTAATGCTATACCACGATCTATAAGAGGAGTAGATGGTCTGTCTAAAGCCATGAAATCATACATATCCTTGTCCATCAACCAAAATGCAATTGTCTTGTCACCAACCAAGGCTTGGTCATGACTTTCAGCATAAGCCACACATTTTTCCAGCCACCTTCTGTTTGTGAGTGTGTGGATTATATCGCCCATTTTCCAGTCTTCATCTTTCTTCTTGAGAATCTCAATCCACTTGTCAGCAATTGCCATCTGCAGGCGATAGTCAAAGCCAACCCCACCATCTGGCGTAGGAAGGCAGAATGTTGGCATTCCACTGACGTCTTCACCAATCATAACAGCCTCAGGGTAGAGCCCATGAATGACATCATTGACCAGCATCAGGTAAACCACAGCATCAACATCAGTTGCATAACCAAAATACTCACTGTAATTTCCAGTAAAAGATACCTCCAATCCATGATGAGTGTACATCATTGATGTGACACCATCAAATCGAAACCCATCAAACTTGTATTCTTCCAGCCACCATCTTGCATTTGAAAGCAGATACCTTAGAACTTCCCAGCTTCCATAGTTAAAAAGGCGAGAATCCCACATCCAATGATAACCCCGTGACCCAGGATGGAAGTAATGACTATCAGTTCCATCAAACATGTTCAGCCCATCCAATGTATTATTTGATGCATGGCTGTGTACAACATCCATCAGAACAAGCAGACCTAGTTCATGGGCTCTGTCTATCAGAGACTTGAGTTCTTCAGGAGTTCCAAACCGACTGCTAGGTGCAAAGAAATTAGTAACATGGTACCCAAAGCTAGCATAATAGGAATGTTCTTGGATAGCCATAATCTGGACAGCATTATAGCCAAGCCTTTTAATGCGAGGTAGTACATCATCTCTAAAATTGACATATGTGTTGATTTTTGGCTCCGGACTACTCATTCCAACGTGTGACTCATATATTCTAATTGATTTTGGTTTCTTTGGCTGTGGATGTTTGAAGACATATTTTTCCTCCTCTGGGGGATCATAGTATATTCCATTATATGGAATTTCTCCAGGAGCCTGTACGGAGAACTTGATCCAAGCAGGAATTGAGTCCTTGATCCCAGATGGAGTATCCATGTGGATCTTGACTCGAGAACCATGAAGAATTGGTGGTGAACCATCCACATTGTTTGGCAAGAAGATCTCCCACACACCGAACTCATTCCGGGTCATTACATCTGCATTTGGATTCCAATTGTTGAAGTCTCCAACTAATGCTGCTGACTTAGCTCCAGGTGCCCACTCTCTGTATGTAACGCCTGTAGCACTGCGTGTGAAGCCAAAATTTTCATAACCACGAGAAAATGCATCCAGACCACCTTCATACTTGTCAATTTCTGCACGCAATCTTTTGTATTGTCCATAACGGAAATCAAGATGCTGGCGGTGAGCCTGCAAAAGTGGATCGATCTCATATATTTTCTGCCCAGTACCAGGTGGGGGAATGATCTTAGGTTTAACTTCATCACTTTCTATTGTTACTTTCCTATCTAAAACTGACGTCGTCTTGGCTTGAGCATTGGTGTTATCATCTCCAAGTGATGACATGACAGAATCTTGCCCATCCTCAACCTCTCTGTAACTGCCAGCTGCTTCATCAAGGTTGTACTTATTCTCATCTTTCATGGTTAAATCCTCTAAGTTCTGTGCATCCTCTGAGGTTATATCAAGTTGATCTGTCAAGGATGCAGAATCATCTTGATCTTGAGGAATAAGTCCTTTATCAGATTCAGCAATCATGGAAGATGTGGAGTCAGAATCATGGGAAGATTTTACAGAAATGGCTGTCCTAGCGAATGAATCCTTTCTGAGAAAAAATGCGAGAGGTGCAGTCCTGCGATCACCATGGAAACTCGACTTGTGCAGAGAAGGAACAACAGGAAATCGAATTCCAGAGATGGTGTAAACCATCTTCCTCAAACCTTAGGGGTTTTATTTCGGAGAGATTTTGTTTCAGAGAAAGGAAGAAAACAGATCCACAGTTTCGGAGTCTCTAGAAGAAAGTGTTGTTTTTAAGGTGCCGAGAGAAGCGTGAAACTGAGAGTCATGATATGAATGAGTAGCATTAATTTCTCGAAACAATAAAGGAATTTAAATAATAATAATAATGAGAAATGAATGAGGGAGAGATAGATTGAACGGTGAATGGTGGCGCCATCTGGGTGGAGGGACTATGTAACTGCAAGTGATGATGCTGAT

>Rp-06

GGGGGGGGGGATCAGGGACAAATTCATTTTATATATAGATTTAGAAACCTGTCATGACAAACAAAGGAAAAACATAAATATTTCAAGGACATCCTCAAGGCATTGAATTACTTGGTATTTTGTGCTAACTTTCCTTATGACACCTCTTTCTTCAGTTTCCTCCACTGTTTCTTTTCTCAAAATAAAGAACCAAAAATATCAGAGAAAAATAAAAGCCTCATAGTTATAACGCTCATATATAATCAGAACGCTGCCCATCCTGAAGCTGTGGTAGATCCTGAAGTGGCTGCGGAGGGAAGATTTTTCTGTAGCTGAGAAAGATCTGATAAAGAGTCAGTAGAATGTCTTGCACTTTCACATGTCCCTTTAGGACCAGAACTGTGAGCGGTAGAAGCAATCCGAGCAGCAACAGGATCATTTGGTGGGGGTGGAAGAGGAGACCTGATTTTCCCTGCCCCACTTGGTGGTGGAGCAAGGCTCAAAGTTTTTGGCTTTGGTGTTCCAGCATGGCCACTCGCTAGGCCAGCAGCTGAAAGCATGCCAGTTCCACTAGATGCTTTGTGCTTCACATTAATCCTGATGGTTTCACCTTCCTTGAGCCTGTGATTGACAGCGGGGTGAATATCGATCTGTGACTCCTCGGCGGCATCATCGTGGCCGGATTCCTTATCATGCTCTCGCCGGACGTACTTTTCGTGATCGGAAAGTGCGACGTTGAAATCGAAGGCCTCGTTCCTCTCATTGAACCCTAAACCAATGAACGCATGCTTTCCATGACCGTCCTCGATCTTCAGGACGAAGTAGCGCGACGAATCGAGGACCGGTTCGACGGAGGTCTCACGCTGGCCAGGGTACACAAAACACGCGGCGAAGAGGTCGGCGGAGTTGGGATCCTCCAACCGGATCTCACACCGGTCACGGCGAGACACTACACGGATCCGACCCGACCAGATCTTGTCGGATTGGAGCCACTCGCCGCACTTGTAACCGCCGGAGGTGGTTCGCGGCGGGATCTTGTAAACGGAGACCTCACGCACCACCAGAAGCGTGTGCTCGAAACCCTCATCCTCTTCCTCCAACGACATCGTTTCGATTTGAATTTGAGTTTGAGTTTGAGTTTGAGCTGCAATTTAAAGTGAATTTGATATAGGCGTTTGGATTCTGAAGAAGATTGGTGTGTTGTCTTTGTCTGTTT

>Rp-07

CTTTTTTCTCCCAACGAAACAAAGTCTTGTCAACCACCCCTAACACGGAGCCATTGCCGGTTTCGACTGCGCCACCTCTCTCTCTCTCTCTCTCTTCCGACCTCTCTTTCTCTCTCTTCTAGTCTTCACCTCCGTACAACACATCATGGGAGTGTTTCGCTGGTTTCTACCTTCTCCACCCTGTCAATGACCAAAACCTGCCGGTGCAATCATCTTTACTCTTGACCACCTTAACTTTTGCTACCTCTTTCTCCAACTTGGACACAACCAACATAAACAAAATCAAACAAAACTTGTTGTGATTGTGTGGTCCTTACCATTCATTACCAATCATCTCATGGCTATTTTAGGGTTTTAAGTTGTTGACAAAGTAGGGTTTTTCTCACCTTCCCATCTTCTTCCCTCATAGTTCAAGCTGAGTGAGAGAGGTTTTTTTTGGTTTCATTAATTGGAGTTGATGGAAGAATACGCTAATAATCACATGAGTGAAAACACAGGTCAAAGTCCAAGGCCAAATTTCTTGTACTCTTCTTCTAGTGCTGGTGTTGGAAACCATCATCATCATCATCAGCTTCCAATCAACACCTTTCATCTTCAATCAGGTGGTGGTTCAGATCACTGCTTTCAAACCGATCATGCACCACCATCACACCCTGCTGTGAAAACTGAAGCCACAACTTCACAGCTTCATACTTCGATTTTCCACTACCCCTTAATGAGAGGAAATCTTCACAACACTATGCATCATCCCCATCACAGTCAACAACAAGGAGGGAGTCCAAGCTCTAGTGAAGTTGAAGCCATAAAAGCCAAAATCATTGCTCATCCTCAATACTCTAGTCTTTTAGAAGCTTACATGGATTGCCAAAAGATAGGAGCTCCGCCGGAAGTGGTGGCGCGTTTGGTTGCGGCAAGGCAAGAGTTTGAGGCACGGCAAAGATCTTCAGCTGGCACAAGGGAATCTTCCAAAGACCCAGAACTTGACCAATTCATGGAAGCTTACTATGACATGCTTGTGAAGTATAGAGAGGAATTAACAAGGCCTTTACAAGAGGCTATGGATTTCATGCGAAGGATAGAAACTCAGCTAAATATGCTTTGCAATGGACCCGTTCGGATCTTCTCTGATGATAAATGTGAAGGCGTTGGTTCATCTGAAGAGGATCAAGACAATAGTGGTGGAGAAACAGAACTGCCCGAGATTGATCCCCGTGCAGAAGACCGTGAACTTAAGAACCATTTGCTGAGGAAATACAGTGGTTACTTAAGTAGCCTTAAGCAAGAACTTTCCAAGAAAAAGAAGAAAGGAAAACTGCCCAAAGATGCTAGGCAAAAGCTACTTAACTGGTGGGAATTACATTACAAATGGCCATATCCTTCGGAATCAGAGAAGGTGGCATTGGCTGAATCAACTGGTTTGGATCAGAAGCAAATAAATAACTGGTTCATAAATCAAAGGAAGAGGCATTGGAAACCATCTGAAGACATGCAGTTTATGGTGATGGATGGCCTGCATCCACAGAATGCAACTCTTTATATGGATGGTCACTACATGGCTGCTGATGGTCACTACCGTCTAGGGCCATGACCACCTTATATATATATATA

>Rp-08

GTCTCAGGTGCATAAGCTCATTACTTCTAGGGAAAATAAAATAAAATAAAATCCAAAGGAACACTGAATATAACCAAGCATCAATGTAAATTTGTTCAGATGCAAATATAACCAAGGAAATTTCTGATATACAATGTGCATTTCATCTGACAACCAATAACTGGGCATGACAGCCTCTTTTGTCCAAACAAGATCTTCAGAACATAAATGATCTAAAAGGAAACTGAAAGAGCTTCCCAGCTCAGCACAAACTACTGACTCATCTAGAATGCCATCAATCAATAGATTTAGACCTTCAATGCAATCTTCTGAGCTGCAAGAGCTTCAGCTTCAAGAGTTGGTTCCCACAATAATGTAGTCTCTGCAAGTAAAGCTGTCACAACATATGGATCCATGTTTGAAGCCGGGCGCCTGTCTTCCAAGTAACCTTTGCCATTCTTCTCTGTCTCTCTTCCAACACGGATTGAGCAACCGCGGTTAGCCACTCCCCAAGAAAATGAGTTAATGTCGGCTGTCTCATGCTTTCCTGTCAACCTTCTCTCATTTCCTTCTCCATATGCACTAATGTGGTCCAGGTGGCGAAGGGATAGATTCAAAATTGCCTTCTTTATTACCTCATAGCCTCCCTCTTCCCTCATGCTCTTTGTACTGTAATTGGTGTGGCATCCTGCACCATTCCAGTCACCCTCTATTGGTTTTGGATCAAGTGTGAGCACAACACCAGCTTGTTCAGTAATTCTCTCAAGGATGTACCTTGAAGCCCAGATATGATCACCAGCTTCAATACCTACACTAGGACCAACTTGATACTCCCACTGCCCAGGCATAACCTCCCCATTGGTGCCACTGATGTTAATTCCAGCATATAAGCAAGCCTTGTAATGAGCATCAGATATATCACGTCCAAATGACTTATCTGCCCCGGCACTACAGTAATAAGGACCCTGAGGACCAGGATAGCCACCCACGGGCCAACCTAGAGGCCATTTCACATTTGTTTGAAGTAAGGTGTACTCTTGCTCTATTCCATACCATGGAACTTCAGCTTGGACCTTTGGGTTACTGAAGACTTCAGCAGCTCTGTGTCGCTTGTTTGTAGGGATAGGCTCACCTGCTGGTGTGTAAGCATCGCAAATGACCAAAATATTGTTACCACCACGGAAAGGATCTTTGAAAATTGCTTGAGGATATAGGATTACTTCACTATCTTCACCAGGTGCCTGTCCAGTGCTAGATCCATCATAGTTCCATTTAGGGAGCTCAGAGGGATGCTCAACAGGTCTTGATATGGTTCTTGATTTACTGCGCACATCAACTCCTGTCCCTCCAATCCAGATGTACTCAGCAATTATCTTGTCAGTGTATGGAGTGATGTCCAAATTAAGTAGATTCTCTAGCCTGTTGATGGTGCTATTGTCAGACTTGATTGCCAGTACTCTAAATTTAGCAGAACTGCTACGGCCAACTTTCTTATTTTGTTTCAACAATAAAGAACTCCACATGTTTGATTTGATAGGACTTGCATTGGTAGAGGTTTTTGTGATTCTCATCTGCCATTGTGTAGAGGGTGCCAAAATCTGTGCCATGTCCAGTATACAGTGGGAAAATGGCTAAAGCTGCAAACTTTGGAGTGTGTTCAACTGAAAATGCACCCTGTGTCTGTGAGTGTCACAGACCCTGAGACAGAGTGAGAAGAGTGGTATGAGAGACTCGTGTAAGATAGGGGTTGATGGCCAAAGAAAAACTCTATTGGCTTTTCTAATACCGATAGGGGTATATTATGAGTGGATGAAGATTGTTTTTGGTCCCTTGAAGAACACCAAGAGAAACTAGGACTAGCAATATTTTAGATAAGGTTCTCAACCTT

>Rp-09

AGAAAAGTTACAGTTGTGCAATAACTGGCTAATTTTATTGCCTTAGGGTGTTCCATGAAACCAGTGGGATGGGACTTACAGATGCTAGTTTTGTTTCATATAAGATAGTCACCATGAACATGAACACTTATGTATTGGAAAACTACTGCCATGCTGGATTCTTCAAAGAAGTAACAGCAGCTTTAACTTGAAGGTAGCTGCTAAGGCTGTAGATTCCTTTTACTCTCCCTTGTCCACTCATCTTATACCCACCAAAAGGTATTGCTGCATCAAAGACATCATAGCAGTTGATCCACACTGATCCAACTCGCAATGCTCGCATCAAGGTGTTAGCAGTGTCCATATTCTGAGTAAAAACTCCAGCAACCAGGCCATAGGAAGTTGCATTGGCCCTTCTTATCACCTCCTCTAGATCCTTGAATTTTAAGATAGATTGTACTGGACCAAAGATCTCATCTATTGCTATCAACATGTTGTCCTGAACATTTGAGAAAACTGTAGGTTGGATGTAATAGCCTTTGGAGCCAATTCTTTCACCACCAGATTCAAGTGTAGCACCACTTTCAACTCCTGATCTTATGTATTTCAGGATCTTCTCAAATTGCATAGAATCAATCTGAGGCCCTTGCTCAACTCCATTTTTGAAAGGGTCTCCAACCACACGTTTAAGGGCACGAGCTTTAGCTTTTTCCACAAATTCATCATATATACTTTCATGAACAAATGTGCGAGAACCAGCACAACAACATTGGCCCTGATTAAAGAATAAGGCAGAATGTGCAGCTTCAACAGCTGCATCAACATCAGCATCCTTGCACACAATGAAGGGAGATTTTCCACCCAGCTCTAGAGTTACTGGCTTCAAATTGCTTTGTGCAGATAGTCCTAACACAATCTTACCAGTGCTACTGGATCCTGTGAAAGCAACCTTGTCAACATTCATATGACTGCACAAAGCTGCACCAGCAGTAGGACCAAAGCCAGAGATAATATTTAGAACACCAGGAGGAAGTCCTGCCTCAAGAAACAACTTTGACATATAAAGGGCAGAAAGAGGAGTTTGTTCTGCAGTTTTCAACACAACTGTATTACCACATGCTAATGCAGGTGCAACCTTCCATGAAGATATAAGAAGTGGGAAATTCCAAGGAACAATCTGCCCTGCTACACCAATAGGCTCATGCAAGGTTTGGACATGGTATGGTCCATCAGCTGGAACTGTTAGACCATGAATTTTATCTGCCCAACCCGCGTAATACCGAAACACACGAACCACCATAGGTATTTCAACATTTGCAGTTTGATCATAAGTCTTCCCACTATCCCATGTTTCAATTGCTGCAACTTCATCATTGTGTTTTTCCAATAAATCAGCAAAGCGCAAAATTATACGTGATCTTTCATAAGCTGTCATCTTTGGCCATGGTCCCTCATCAAAAGCCTTGCGAGCAGCATGGACAGCACGATTTACATCTTCACTATCACCTTCAGCAACATGAGCAATTATATCTCCTGTCCTAGGATCATAGGTTGGAAAAGTTTTTCCAGAAGCTGCATCAACAAATTGTCCATTAATCAGAAGCTGACTATGATCAATTTGCACAGGTGGTTTGCTTGGCTCCACATCAGCAGCAGCAGAAGCTCCAATTCCACTGATATTTCTATACCAACTACAATATAGGCCTAAACAACGTTTAGAAGCTGAGGCAGAAGAAGAGACATATTGCAGAGATGAAAGAATTCTAACAGCCATTATTATCTTCATATATGTTCTGAAAGTTGAACTTTTCTTTTCTTTTCCTTTCTTTCTTTCTTTCTTTCTGCTCCTGCACTAAGATATCTCAAGTCTTCTCCAAATGCTAACTTCACTTGATCTCTCACCACACTTCTTTTAAGAACTTGAAGTGTTACCTACCAGACAG

>Rp-10

AAGGGAACTAACTCAATAAAAAGGAGTGATGTTCCTTCCATCAATAGCCTCATCTCACGGATCTTGCCCCTCTCTGTGTTTGTGTCTCTCTCTGTGTTCCTCTCCCTCCCTTCTCACTTTTGTTTATTTTAATTCTCTACCATAGACATCATCTGTTGCTTTGCTTATTATATAAGGCCT

AGAGAATACTACATTCATTGCAAAGAGAAACTAATTGGCAGCATATTCAACAAAGGCATAATCTAGAGCTACCCAAATGGCCTCTTGGAAGAAGACCATCACAACACCATTCAAAAAAGCTTGCACTTTCTTTAACCAGCAACCTACAAGGGATCATCATAAGAAGTCTCAAACAGAGCAAGAAAGCCGTGTAATGGATCTGCATGGTGAAGTCATGGCATGTGGCTATGAAGATGTTCAAGTCATGTGGTCTATCCTGGACAGGTCAAAATCCACATCCTGCAACATCACTTCTTCAACATGAACCAACTCCTTCCCTTTCCTTTCCTTTCCTTTCTACCAATCAAAACAAGAAACCAAGTCCCACTGAAGAAATTAGCGAATGTTTTTGTATGCTAATGTTAAAGTCATGGGTGGTTGCATCATACTAGTTTATCTTAGCTGGCTAGTTAGCTGGCTTTTGAATGTGGTATGAATGTAATTCTCATTGTAGAAAATGAATAGATATTTTATATGTAGGTGTTTGGATCAGTGCCCCTTTTCCTTTGATGAAGATGGTGGCGGTTTCTGTTTGTAATGTTTTCCTAAGTCAAAATGTTGGGAATCCCCGTGATTGATGAAAGAAAATCAT

>Rp-11

ATAAAAATCAAACTTAAAGATCATCAGAACATAATATATGGTATTCAATCCAAAATAGCTACGATATCAGGTGATCAATTAATTTACTTTTGCTAGCTAAGATCCATCCCGTCCGGTGAGAACTGAAAACAACTGTTCAGGCTATGCCGTCAAGAAATTAATCTGTTCTCTCACTAACTCATTTAAAAATTCTTTCAAAACAAGTCCAACAACATTTGCTCTAACTAGGTAAAAACTGACCATTGACAGAATATCACGCTCTAGCCTCCAAATTTTCTGACCACAAATGTACTAATCTGCCAATGATTAACGCTTCTTCTTGCTTCCTTTTGCACCAGATTTTGATGGTGTAGCTACAAGGTACACAAACAGAACCATGATGGAGATCACTGAGATTAGAGATCCATATTTAGCCAGCAACCTCTTAGCCCATTCAAACTTCTTCTCTGGAGGTCTATCAGCAAGAACATCTAAAGGCAATATGGGAGATGAATATGCCTCCTGTAGAGCAGGCTTTGTGGGGACACGGAACTTTATGACAGCTGGTTCACCAGAAAATACTCCCTTCGTTTTTGCCTCCAGCTCAAATGTGTGGGAGAGGATGCCACCAGCATCAAGCTTTTCCCATGACTTTGAAGTGCTACCGCTGATTACATCGAAAACATCACTTGGCCAACTATCATCTGCCAGACTTACATCGTATGCGGTCGAGGTTCCTTGGTTGTAGATGTCGATGGTGACAGATACCCTTTCAGCACCAGATTTGAGCCTGTTGAGTGATGCCTTCTTGTGAGCGACGATGAACGGTACGTCAGAGGAAGCGAGTGAGCAAACCAACAACGAAGCTATCACCGCAAATGAAATCAGAGACTTCCAGATTGGATCCGCCATTGGTACCCGAAGAGAGAGAGAGAGAGAGAGTCAATGAGGAAGAGAGAGGAGATCGTGGGACTCTTTTTATGTTGATTTGTGATCTAGGACGCACTTCGACCACTAGGG

>Rp-12

GTAAAATGAACTTGCAAAGTATACGGCGTACTTTCCTATCACATGTCCTCTGCATGGTCCAAGCCAACCACATTGCTTTCCATAAGGGATATGAAGCCGTAAGACTATTTCAGTGGATCAATCTTCACGAGAAAATGCAACTTACTTTGCGTGAAGGCTTTTTAACTCGATAAATAAATATAGCTTAAAATCCATTACCTCCAGCAAGGCACCACACTAGATCAAAATGTCAAAAGAAGCAATTCTATGCTGCCACAGATCCTGAAGAAGCTTTCCTTTGCTCTAAAAGACTTGAGACTGCACGATCAATTTGAGCAAATACTTCCTCCTTTGAGACATTTCCATCAATCTTAATAGTTATATCTTTATACATGGAAAGGACTGCCTCCACATTTTGATGATGGGTGTTCAATCGCAACTTTACCTTTTCTTCAGTATCATCAAAACGTTGGGTAAGCCTTTCTGCGATTTCTTTTGTCTCTGGAGGAGAATACTTCAAGTGATATATCTTCCCAGTAACAGGATCTAATCTCCGTCCAACTACTCTCTCCACGAGAATATCTTCAGAGACCTCTAGAAGAATAAAGATATCAGGTTGGAACCCAAATTCGTGAAGTGCAATAGCCTGTGATAAGCTCCTGGGATATCCATCCAAAAGCCAACCATTCTCTATAGAATCTGGCTTCGTGAGACGCTCCTTGACCATCATGACAACAATTTCGTTAGGGACCAACTGTCCATTCTCCATGTATTCTTTAGCTCGCCTTCCATTTTCACTTCCAGTGGCAATTTCAGCCCTAAGCAAATCTCCAGCAGCAACATGCACCAAACCGTATTTATTGGTGATAAGCTGACATTGGGTTCCTTTACCAGAAGCGGGAGCCCCTGAAATCATAATATGGAGCGGCTCCAACTTAGCCTTAGCCTTGACAATCACATAATCAGAATGAGAATGGAGGGCGTTGGGAGTGAAAGGAGATCGGGAAGAGTCATCACGGAGACCAAAGGCATTAGCAGAAGCAGAAGCAGAAGCAGAAGGAAAGGAAAGTTGTTGATGCCGCCGCGTAACCCAAACTGAAACTGAAGATGAAGATGCACGCTGCTGCGCCTTACTCTCGCACTTAATTGGACTCCCCATCCATGCTGCTGCCGCATTCAAATTCATTT

>Rp-13

TTTAAGTTCAATGGATACAGTGGTTCCCGGCGTTGGCATTGGCCTCAGCCTCTGCATCCCACGACTCTCACATTTCACTCGAAAACCTAACAAACTCTATTCCTCCGTCACCACCGGCGGTTCAATTTCCGCCACCAGCAAATGGGCCGAACGTCTCATTTCCGATTTCCAATTCCTCGGCGACTCTTCCTCTTCCTCTTCCTCTTCGGTCGCTAATAATCCAAACCTTTCTTCTCTTCCTCCTCCGAGACTCGACCCTCCCGAACGCCACGTCACCATCCCCCTCGACTTCTACCGAGTCCTCGGCGCCGAAACGCATTTCCTCGGCGATGGCATTCGCAGGGCTTACGAAGCAAAATTCTCGAAGCCTCCACAGTACGCTTTCAGTAACGACGCTTTAATCAGCCGTCGCCAAATCCTCCAAGCTGCATGTGAAACCCTAGCTGACCCTGCTTCTAGAAGAGACTACAATCAACGCCTCCTCGATGATGAAGATTCCACCATTCTCACCCAAATCCCTTTCGACAAAGTTCCTGGGGCTTTGTGTGCGTTGCAGGAAGCTGGAGAGACTGAATTGGTGCTTGAAATTGGAGAGGGTTTGCTTAGAGAGAGGTTGCCGAAGACGTTTAAGCAAGATGTTGTGTTGGCTATGGCACTTGCTTTTGTTGACATCTCAAGGGACGCTATGGCTTTGTCACCACCTGATTTCATAGCTGCATGTGAGATGCTCGAGAGGGCATTGAAGCTTTTGCAGGAAGAAGGGGCAAGCAGCCTAGCACCAGATTTACAAGCACAAATTGATGAGACGCTTGAAGAGATAACCCCACGTTGTGTTTTGGAACTTTTAGCCTTGCCTCTTGATGATGAACATCGAGTGCGGAGGGAGGAAGGTCTTCATGGTGTCCGCAACATTTTGTGGGCGGTTGGAGGAGGGGGAGCGGCAGCAATTGCCGGGGGGTTTACCCGTGAAGATTTCATGAATGAGGCATTCTTACATATGACAGCAGCTGAACAGGTTGAACTTTTTGTAGCCACACCGAGTAACATTCCAGCTGAAAGTTTTGAAGCATATGGAGTGGCACTTGCACTGGTTGCACAAGCCTTTGTAGGTAAAAAGCCACATCTTATCCGAGATGCTGATAACTTATTCCAACAACTTCAGCAAACTAAGATAACAACCGTAAGGAACGCTTCCTCTGTTTATACTTCTAAGGAGAAAAGAGAGGTTGATTTTGCTTTAGAAAGAGGCCTCTGTGCACTGCTTGTTGGGGAGCTCGATCAGTGTCGATCATGGTTGGGACTAGATAGTGATAGCTCTCCTTATAGAAACCCATCTATTATCGACTTCATTATGGAAAATGCAAAGGGTGATGAAGACAGTGATCTTCCTGGACTCTGCATATTGTTGGAAACATGGTTGATGGAGGTGGTTTTTCCAAGGTTTAGAGATACCAAAGACACAAGATTCAAGCTTGGAGATTACTATGATGACCCTACAGTGCTGAGATATCTAGAGAGGTTGGAGGGTGTTGGCAATTCACCCTTGGCTGCTGCAGCAGCCATAGTAAAAATCGGAGCAGAGGCTACTGCTGTTATTGGTCATGTCCAGGCTAGTGCAATAAACGCATTGAAGAAAGTATTTCCTGTTGGCTCTGACGATCAAATTGAGAAACATGAAGAAAATGGTGAGAAGGATTATACCGACCTTTCTGAAAGTGAGAATCCTCTGATATTAGCAGATCAGGATACTTCAGTCAATGTTGAGGTTTCTGGAATAAAAAATACTACTGAGATAAATGATGGTGAATTTATTACTGACGAAATTAAAAATGCAAGC

>Rp-14

ATTTATAAAATGAAAGATGGTGTAATTACAACCAACCTCAAAGGAGGTGAGTGTAATTTACCCAAAATAATGTAGTTACATGTTAACCGAATCTGAATTATGTTTTGCGGGCCAATAAAATCTGCAACTGTGGCAGGAACATTAACAAGTGATCTTAACATATACATGACCGTATATTAGTCCAACAAGTACATAACAATTGCTTGTAAGTGGCGAAAGATAAGAATGTTTTCCAGGTTCATTCCTATTTTCCCTCAGCAGCATAGAGAGCTTTGATTTCTTCGACATCGTCATAGCTACCAAGGAATATGGGAGACCTCTCATGCAACTTCTTGGGAACCAAATCAAGTGCCCGTTGCTTGCCAGTGAAAGCCTGTCCTCCTGCCTGTTCAAGCAAGAATGACATTGGGAAGACTTCATAGAGAACACGTAGTTTCCCATTTGGACTCTTTTTATCAGCAGGGTATAGAAAGATACCACCATAAAGCAATGTGCGGTGAACATCAGCTACCATGCTTCCAATATATCTTAGCGACTTTGGTGATGAACCATCTTTTGGAAATTTGCATTTCTCTACATACTTGGCAGTAGGACCATCCCAGTTTTTAGCATTTCCTTCATTCACTGAATAGATCTTGCCTTTCTTTGGGATCTTGATGTCAGGGTGAGTTAGAATGAACTCCCCAAGAGATGGATCCAGGGTGAAACCATGAACACCAGTTCCAGTGCTTAACACAAGCGTGCAAGAGCTTCCATACATACAATAACCAGCTGCCAACATGTTCTTCCCGGGTTGCAAAACATCTTCTATGGTTGGTTCATGGTCTTCGTTCACCATATAAATTCCAAAAATTGTGCCAATGGATACACCACAATCAATGTTTGAGGAACCATCCAATGGGTCAAAAGCAACACAATACTTTCCCCGCTTAGAAGACTCCACAAATGTTGCCTCCTCATCTTCTTCAGATACCAAGATGCATGTTCGCCCGCTGCTTATCAAAGCCTTGATAAAGACATCATTGGAAAGGACATCCAGTTTTTTTTGCTCTTCACCCTGAACATTGGTCTCTCCAGCCAGTCCAATAAGCTTAGCAAGACCCGCCTTGCTAACAGCAGAGCAAACAAACTTGCAGCCAAGAACAATGTGGCTGAGCAAGATGGTGAAATCTCCACGTGACTCAGGGTGCTTGGACTGCTCGTTTAGCACGAACCTGGTTATGGTCATGAGGTCCGTGCGTTGTGCATCCGCGCTGTGATCCATATTTGTTTGTGCTTCTGGTGCAACTGCAACTGCAACTGCAACCCCTTCTTCTTCTTCTTCTCTCTTTCTCTCAAGGAACCAAATGAAGTGAGTT

>Rp-15

CGAGGAGTGAGAGTGAGGGAGACGATGAGTATCGTTTGGAGAAGGAGCTAAGAAAGATAGGAGAGAGAAGATAAACAACAGAGCAGCGGTGATGCCAGAAATGACTGGAATAATAAGGCGGTAATAATGGTGGCGCCGCCGCTGAAGTGCCATTAATTAACTAATGCGGCGAGAAGAAGGAATGAGGAGGAGAAGTTGAAGGAGTGAGTGGCGTAGCATATCACTCACTCACTCACTCACCGCACACACTTTACTTGTTTCGCACACTTCCTCTC

>Rp-16

ATTTATTGAAAGTGTATATGCTATATGTCATTGTCATGGTACATTGAAAGTGTGTGTACTATTTAAATAATGCTACAGACATTTGACTGCCATGAAACTAAAAATAAACTGCTACATCCCAATACTATGACTTAAACATAGATCTTATTTGATATTCAGATTCAATCTTTATTAGATAAAACATATATTATACATATACGTATGCATCTACTACTTCATGTATTAACAACCAAGTGCATTGAAGGTTCACTCACAGATCCTCATGGGGACAAGCTTCAAGAAACTCTTTTATTCCGAGTCTTTCATCCTTTGTGAGATCAGGATCATCACCAGTCAATCTCTCACTAAGGGAAGCACGAACACCGCTGGTGTCATCAAGGACTTGAAGAAAATTGTTGACATGGCCATCATTGTTGGGAGAGGTTACAAGCTCATCCTTGTTGCATGTCCATTCCCCATCAACAATGTATTTGTACTCGTAGCGTCCTTCGGGCAATTCCCTCTCGAGAAACCACAAACCCCGTTTGTCATCAAAATTTAGGGGCATTCTCTGCCACCATCCAATGTCAAGTCCTGAAATTTCCACCGTAGAGCAATTGCTGTCTTCCCATGACAAAGTGACAGGCTTCTTACTAAAGCCTGTAAGAATATCAGCCGTTGCACTTTTTATGGCATCCAGTTTTGGAAAGCATGACCTTTTGCTCTGAAGTAGTGTATGACCGTCATTAAGTTTATAACCCAAAACCCAAAACATATATGCCAACGCCACAGCTGGAGCTCTTCCAAGTCCAGCAGTGCAGTGTATATATGTCACACCTCCATTGGAATTTATTGCCTTGTATAATTTGCTAACTACAACTGGAAGCCGGAACCGTAAATCAAATGCATCAAAGTCCCTTATCTCAGCACGCAAGTGTTGAATGTCATTGCATGTCTTGGCATATTCTCGGATGGCATTGATATCAACTCCAAAATATTCTAGGTCTGGATCTTGTTGCAAGCAAAATATAGTTTTCACTCCGATTTTACGCAGCTTGTCAACATCTTCAGGAGTCTGTAGGCATGATCCCACGATCAAATCTGGACGGATGAAGTTGTAGTTCATTCCTAATTCATGCCTATAAGTCAAAACAGCACCCATAGCTTCTGTCATGTTATGACTATATGTCTCAGACTTTTCTTCCTTCACATTGGAGCTACTTGTCTCTGCACCAGGAATAGAACCAGAAACTGACTTAAGTGCCATGCTTCGATTTGGATGAGAATTATTGAAGATTCCCGGGGAAAGAGAAGAGAAAGAAAGATTATTGGGGTGGTGGCGCGTAACAACTGCTTGGAAGGGCAAAACAAAGGATCGAGGAAGATTGTGAAGACAGTTCATGATTATGAGACAGTGTTGGTTGGTTGAGCAAACTTCAGTTTCAGTTTCAGTTTCAGTTTCACAAGCTAAAATCGTCACCACACTGAACGAGATGATGAAGAAGGAACCCGAGTCGACCCGAGTGAGGGAGCGAGTCGACCAGGACGACACGGCCACGCTGTTATACTCTTCTGGCACGACGGGACCCAGCAAAGGCGTGGTTTCGTCGCATAAGAATCTGATGGCGATGGTCCAAATCGTTTTGGGTAGGTTCAGTAAGGAAGATGAGCAAACCTTCATTTGCACGGTGCCCATGTTTCATATATACGGTTTGGCGGTGTTTGCGACGGGGCTTCTTGCTTCGGGATCAACCATCGTTGTGCTGTCTAAGTTCGAGATGCACGATATGCTGTCGGCGATTGAGAGGTTTCGAGCCACGTTTCTTCCGCTTGTGCCGCCCATACTGGTGGCGATGATCAACAACGCCGATGCGATTAAGGCCAAGTACGATTTGAGGTCTCTGCATACAGTTCTCTCCGGTGGGGCTCCGCTCAGTAAGGAGGTGACAGAAGGGTTTGTGGAGAAGTACCCCAACGTTACTATTCTTCAGGGTTATGGCTTGACGGAATCATCTGGAGTTGGGGCTTCCACCGACTCATTGGACGAGAGTCGAAGGTACGGCACGGCGGGGTTACTTTCCCCTGCCACCGAGGCTATGATTGTTGACCCTGAAACCGCTCAACCGCAGCCGGTTAACCGGACCGGTGAGCTCTGGCTTCGGGGTCCCACCATCATGAAAGGTTATTTCAGTAACGAGGAAGCAACCTCATCAACCCTTAATTCAGAAGGATGGTTAAGAACAGGGGATATTTGCTACATTGATGATGATGGATTCTTATTTGTTGTTGATCGGTTGAAAGAGCTCATTAAATACAAGGGATATCAGGTCCCTCCAGCAGAACTAGAGGCCTTGTTGCTGACTCATCCTGCTATTTTGGATGCTGCTGTCATACCGTATCCAGATAAGGAGGTTGGGCAATATCCAATGGCATATGTGGTAAGGAAGGCTGGAAGTAGCATATCAGAAAAAGAAGTTATGGATTTTGTTGCAGGACAGGTGGCTCCGTACAAAAAAATTCGAAAAGTGGCTTTCATATCTTCCATACCCAAAAATGCATCTGGTAAAATTCTTAGGAAGGATCTGATCAAGCTCGCTACGTCTAAACTCTGAACACTGGAGTTTTCTAATACTTGGTTTCTATTGTAATTGCAGAAACAGATGATTCTTTATCTAATTCCCTTTTTAGCTCTAATTTTATGGCTTGTTTATTTTTGTTGATATAATATTAAATGCGCTTTTTTCCCGTTCTGTGTTGACAGTCTATTTTGTAAAGAGCAGCAATATTGCTGAGTGCTCCCTAATAATACTCTTAC

>Rp-17

TTTCTTTGGAAGAGAGGATGCTACAGATGAAGAGGTTGTGGAAGCTGCCAAAGCTTCCAATGTTCATAATTTCATTTCACAATTACCACAAGGATATGATACTCAGGTTGGAGAGAGGGGAGTTCAAATGTCAGGTGGACAAAAACAAAGGATTGCTATAGCAAGAGCCATAATAAAAAGACCAAGAATCCTCTTACTAGATGAAGCAACAAGTGCACTTGATTCTGAATCAGAAAGAGTTGTTCAAGAAGCTTTAGACAAAGCTGCAGTTGGACGCACAACCATCATCATAGCTCACAGATTATCCACAATAAGAAACGCAGATGTCATTGCTGTCGTTCAAAATGGTAAGATCATGGAAATGGGGTCACACCACAACCTCATTCAAAACGACAATGGTCTTTACACTTCCCTAGTTCATCTCCAACAAACCAAAAATGAACAACAAAACGACACCACCCTCTCTCATAACAACACTCATCTTCACCCTTCATCTATTTCAAATATAGACAATGTTCAAAACACAAGTAGTCGTAGACTCTCACTTAATATAAGTCGTTCTAGCTCTTCAAACTCAATGGCACGTGATTCTTCTTCAATTCCAAATGCTGATGATGTTGTTGTTGTTGAAGATAATAAGAAGCTACCTGTTCCTTCATTTAGAAGGTTGTTGGCTATGAACATACCAGAGTGGAGACATGCATGTTTAGGGTGTTTGAATGCTGTGTTGTTTGGTGCAGTTCAACCTGTGTATGCATTTGCAATGGGGTCAGTGATATCTGTTTATTTTCTCAATGATCATGATGAGATCAAGAAGCAGATAAGGATCTATTCACTTTGTTTTCTGGGGTTGGCTTTGTTCTCTTTGGTGATTAATATACTTCAGCATTATAGCTTTGCTTACATGGGAGAGTACTTGACAAAAAGGGTCAGAGAAAGAATGCTTTCTAAGATACTTACTTTTGAGGTTGGATGGTTTGATCGTGATGAAAATTCCACAGGTGCTATTTGCTCTAGACTTGCCAAAGAAGCCAATGTGGTAAGGTCTTTGGTGGGAGATAGAATGGCTCTAGTGGTACAAACTATCTCAGCAGTGGTAATAGCCTTTACCATGGGCCTGATAATTGCATGGAGGTTGGCCATTGTTATGATAGCAGTTCAACCCATTATCATAGTCTGCTTCTACATAAGACGTGTCCTTCTTAAGAGCATGTCAAGCATGGCCATAAAAGCCCAAGATGAAAGCAGCAAGATAGCTGCTGAAGCTGTTTCCAACCTTAGAACCATCACTGCTTTTTCTTCCCAAGACAGAATCCTCAAAATGCTTCAGAAAGCCCAAGAAGGCCCAAGCCATGAAAGCATTAGACAATCTTGGTTTGCAGGCATTGGGCTTGCATGTTCCCAAAGCCTAACATTTTGCACTTGGGCTTTGGACTTTTGGTATGGTGGTAAGCTTGTGTATCATGGGTATATCAATGCCAAAGCACTATTTGAGACCTTTATGATCTTGGTCAGCACAGGTAGGGTCATTGCTGATGCTGGTAGCATGACCAATGACCTTGCAAAAGGAGCAGATGCTGTGGGCTCAGTTTTTGCAATTCTTGATAGGTACACAAAAATTGAGCCTGATGATTTAGATGGGTACAAGCCTGAAAAGCTAACAGGGAAAATAGAACTCCATGATGTCCATTTTGCATACCCAGCTAGGCCTAATGTAATGATATTCCAAGGCTTCTCAATCAAAATTGATGCAGGCAAATCAACAGCATTGGTGGGACAAAGTGGTTCTGGCAAATCAACAATCATAGGCTTAATTGAGAGATTTTATGATCCACTTAAAGGGACAGTGGCCATAGATGATAGAGACATAAAATCATATCACCTTTGGTCACTAAGGAAACACATTGCACTAGTGAGCCAAGAGCCAACACTTTTTGGTGGGACCATAAAGGAGAACATTGCATATGGAGCATCTAATAAGGTTGATGAAAGTGAGATCATAGAGGCAGCAAGGGCTGCAAATGCTCATGATTTCATAGCAAGTTTGAAAGATGGTTATGACACATGGTGTGGTGATAGAGGAGTGCAACTATCTGGGGGTCAAAAGCAAAGGATTGCAATAGCTAGAGCCATATTGAAGAACCCTGAGGTGTTGCTTCTTGATGAAGCCACCAGTGCCCTTGATAGCCAATCAGAGAAATTGGTGCAAGATGCTCTAGAAAGGGTGATGGTGGGGAGGACTAGTGTGGTGGTAGCCCATAGGTTAAGCACCATACAAAACTGTGATCAAATTGCTGTGTTAGATAAAGGAAGGGTGGTGGAGAAAGGGACCCACTCTTCTTTGTTGGCTAAGGGACCAAGTGGAGCTTATTACTCATTGGTAAGTCTGCAAAGAAGACCAACCAACACTGTTGCTGACTCTGCAAATGAAATCAACTAACCAAACCAAACCAAACCAAGTTGCCATGTCTTGTTTGAGATTACTATAAAGACAAGAATGATGGGGCTGTTGAGTTGATAGGTGAAAAGCAAATTGAGCCAAGGCAAAATTTTCCATATATATATATGCTGGTGGTCCCCAGAGATAGCAC

>Rp-18

AAAGAAGAAGAAGAAGAAGAAACCCTAGAGAAAGAAAGAAAGAACCAACTCAACTCATCATTCAATACAAATTCAAATTTTCATTTCCATTCATAGCTTTCATTCAATTCCATTCCATTCTTTTCTCTTGCCACTGTTTCAATTTTCAACTCCTGAGATTCATGTGGAAGTGGTTTGAAGATTGCACATTTGAATGGAAGTAGTGGTGCAATTGCGATTGTTAACCGTTTGTTTCGGAGAGGCACAGTAATAAAGGCTTGGACTTAAGGGTTGCTTGTTTCTCACAAGGCTTTGTGTGGTGTAATTGGTAATTTGGTTGAACTCATGTTTTGTGAAGTTAGTTATTGTTGTTGTGCTTAGGTGAGTGATTGTTAGTAAAGAATGGTGCCTCCTGGGCCACCCAATCAGATTGGTGGTGGCCAGTCTGTGTCGCCTTCACTTTTGAGATCGAATTCTGGAATGTTGGGGGGTCAAGGTGGTCCTGTGCCTTCACAGGCTTCATTCCCTTCGTTGGTTTCACCACGAAATCAGTATAACAACATGAATATGCTAGGAAACATGTCCAATGTTACTTCCATACTGAACCAGTCTTTCCCGAACGGAATTCCAAATTCCGGGCTCTCTGGTCCCGGAAGTAGCCAGTGTGGTGGTGTGGATTCCAGGGCGGAACAGGATCCACTGTCCGGTGTTGGCAATGGGATGAACTTTGGCAATCCTTCGTCGTTGTCGTTTGTGCAATCGAATGTGGTGAACCCTGGTTCATCTGGTCAAGGTCAGGGTCAGGGTCAGGGTCAGCAATTTTCAAATCCTTCTGGTAACCAGCTGTTGCCGGATCAACAGCATTCCCAACAAATTGAAGTTCAAAATTTCCAACATAGTCAGCAGCAGTCAATGCAACAGTTTTCGACCCCTCTGAATACTCAGCAACAACAGCAGCAGCAGCATTTTCAATCGATGCGAGGAGGGATGGGTGGTGTTGGACCTGTGAAGCTGGAGCCCCAGGTAAACAATGATCAGTTTGGGCAGCAGCCACAGCAGCAGTTGCCATCGTTGAGGAATCTTGCTTCGGTGAAATTGGAGCCGCAACAACTTCAAACAATGAGAGCTCTCGCACCAGTAAAAATGGAGCCTCAGCATACTGATCAGCCATTTTTGCATCAACAACAGCAGCAGCAGCAACAA

>Rp-19

CTCGGGCACAAGCTTAGCCACCTTTTCCGGTATTTGCTTCTCCGGGGAATTCCTCTTCCTCCGCAGAGGAGCTGGGGTCAACTCCATGGTCTTAAAAGCTGAGGTTTGGTTAGCACTGGACGAACCTTGAGGCCGAGAAGGCGGCTTCTGAGTCGCACGCTTGGCAGTAGCGGTTCCAGTTGCAGGAGTGGCAGCATTAGTGTTGTTGGCATTATTATGCAATTGGTTCACAGGCTGAGGCTGAGGCTGAGGCTGTTGCTGAGTTTGAGGGTGTGCACGAGACTGTGCAAAATGTGCTTGTTGTGCAACCACATGGGTTTGGGGTTGTGAAAGCTGAAAATGACCGGGGAAGTGAGAAGCACCTAGGGTTTGGGGTTGAGATTGAGTGAGAAGATGAATTGGTGGTTGGCTCATTGGGATCGTTGAATTGCCGAAAAACGACGGCGTCGCTGCATTCTTCGCTGGATTATTATTGTTCATTATTCTTCGCTGAATCTAAACCTCGATACTTAAAATTGCTTCTAGGATAACAAACAATTATTGGATCTAAAAGTATTCCGGAAATTTGAAGAACGAAAAACCCTAATTACAAAACGACAATCCTGAAAAAGTGAATTGGG

>Rp-20

CTAAAATGAAATGGTAGATTTCAGTAGGAAACATAGTAACCAATAACTATTATCATCTGG

ACACTTTACTATTCAAGTTCTATTAGCAATTTGATGTACTGTTATGATGGATCAAGACAA

TTAAGTACAAACATATGATTTCATTGCTCTTGGTAATTGACATCATGATCTTGGATCTCA

CCAGAGCTGAGAAGTGAGTTGACCCTGCTGCCTTCTATTTCATCTTCAGATTTGGATTGTTTTGAGATCCTCTCCACTCCATCAGAGATGTCTGAAGAATGAGTTACTGGCTGATCATCTGGCATCAGTTCTTCTTGGACCACTGGCTTTGGGCGTCTGATTGCTTCTCCAGCCTCCGGCATAGCAATGGGTGGGGATTGAGGGGGAACCCACTTACGTTGGACTGGCTGCAGACGGGATGCATAAGGTGCTCCATTATACTCAATTCCATTATCAATCTCTCTGATTCTGACATTTTTCCTCTGCCACCAGGACAATGGGTCATCCCCATTTGAATGAGTGGTATCTTGGACCTTGGTATTCAAGTCTTCACCATTTACTTGAGACTGAAGCACTTGGGTAGAAGTGTTTTGCACCTGACTAACCTCCCAAGGTCTAATATTAGAAGGCTTCTCCCCTCTTTGGGCCATGGCCATCATCTCCATATATGACTTTGGATGAGGAGCACTTGATGGTTCAACCGATGCAGGAGGGGATAAAGATCTCACTGAACGCAAGTCATAGTCTGCCTTGCCATTCATAATCAGTTGCTTCGAACTTGAATGACTGATTCGGAGATCTTCTTGCCCTTCCAATCTTCTTATTGCATTTGTCATTAACTTCATTTCTTGTATTTGCTTATCTAAAAGGTTCACAACTTCCACAAAGTATTTCCTCTCTTCACCCTTTGAAGCCAGCATTTCTTGGCTTGCTTTTGCTACATCAGCAGCTGCAGCTGCAGCTGATTTTGCAGCTTGTGCAGCTTCTTCCGCCAAAGTTGGTTTGCTGTCAGTTTTCTTTAATTGTTCATCATCTTCATCTAAGACAACCTTGCGTATCCAAGATTTTAACCGAGGAAGAATGGAATTCTTAATTATTAAAGCAGTTCCAGCACCTGAAGCAGCCAGTAATCCAACTGCAATAAGGGCATGGGACCAGCGAAATCTGGATCTTGCTAAGGTTCCTGATGAAGTATTAACAGCAGTAGAGGCAGGTAAACCAGGCTGCAAGGTTTGTTGTTGGGCCTGTTGCTGAATGTTTGATGATGATTTCAATTGTCCATCTTGATTTACACCAGCAGTCTGCACAGTCGGAGCCGAATCTGGCACACGCCGAAAGGCTTCATCAATCTCCTCCTTTGTAAGGCCCTTCTTCTCCAGAAATGACCGCCTGTAGATGACAGGTGAACCTTTAACTTTTGGGTGTGAAAGAAATTTCACAGCATTCTGAACTTGATCTTCTCGCAATGGTTGCGAGTTCACAAAGACTGACGTAGTTGAACTCTGTTTAACAGGCTCCTCTCTGGCATTTTGCTGATCCACATTTGTTGGTTGCACAATTTCAGCACCTTGGTTGGGAAGGTTTTCGTCGGTGGCGGTGGGAGAGCGAGTAGGTGGCGATTGTGTGGCCATGTTTTTCTATTCGCACCTGCTTCTCACTCCAGTATTACAATTCAGTGAAGATGACGACGAAGTGAGGAGTGAGTGGATTAGCTCAAGAAAAGAAAAACAATTGGGGTTTTATTTCCACTTCTCCTTCTCCAAACATATCGTATCATATCCTCTTCTTCTTCTTCTTGTCCTTGGGCTTCGAAACACGCCAAAACGATGAATGCAAGTCAAAT

>Rp-21

ACGCTTTTAAGTCTGAACCCAAGACCACATCTGAACCCAAAAAAGAAAAGTTCTTAAGAGGGATATAATTATTGATAGCTCATTTGAAAACCATCAACAGTCAACAATACTGTTCACTTTTCTGCTATTTCAACTTATAAGCACACACAAAGCTTGAAAGTCAGATTGATATATGCATATTACACCAGTTCAACACCAGACTTGAATTGTGACAAATAGACCAAAGGAAACTCAGCCACAGCAGGAACCGCAGGCAGGAAGTGAACTATGATCACGTCCCCTAATGCTATCTAGCCCACGAACATTGTCAAGGCCACCGCGAGGAGGGCCCCGTGGACCGCCACCATATGGTCCACCACCACCACCACCACCAGGACCTCCAGGAGCACCACCATCCCATTTCTTTCCACTTTTCCTGTAGGATTCTGACTTTTCCATCGAGAACATTGTTGCGAAAAATACGCCTATGAAGTTAACAATGGCCCAGAAAAAATCAGTGATTGTCTTAAGCCGCCATATTGATCGCTTTGATTTGACAACACCTCGCTCAACGTAAGCCATGGGAGACGAAGATTGCAGCAATCGAAGAATTGAAAGTGGAAATTGGTTATGGACTTCAATTCATT

>Rp-22

TTTATTTTGTTTTTATATAAATAGCAGCAATAACATTACAAAATCATCATAAGATAAACATTTACTTACTACATTGAGCAGTAAAAATAAAGCAACACCTTATGCATTCAATCCAATACCAAGTCTAGATATATACATCACATATATATTTATAATATATATATTGGAACAACACCAGAGTGAATTAATTAGCCTTCAAAGCTCAAACCTTGCCGCTAAGAATTCAATAATCTACTCTGTAATCACCGTAATCATGCCTCGCAGAAGCTGCGACGTCGTTTTCAACCCTCTCTGAAATCTTGTCCTCTTCATATTTGGATTGCTCTTCCAGAGCAAGCCCGCCCAGACCCCCAGCTACAGCACCCACAGCAAGCCCGGAGCCCAGGCCCATCTTCCCGCCCTTGGGCTTGGATTCATACGAGGAGGAATAATCTAGCGGCGCAGAAGGCCCGGTTGGAGGACGGTCAAAGAAGGGCCTCGGCGGTGGAGGAGGAGGAGCACCGGAATAGTAACCGGAGTAATAACCAGCAGAGTAGGAATAATCAGGGTAAGGTGAAGGAGGTGGAGAAGGTGAAGGTGAAGGAGCATATCCTCTATAGTAGAGGAAATGAGGGTTAGGGTTAGGGTTAGGGTTGGGGTTAGGGTAATCGAGAGTTGGGGGTGGAGGAGGAGGAGGACGACCTAGGAGACCAAGCTTGAGGTGGATCTTACCCTGAGGACGACCAGAGGGGCGCGTGAGGGTGAATTTTCGGAGGCGCGTGGAGTCATGGAGGTCGTTGAGGTCGTTAAGGGGGAGACGAAGGGTGGCGACGAGGGGTTTGGGAGTGTCGGAGGGTTTGGAATGGAAGATCTCGAGTGTGAGGATTGATTCATGGAGAGGCTGAGGGAGTGGAACGGCGAACCGTTCATTCCAGACGGGGGAGGTGGAGCCGGAATCGTCAGATTTGGTGGCGAGTCTCCGGTCCGGGTCGACCCAGAAGACAACGTAAGGCTTAAGGTCACCGTTTTTCCAGTTAACGTTTTTCAGGTGCTTGGCGGAGACGATGGTTAGGTCTAGATCCACCGACTTCGAAGGAGGGGGACGCCATGACGCCATTTTCTTCTTCTCTTACTAACACCAACTCAAGCACAGTTCGCAATTTTGTGTGATATATTGACAGATAGATATTTATCTAATCCCTTCCTTGACTTCAAGGAATGCCA

>Rp-23

CAACATAACTAGAAACAAACGAATACAATATTCAACAATAACAATAATATATTTTCATAACTAGATTACAAGACATAACTGGAGTCTTCACCTGGCCACGCAAGAAAAAAACCAGCAGTGACCCAAGGTAGAGAGATCTTGACAGAATCCTTATTTAAGATACACCAAATTATCCAAATCCTAAGGGTAGCCCTGCAATTGCCAAAAACTCCATTGCAGTAGCCCACCCACCTAAGACCTACAAATATACTACTACTAGATAATGCATGAGACCATTTTAACAAGTTACATATGACATGTGACCCGGACCATGACTAGGACTAGAACTAGCAATTACGGCTTCACAGAGAATCCCAATCGATCTCATAAGAGGGGTACTTGAGCAGATTAAAATTATCCGAATCACCTTCCCACTGTGGCTCACCAACATCTTCAAAGGTCAGATCCGAAAGGGGTGAAGAACCTTCAGATCCCTCGCTCTCAGTCATCACCGGAGACGTGGAAGCTTCAACCTTGCAAGAGTTCTCCAATCCCTTCTTCAGATCAACCTCATGCTCCTCCACCTTTGAGGCCATTTTTGACCTCTTGGATGATCTCGGAGGCTTCTCTGATTTCCCTTGTTTCTTCATATCAGCCAAGCCTTCACAAATAGCCTCAAGCTTAGCATCAACAGAGGAATGAAGAGGCTTGTACTCTCCAAAAGCACCCTCAACACATGAACCTTGGTGCTTCAGGTTCGGGAAGTTGAGTCTAGCCAAGTCACCTCTGAGCCTATATGCTGCTCTGTCATAAGCCAAAGCAGCTTCCTCTGCAGTGTCAAAGGTTCCAAGCCAAAGCCTTGTTCGGTTCTTTGGGAGTCTTATTTCCGCTACCCATTTTCCCCAATGCCGTTGCCTTACCCCTCTGTAGAGCTTGGTGGGTTTTGGAGGGGTACCCACTTGCTTCATGGGTATGGGCTTGGGCCCGAGGAAACTTTGGGTGGGATTGTTTTGCTGGGCCTGGAGCTGGATCTGGGCTTGGATCTGGTTAATTTGAGATTGGGTTAGGTTGTTGAGCCCAAGAACAGAACTTGGTTGCCCAAAGCCTATCAAGTTTTGGCTGTCTGAGAAGCCAGAGGGAAATAGGGAGGAGCAACCATCTGTGTAGAAATTGGGCTGTGTTGGAAGAAAAGGAGAGGGTAGGAAGTAAGAAGGGAAAGGAGAGTAAGAAGTAGAAGTAGAAGTAGAAGTTGGGTATGAATTTGAAGAAAGAGGTGAGGATGAAGAGGGAGTTGTGGAAGGACTTTTCATAAAAGGTTCTAGAACTTCCATTAACTCACCTCTGAAAGGATCTGACTGCATTTCTGGACTGTTGTAGAAATCCATTGTAGCTGCCATGTAAACTTCCAAGTACACACACACACACACACAGATCTTAGTTAAAATCAACCTGCGTCCTTAAGAAAAAATCAAAAACCAACTTAAGAAAAAAAAATACAAGTTTTGTTCTAGAAGAAAGGATGGAAGAGGTTTAAAATAGACAACCCCTTTAATCCAAGGAAAATTTGAAAAGAAAAAGCTTTGAGGGATAAAAAACAACCCTCTTGGCTGATTGAAAGATGAGAAGAAAGACCAAATTAACAACAACAAAAAAACAATTTTTTAACTTAATAAAAAAAAAACTGGATCTCGATTCTGAAAACACCTGGCTTTCTAGAAATCCTTTGAAGTTTGATTCTTGGTGAGACTAGAGACAAACTCAAAAAACCATAACTTGAAAGACAGATGATTAACAGAAAGGGATTCAGAGAAGCGGGGTGTTACAGAGGGGGGAAAAGGAAGATTGAAGATTAGAAGAACAATATGCAGAACCACAGAATTAGAACGCTCTCTCACTCTCTCTCAAGAGAAGTTTTGTTTGGTGATAATCTCTCAG

>Rp-24

CACAGATCGAAGCATCTAAAAACCAAATTCACCACCACCTTTCACTTTTTCAAATCTCTAAACTTTTTTCACTTCTAACAAACTCTCTTCTTCTTCTTCAATGGCGGATTCCGCCTCTTCTCCCGCAACCCTAGAACCCAACCCCAAACCCTCCGAATCCGGTGCTCCGAATTCCCACCCCAACCAACCCGATCCAATTCCCCAATCAACCCAATTGCAATCCTCAACACCTTCTTCTACACCGAACCCTAATTCCAATCCACCGTTAGCGTCTACACTGTCGCTGCCTCCACCGCCGTCTCTACTCTATGCGCCGCCGCAAGTCCCCGGCGTCCTCCCTCCGTCGACGCCGTCGTTCCGGCCGCTAGCTCCTCAAGTGCCGCAGTTCTCTCCTTTACCCAATCCCGCCGGTGCATACCAAAACCCTACCGTTCCTCCTCCCGGCGTGGCCAGCGCCGCCGTGCCGCAGATGCAACCGATGATGTCGTATCAGGTTCCCACCGTTCCGGCGACTAACCCTGCCTTGCGACCCTTTGCACCGATCCCAAATGGTTACGCCGCGCCTCCACAAGGAACCGTTACTCCTGCAGTGTCTTGATTGAATTCCGTGAGAAAGGTTGGAAGGCTTCATATTGCTTAAAGACACCCCCCTTCTCCAACTCATTTTTTGGTATGCACATATTTGCCTGGTTTATTAGCTTGTCTTTTACCGAAATTGGAAACCAAGCTTACACAGAGTGATATTGATAATGGAAATAATTGAATAAATAAATAAATAAAAGGATTGTTCTTTTGTCGATCTAAATAATGTATGGTTGTGTCATGCTCATTTTAGTAGGCCACTTTCTGCTAGTTATAAGGGTACTTTCCCTTCCATGACTTTATGAATTCCAGAAGCCGTCCATGTTTGCAACTGAATGCACGTTGGCCTTGTGTCATGAGGTATTGCAATCTTTTCTCTCTATTGCAGTTTCATTCCATTTTTTATGCTGCAACATGGGCTCCTATATATAAAAAAAAGAACTTATAATTCAACTAGAATTCTTCTTAGTAAACTATGCATGCATGAGATTATGAGAGTTACTATTTGCAAATGGTGGATGGGATAGTTTGAGAAGCCTGCTATGTTGCAAGTGATCAGACAAAGGTAAGCACTTTCTACACATCCATGAATTTCAAACTGGATAGTACATCTTATTGTTTTCTTCAGGTTGTCATTTCAGCTGGTGATTGTTGGCTGTGCTGCCATCTCTGTTATGCTTTTGATGTGTGGTAACAGCATTCCATTGTTCTTGTTGCATTTTTTCTTCCGCTATCTTTAGTGTTGTATTGATTAGTTTACAATGTATATTCACTCACGATCTGTTCTAATATATTTGCATAGATGTTTTTTCTACTCTGTACAGGCATTCCTCGTTACCCCCCTCCGTATGGAACTATGGTTCGTCCTGTATATCCTCCACGCCCTCCCGGATTAGTTAATGTACTTCCAGTATCACGTCCACCTGTTGCAGGGATCCCTCCAGTTCGCCCTATTATTCCTCCTGTTGTCAGACCTGTGGTTGCTCCTAGTGTTACTCAAGCTGAGAAGCCACAAACCACAGTTTACATTGGCAAGATTGCACCGACTGTGGAAAATGAGTTCATGCTTTCTCTCCTTCAATTATGTGGAAATATCAAGAGCTGGAAACGTCCTCAAGATTTATCAAGTGGAACTCCTAAAAGTTTTGGGTTTTATGAGTTTGAGACTGCTGAAGGGGTTCTCCGTGCTATGCGTCTCCTTACTAAATTGAATATTGATGGGCAAGAACTAATGATCAACATCAATGAAGCTATGAAAGAATATTTGGAGCGGTATGTTCAAAGGAAAACTGAGAACTCAGAGAAAAAGGAATCTCAGGCAGCAGGAGTTGAAAAAGATGATGAAGGTGCAAAATCTTCTGATGCAAATGAGGTTGCAAAGCCCGATGGAGACCTCTTGAATAAGGAGGATAATGATTCAGGGAACAAGAAATCCCATGATGTGGCAAACTTTGGGATTGTCACAGATGAAGATAAGGAAGCTGATCGAGATGCTTTAGAAAAGATCACTAAGATGATAGAGGAGAGGTTGAAAACAAGACCTTTGCCTCCACCACCTGCACAGCCAACTGGCAATGGTTCTGTGAATTTAACTTCAGAACAACCTGCTAAAACAAGAGATGGAGACTCTGATGTGGATACAAAGAAGAGTGAAACAGCTGAGGATAAAAGTGAGAAAGAGACAAACAGTGATAACAAACCAACCAGTGAACATGATAAACCCGAAACCCCTGATAGAAGGCATGATAGGAAAAGCAGAGAGAGGGACCGAGATAGGGAACTAAAACGAGAAAAGGAAAGAGAACTTGAAAGATATGAGAGAGAAGCTGAACGGGAACGTATTCGAAAAGAGAGGGAACAAAGACGGAGGGTTGAGGAGGCTGAGCGTCAGTATGAATCATATTTGAAGGAATGGGAGTATAGAGAAAGAGAAAAAGAGAAAGAACGCCAGTATGAAAAGGAGAAGGAGAAGGAAAGGGAACGCAAACGGAGAAAGGAGATACTTTATGATGAAGAGGATGATGATGAGGATTCTAGGAAGAGGTGGCGGAGAAATGTGCTAGAGGAGAAGAGAAAGAAGAGGCTGCGTGAAAAGGAAGATGACCTGGCTGACAGACAAAAAGAAGAGGAAGAAATTGTTGAGGCTGAGAAGAGGGCCGAGGAGGATCAACAGCAGAAGCGGCAGAGAGATGCGTTGAAGCTATTATCAGAGCATGTGGTAAAGAGCAGTGACAAAACTATGTCTACTGAAGAGATTACTAATAAAGTAAAAAACATTGTTGCTGAACAAGATACTGTAGCTGATTATAGTCGTGAAGGTCACATTGGTGATGATAATTCACTAAATGCTATCAACGATGAATCAGCCATGGCATCTGTGGCCACAACTGATACACAGTCAACAGGAAATGCTCCTACGAAGAAGTTAGGATTTGGTCTAGTGGGCTCAGGGAAAAGAACAACTGTCCCTTCTGTTTTCCATGAAGAGGAGGATGATGATGCACACAAGGATAAAAAAATGAGGCCTTTGGTTCCAATTGATTACTCGACAGAGGAATTGCAAGCTGTTCAACCTACTGCTTCTGGGGCAACACCACCAAATTTGGCTGCAGCTGCAGAATTTGCAAAACGTATATCCAGTACCAATTTCAAGGAAGAGAAGCTGGACGGAGATCGGGATAGAAGTAGGCGTTCAAATGAGAAGTCTAACCACCGGGACAGGGACAGGAGTGATGAAGATGGCACTCACAACAGAGATGAAAACAAGGAGAAAATTCCTGACCGCGACAGGGATCGAGATCATGGCTCGGAGAAACTTAAGACTCATGATAACAGGAGGCTTTTTGATGCAAAACAGTTAATTGATATGATACCAAAGACCAAGGAGGAGTTGTTCTCATATGAGATTGACTGGACAGTGTATGACAAGCATCAATTACATGAAAGAATGAGACCATGGATTTCAAAGAAAATCAAAGAGTTTTTGGGGGAAGAAGAAAATACCTTGATAGATTATATTGTTTCAAGTACACAAGAACATGTGAAAGCATCCCAAATGCTAGAGCGACTTCAGACCATATTGGATGAAGAGGCTGAAATGTTTGTTCTCAAGATGTGGAGAATGCTCATCTTTGAAATAAAGAAGGTAGAGACAGGGCTTGCTTTGAGGTCAAAATCATGATTTGCTGATTATTGTGTTCCACCTTTTCTTTTCTACTGATTGATGTTTTTACTACCATCCCTTGGTATGTCTTCGCCCAAAATTGTAATATGGCTGCATAATCATTAACGCTTTTCAATCTTTATTGCTTCTAGATTCAAGTAGTTATCTGGATAAACCCCCGTTTTCTTTATCCATGAGAATCCGGTGGCAATTTTGTCATGTGAATAGTTAGAACTTTGGATTTGTTGATGTCACTTCT

>Rp-25

GATATTTACTTATTACAATGAATATAAATGATACCTTTTAGATTTTACGCATAAGCGCATTCGTACATCAACATTTTATAAACGTAAAGATAACATTCTTATTTCGGCAACAAGTTGAGAAGAACAAAATATTCAAAAGAAAAGAAAGAAAGAAAGAAAGAATACATAGTAACAAATGAGAATGCATTCAAATTTATTAAGCTACTTCATGATTCCTCAGTATCATAGGTTGGTTTGTGAGCCTACATTATAGTATGAATAAAATTGAAGCCTATATGTATATTGTATACGTATACGTGGGTTATTCAGCACTTTAGTGGATTGTTATAAATCATGACCATGTCCCATCATCGCAAGGGGAACAACCCTCTTGTGCAATGTCTGTTATCTGAACACCAGAGTCATATATCTCCCCAGTTCTCCAATCAGCAGGCAGAACTTTCTTTGCCCAAATCCATTTACCATCATACCCTGCTGTTACCACTATCCTGAATTGCAATGGACCTTGTGGTACCCTACTTGTGTCCCACACAGCCCCATGATTCCTAGTCATGAAACCCCAGTTTGAAGATCCAACCTGAGCTACATCAACGGCTACTATCTCTGTTTGACCCCCTTGGTACAAAAATTTAATTGCTAAATAATCTGGTTTTTTGCTTGATTCTTCTACTCGAACAGCCAAATTTTTATTTTTGTGCTCACAAGGTACTCTGCTCCATTAAACCCATGAAGGACGAATCAGTAATCAGCAACATGTTTATGCATAACCATTTTAAACTTGACATTATCTAATTAGTTCCACTGAGCTAAAACCATAATAAAATTAATATTGCCTTCTGATCTGATGCTTGTACACATGCAAGGACATAAAATATGTGACATTATACATTACAATATTATATTAGTATATACATTAGACCGTTACTGTTATTAATGCGCTATGATTATCTTAGGGATTCATAATTATTTAAATGTTGGACCACATTGGTGTTCTCTTTTTTGGAGGTGGGCCGGTGCCACTGCCCGGTAGGTGGGAAGTGGGGACAGACCAAAACCGATGGCTTAGCAATAAAGCAGAAAAAAAAACAACAAATGCTAACAAATGGAAAAGGATAAAATTTATCTAGAAACAGCAACATAAGAACTAAACTGCAGCTACAAAATGTGATTTAAGCCTACCTCTTGTATTCAATGTCAACAATGCCAAGCTTCAAAATTTGTTGGCCCATACCCTTCTGAGCCATGGCCATGAAAGCTCTGCTGCTGAGGACAAAATCGGTTTCATTGTTGTGATTAAGATCAGTCAATAGGACTCTGGTACCTGCTTTTGTGCAAAGAGTTGGGTTCTTGCATCTTATCTGAAAGCAAGCACCACAACCAGCTCCATTTTTGAAGAGGGAAGGCACACCAGCTGCAAGATGTCCACCACTCAAGTCCAGTGCCAAAGAGCCATACCCACATGCCCCAGATGAAAGAGCAGAAGCTTTGGAGAAATAGGAAGCTTTGGTTTGGTGGACACAGCGATCACAAGCAGATGCAGATGAAGCAAGAAGGAAGATGAAGAAGCATAAAAGGAAAGCCATGTATGTTAATATTGTTTGGTTTGAG

>Rp-26

ATAGAGCTAGGGTAGGGTGAGCGTGATTGATCCCAAATCTGAAAACCCTAGTTTCGAACTTCACTTCACTTCAAGAATCAACCAAGAAGTCATGGGGAGGAAGCGAAAGATCGCACCTAAATCTTCTCAATCTGCAGAACCCCCTCTGAAACAGCATCAACCAGAACAAGATAATGCAGCAGAAGAATACGAAGAGGTCGAAGAAGAAGTAGAGGAAGAAGTAGAAGAAGAAGTAGAGGAAGAAGAAGAAGAAGAAGAAGGAAGGATGTGGCACGAGGCGAGGAGGTCGGAGAAGAAGGTGCACGATATGATGGACGCTGCTCGAAAAAGAGCACAGAGGCGAGCCGTTTACCTTGCGAAGAGGCGCGGCGATCCTCAGCAATCCATTCAGGTCGTTGGATTTCGTTCACGAACCTATCGAGACGATGCACTTTATCAAGCCACACAGGATCAGCAGGGCCTGATACCTTGGAATGGGAAACAGGATATTTTGATTGACAGATTCGATGGACGTGCTCTCCTTGATTTCATTCGGGATACTAGTCATAGGCATGTCCAGAAAAAGTCTGAAGAAGAAGAAGAATTAGAAGAGTTTGTTAATTTTGAGCGTTATCGGGATTTAATAAAGCATCGGCGTAGAGGATTTACCGATGAGGAGGCTTTGCAACATGTAAATCAAGAGATGGAGGCCAAGGCTGCTGCTCCATTTACATCAGAGAGATCTAATTTGTCGCAGCCTGCTGCAAGCAAAGGATCTTACTCACAAGTCGGGTTTTCTTATGAGGGGAATGGAAAAGAAGAATCCCAGATTTCAGATGATGATGATAATGATAATGATAATGATAATGAGGAATATGATGATGACGAGGATGATGAGGATTTTAACAGTGATGATAGCAATGATGAAGGAATGGAGTTAATTGCAAAAGAATATGGGGTTAAAAGGTATGGTTGGCTTGTTTACATGGATAAGAAAGCTAAGGAGGAAGAAAAAAGGCAAAAAGAGATGATCAAAGGCGATCCTGCAATTAGGAAGCTGAGTCGTAAGGAAAGAAGGAAAGCTTCTCAGATTGAAAGGGAGAGAGAGAGAGAAGCTACACGGATATCTGGAACTCGCGTGCTGCATCATGACCCCTACCGGGAATCAAGGCAGAGTCCTACTTATGAAGCTTATTCTCGTTCTAGAAGGTCAAGGTCCAGATCACGGTCCTACTCTCCTTCGTACTCAAGGCGCTATTCACGGAGCAGTCATCCTGATGATATCCACCGAAGCAAACCAAGGACTCCTAAAATAGAATACATCACTGAATTTGGGGGCTCTGGAGAAGCAGCTGAACCTAGGCGTGAAGGATTTTCTCCACCACAATCTCCTCCATCTCAAGTTGATATGTTAAACCGGCCAGCTTCTGGTTGCATACTTGAGGCATTGCATGTTGATCCTGCTTCTGGTGTATCTGTTGATAAGGGCACTAAAGTCTTGAAGCCGTCAGTAAGCACATCTTCAGCATTAGCAAAATTAAAGGCTAGTGGTTCAGGAGGGCCCTTAAAAAATCAGCAAGGGGAGAAGAAAGAAACTCCTCAAGAAAGACTTAAAAGGATCATGAACAGGCAGCTAAACAAACAAATTAAAAAGGATACTGCTGCAGAAATAGCCAAGAAAAGAGAACAGGAGCGGCAGAGGCAGGAAAAGCTTGCTGAAACAAGTCGATTGAGTCGGTATAGACGCCGCAGCCGCAGTAGGAGTTACAGCCGTTCCCCTCCAAGAAGGTACAGGCGTAGTAGAAGTCCAAGTAGAAGCAGGGGTTCCAGAAGATATTATTCTAGTTCTCGCTCTCGTTCTCGTTCTCGGTCCCCCTCTCGCACCCGTTCCAGGTCCAGGTCTAGATCTCCATATTCTCGTTCTCCCAGGATAAGAAACCGTTCGAGGCACTGATGGAGTGCTTGTATGCATGCAGAAAGGTTGATGAGGTTTGAGTGGAATTCGTTTATTTATGGATTTTACTGCTCCCAAGTGGTTTGAGGGAAACTCTTACCACCGATGTGCTTAGTTGCTTTGTCTTGAAGTCGCGGTAGGCAATCTTTCTGAACATGTTTCTTTAGCATTTGCTGATGTATATTTAACATGTTGATGATTGTGAGTAATTTGATGTCTCGGAAGTCATATCAG

>Rp-27

ATTTTTCTTCACATGTTTGTTTTTCTATTTTGATAAAACAAAGTTTTTGGATGATCAGAATCAACATGGCTCAAATTCCTTTTTGATTTTTGTTATATAGTGTATGAGTTTTACAAAGATTCAATCTGAATTGATCAATATAATCAAACAGCAGCTGGACATCCACCCTTTCTAGGGTCACTTACGGCTGTTAGAGTACCATACTTTGCCTGTGAATTGGTGTCTTCACCAGTTTTTCTATTCATGATAATTGGTGTTTTGAAAGTTTGGACAATAAGCTGAGTGACAGCTTGTGCTGGACATGCACTCAGTTGATGACCTCTCTCTTCCAGGAAATGCCTCCTTTCTTCTGAAAGTTCAATGTGATCACCATCATGATCGGTCAACTTCTCGTAGCGAACTACATTCGGTATTAGCTACATGAGATAAAGATCCAAGTTAGATAGTAGGTAGGTGCAAATATTTGAATAGAGAGTAGTTAGAACCTTGTGAAAGACCCTTGGACTCAGAACTGCATCCAAAGGTTTCAATCCCAAGATGAAATGATTAAGAAAGACTTGAGTCACTGCCGGAATAATGTTCATTCCACCGCTGCCTCCAAGTACCCCGACCAACTGATCATCCTTTGTGATGATAAGAGGAGTCATGGAAGACAATGGTCTTTTGTTTGGTTCAATAAAATTTGTTGGAGCTGGAGGTAGTTTATCTGGGGATATATCAGTGGGTGTAGAGAAGTCATCCATCTCATTGTTGATCACAATACCGGTTGAAGTAGAAAGAACCCCAGCTCCAAAAACATAGTTTACGGTTGTCGTTAGTGATACAGCATTTCTATCAGAATCCACAATGCACAAATGGCTTGTTCCGTGATCTCTAAGTTGGCTCCACCTGTTCATATAGTACTCAGGAGGGAAAGTGGTGTTATCAAGTATCTTGTGCTGAATATTTTTGGCAAATGATGGGGAAAGCATATCTGATACAGTACCTCGAATGTTTTCAAAGTCGGGGTCACCCAAGTTCATTCGAACTGCAAACATGTGTTTCAGAGCTTCAATTAGTCGATGTAAACCCAGATTTCCCTTTGCAGCATCAGGAACTCCGTAGCTGTCAAAGATGTTGAGGACCAGAGAAAGAGCAAGAGTTCCACTTGAAGGAGGTGGCATTCCATATATGGTGTATCCCATCGCTTTCACAGTCATTGCATCAGTTATTTCCACCTTGTAATTGCGTAAATCCTCCATTGTTAAAATCCCACCAGCTTCTCTGACATCCTTTACTAACTTTTCACCAATGGTTCCATTGTGGAAAGCTTGTGGCCCTTGCTCTGCCACTACCTCTAAGCTGTAACCAAGTTCCGCATTACGACATATCTCCCCTTCTTTTAACAGAATTCCATTAGGTGCATATATATTTTTTAACCCAGGATCATTCATCATCTTATTCGCATTTTTAGCCATGTAACCTGCAAGAGTAGGGGACACTGCAAAACCATTTTTAGCAAGTTCTATAGCTGGTTGGAATAAGGTCTTCCATGGCAATCGTCCATGCTTCAACCAGGCTGCATGAAGGCCAGCTATCTCACCAGGAACTCCCATTGACAAGGCACCTAAGGTCTTGGCTTTGGGATTGTTCTGATACATATCCTGTGAAGCAGCTAAAGGAGCAGTTTCTCTGAAGTCAAAAGCTTGAGTTTGGGAGGTTGAAGAAGATCGGACGACCATGAAAGCTCCACCTCCTATGCCACTTGATGCTTGAAAAACAACACCGAGGCATAATGCAGTGGCCACTGCAGCATCAACAGCATGCCCACCCAGCCTAAGCATTGAAACACCAACTTCAGAACACCGGGCGTCATCAGCTGCAACAACTCCTAGTTCTGATTCAATAATATCTCCCTGATTTACTGTCATTCCCTCATTATATTTTTCTGCCCCTTGTAGTACCCCAAAACTTACATTGCCTCTGACTGCTAGCCCCACAAAGGTGACAGCAACGAAAGCGAGAAGAAAGCAGAGAAACATCCTCGTTTTGTGTTTCTTGGACAAAACATCATCGTGTGCTGCTAACAAAGGAGAATCCATGATGGCAGCACCTGAGGAGAGGGCGAGGAGGAGGGAGATACTGACGATGGAGCAGTGGGTGAGTTTGAGTTTGAGTTTGAGTTTGAGGGGCAGAAAGAAGAGAGAGAAACAAGAGTTGTTGGTTGTAGCTGACGCTAAGGTGGCGATGGTTGCATA

>Rp-28

CTTTTCCCTCATAGCTGTTGCTGGTGTGCTGCAATAAAAAGGGCTACCCGCTCCATCCATTACTCCCTCATCTTCTCTCGATCGAGGTCTTCTCGATTTTTTTCTCTACTCTACTCTTTG

TTGCAACAAAAAAGCTCATTCCTTTAACACTTCATCTCTACTAGTCAAGAGGATTTGGAGCATGTGCAGTGGTTCAAAAAGTAAACTTTCTTCCTCAGACCACATCATGGAGAATAAGCTTCAGAAGGAGAATTACGATGGCCTGTATAACTCATCAATTTTGCTTGAATTGTCAGCATCTGATGATGTTGAAGCTTTCAAAAGAGAAGTAGATGAGAAGGGTTTGGATGTCAACGAGGCAGGCTTTTGGTATGGTAGAAGAATTGGATCAAAGAAGATGGGATCCGAAAAGAGGACCCCTCTCATGATTGCTTCTCTGTTTGGAAGCACTAGGGTGCTCAAGTATATTATTGAGACAGGAAGGGTTGATGTGAACAAGGCCTGTGGTTCTGATAAGGCCGCTGCTCTTCATTGTGCTGTTGCTGGGGGCTCTGAATTCTCACTTGAGATGGTCAAGCTATTGCTTGATGCTGGTACAGATGCTGAGTGCCTTGATGCTAGTGGAAATAAGCCGGTTAATCTGATCGCTCCTGCCTTCAATTCTTCATCCAAATCACGAAGGAAGGCCATGGAGTTATTTCTCAGAGGTGGGGAAAGAGATCAACTCATCCACCAGGAGATGGAGATGTTCTCTGGTCCTCTCTCATCAAAAGGTGGAGAGAAAAAAGAAAAGGAAGGAAGTGATAAGAAGGAATATCCTGTTGATATCTCGTTGCCTGACATAAACAACGGTGTATATGGAACTGATGAGTTTAGGATGTTTAGTTTCAAGGTGAAGCCATGCTCAAGGGCTTACTCCCATGACTGGACCGAGTGTCCATTTGTTCATCCAGGGGAGAATGCAAGGAGGAGAGACCCAAGGAAATACCCTTATAGCTGTGTTCCCTGCCCAGAGTTTCGCAAGGGGGCCTGCCAAAAGGGGGATTCTTGTGAGTATGCACATGGTGTTTTTGAGTCATGGCTACATCCTGCACAGTACCGAACAAGGCTTTGCAAGGATGAGACTGGCTGCTCTAGAAAAGTTTGCTTCTTTGCTCACAAACCTGAAGAGCTCCGCCCGGTTTATGCTTCCACTGGGTCAGCCATGCCTTCACCAAAATCTTATTCAGCTAGTGGACTTGACATGACAGCAATGAGTCCATTGGCTCTTAGCTCCTCATCTTTACCTATGCCTACTGTTTCAACACCACCCATGTCTCCCTTGGCAGCTTCATCATCTCCAAAGAGTGGAAGCTTGTGGCAGAACAAAAATAACCTCACTCCACCATCTTTGCAGCTCTCGGGTAGCCGGTTGAAGACTGCTTTGAGTGCCAGGGATTTTGATCGGGAGATGGAACTTCTTGGTCTAGAAAGTCCTGCTCGTCAACAGCAGCAGCAGCAGCAGCAGCACCAACAATTGATAGAAGAGATTGCCAGGATCTCTTCCCCATCCTTCCGGAGCAACGAATTCAGTAGGTTTGGTGATATAAATCCAACTAACCTTGATGACCTTCTAGGATCTGCTGATCCTTCTGTGTTATCTCAACTACATGGACTTTCTATGCAACCTTCAACACCTACACAGTTGCAATCTCAGATGCGCCAAAACATGAACCACCTCAGGGCAAGTTATCCATCCAATATCCCTTCCTCTCCTGTGAGGAAACCTTCACCAGTTGGGTTTGATTCATCAGCTGCTGTGGCAGCTGCAGTGATGAATTCCAGGTCTGCTGCCTTTGCAAAGCGAAGCCACAGTTTCATTGATCGCAGTGCTGCAACCCACCATCTTGGGCTGTCTTTACCTTCCAACCCTTCCTGTAGGGTGTCCTCTGCCCTTTCAGATTGGAGTTCCCCCACTGGGAAACTGGATTGGGGTGTAAATGGAGATGAGCTGAACAAGCTGAGGAAATCCGCTTCTTTTGGGTTCAGAAACAATGGGGTTGCTGCAACTGCATCCCCTATGGCACAATCAGAACATGTTGAGCCTGATGTCTCTTGGGTTCATTCATTAGTTAAAGATGCACCCTCTGAGAGTTCTGAGATGTTTGGTGCTGAGAAGCAGCATCAGCAACAGTATGATCTCAGCAAAGAGATACTTCCACCATGGGTGGAGCAGCTGTATATAGAGCAGGAGCAGATGGTAGCATGAGTAAAGTGCTACCATAGCAAAGCTTCCTGGTTATTCTGACAAAGTTTATTTATTTATTTATTTATTTTATTTGAACTGTTACTACTATCCAACACTACATAAGAGTATAGTTAGTGATAGGATGAGAGATGTTGCAAGGAACAAAAGTTCAGGTTAATCCAGAGATTTGAAGAGTTTTTCAGCTTCTAGATCAGGAAAGACTTGGAGAAACAATGGAGATGAAGCAAGGATGCTGAATTTTTTTATTGTATTATATAATTTATTCCCCCATTTCTCAAGTGGGTCGTTGTACTTTATTTTAGCAAGTGGACTAAGTATTGTGTGTCATTCATTGTAACAACAATCAACACCAATTAAGTTTTTTTCTCATTGGCTA

>Rp-29

CTTATAATAATGGAAAGAATATAATAATAAGACGACAACTGATGATTTCCATACACAAAACCCAACAATAAATAATCTGCCTGGTTTTTATACCGTCCAAAGAAAACAAAGGAAGATCGTCCAAGCTTGATTGATACACCATTGCAAGACCACACAGGGCCCATCAATCGCCAGTAACCAGGTGAGCACAATAAAGTGCTATCAATCCCTTCCTTCCATACTCGTGAATCAGGAAGACCAGTATTTGTTGTAGCCAGCAAAAATCGCAATCTAACACTCAACCTGCCCTACACTCAAGTAAAAGCTAATCAAAATTTTTATTTCTGCCAGACCAAAAATTATTAATAATAAAGCCTTTCCAAGTTTCTTCAATTCCAGCAGATATTTCTTGCCCCTTATGACACTGGTGATGCCCCACTAATAGTCTGTTTAGCCACATAAATTTGCTTGACCTTTGATGAGGAAAAATTCTTATCTGCACCCAGAGATTGGTTTCTTCCTTGATACCCCATTCTGCGATGGGACCATTCGCCCCCAGAACTATTTCTCTGAGAAACACCACCTCCTCTCTGATAATTATTAGTAAGTGCCGAGGAATGGTCATACTGCTGAGTAGAAACATTAATTTGTTGTGAGGGCTGACTCTTAAAAGCCGAACTTGCATTCTGGCTATTACTGTTGCTGCCATTGCCATTCTGAACATCAACCTTGGCAGCATCTGTCATGATTATCACAGATGGAGTCTTGTTGACAACACTTTGTGTTGAAGTCTTGGCAGCAGTGGAGTTTGAAGTGTCCACTAATCCAAGTTCATCAGGCAATTGGTTAACATTTACATCAGCTGCTCTTGGAAAATTCCTATCACCATCAGAAGATGTCGAAGCTCGGGAACCGGGGAACCTTTTGACATTTAATGGCTGGTCAACAGAAGGGCCATGGTTCAACTGAGAAGTCTGTACCCCTTCTTGTTGATGCAATGACATTGATAGAGGAACGGACGAAAGTGGCGCATTAGGAACATGTGGCCATCGAGCTTGTACTGACATCTCAGTGGAGGGCTGAAAAGGAGAAACATCAAACATAGCCACAGGAGAAGCCATTGGCAGAAGTGGCGAACCAGGAGCAAGATGTTGAATTTGAGAAGGCATGTTAGCAGGATTCCTCTGTGAAGATACCATATTCATATTGTTCATATCCCCTTCACTGGCCCCCACGGCAGAAGATGTAGGGATGTGTTTCCAATCAGGCTGCTTTCCAGAGGGGATATAAGTGGTGCCCATGAAACTCAAGCCAACTTGACCAAATTGTCCAACAGGTGCAAAATGGTTATAAACAACCATGTGTGGTGGCCCCTGGACTCCTGGAATGCCTCCAGGGGGAGCAATAAAAGGTCCAGTGAATCCTGTTGGAGGACCATAAAAAGATTCCACACCAGAATGGCACTGTTGCCAGTTCCCAATTGGCCTTGAGGCTGATGCAGTACTCTTTTGAGACTGTGACTGTGTAGTAGATGCAGACTCATCATGTGGTCCAAAAGCAAACACAGGTCCTCCCATCATAGGATTCATTTCATAGAAAGGAAAATGAGGGGGAGGACCGGAAGTAAAATGAGGAGGAACAGAAGGAAAATGCGAGATCATTTGGCCTGAAGAATTTTGTGTATTTGGTAAGGGTGGCCACAAGGAAATTGGTGGAGTCTCAACAGATAAGTCTGCTGGAAGAGATACACTTAGAGGCTCTTCAGATCTAGATTGACTTGCTGATGGCTGCTCACAACCTCCTGCTACTGCCCTGTCAATATCTGTAGCTACAAAACTTTTAGCATCTGAAACTGATACAGAACAGGTACCCAATCCATTTCCAACAATCTCATCACTACCAATGGCAGCAACAGCAACAGCTGAAGCAGCTGCTTCGGCTTCGGCTTCAGCATCACAATCTGCTAAATGGCCATGACATTCATTACCATGTTTTTCTTTATTAAAGAAAAGGCTGCAGTTATTATCAGATCCACCAAGGTTGTGAGATATTTGCATGTCTGATCTAGATGATCGAGGGGGGCCAATGCCATGTGACACAGCACGGCTGCTGGGAGGAAGAACAGTTGGAGATGTTACTGCCCCAAATTGAATTTTCTCTCCAGCAAGCAAGGAATTAATTGGGCTGGCAGCAGACGAGAATGCTTTCTCCTTTGTCAAAATGGATGATGTTGGCAAGCTGGGTTCATTAACAGCAGTTGTCATATTGCCTACAGAAGCCTGTGAATCAAACTGTTGAGGCTTCATAGCCTCATCAAGTTGTGTCTGTGTAAGCGCCATGACCTGTTGACTAATTTGCGCATTGCCCCAAGAGCCCAAAGATGTCTGAACATTATCAAGAACCTTGTTCTTGCTTTCAAATATCACACCAGACCCAGGGTTGTTTTCACCACCAGAAACTGCTGGGAGACTCGAATGAAGTGACCTGTTTGCCTGTGATCTTAAATCAGGCTGGGCATCAATTTTCAAAGGAGGCGTGCCTATAGGAGGTAATCCAAGGTAGAATAAGTTTGAATTAAATCCAGATGAGGCATCAATCTTTGCCATTCCACGTGCCTCAGCAGCAACAAAATCAGTACAAATACTGTTTGCCACTTCTCCAGAAGAAATGGATCCTTTGTTGGTCACGTTACTTCGTGAAGTGGAACGGGGTTTCCGTGGCACCTTTGTAACCCGAGACTTGGCCTTGATTTCTTTCTCTCTTTGTTCTCGCCGATCATTTAGCATTTGCCTCTTTGACCTGACTTCTATGAAGTCATCTTCATCACTAGGAGCTTCAATTCCAGGTTGCTCAAACACACGTATAATTCCACTCTGCAATGGAGCATCAACATCATCATCAGAACACAAGTTCCTTTTTAAATTACTATGTCCAGAATGTGAGATGCTCTGAGTCTTTATTGATTCTTTTCCATCAACCTTCTCAGCTCTGCTTCCAGAATCCACTCCATGTGAATTTTCTCCAACTGATTCTACTGTCCGTTTTCCCAATTTATTGGTGAAAGCCTTCCTAGGTCCAGTTCTTCCAGATATGCCTGTTCCCCTTCCAGTTATATTTGACTTATTATCCAACCCAACCTGATCAATCAAAACAGAACTAGAAGACTGCCTCTTCTCAGCACTTTCCCTAACTCGAAACTCAGTGCGCTGCATATTCCGATTAGGCCTCCTCACAAATCCTCTGGAGTCTGTGCGATTGACCCTTGAAGCTGGACCTGATGGTTTTGAGCTTGAAGTTTTTACTGTATAAACATATCTCTTTCCTCTACCACCAGATACAGGAGACTGTGCTTTTGACTCCATAAAATCTTTCTCTCTTGAAACTGAATGAAGTGATGCATCTTTAGCAGGAGGCTGAACTTCTGATTCTTTAACATTGGATGAAGTGTTGCTGCTCTTTCCAACTACATTTTGGCTCCCCTGCTTGTCCACTTGGAAACATGTAGCGATCCTCGTGCTATTATTAGTATCATGAGGGGCCTCAATTTGACCCTGCTTTATTCCTGCCAGATTTTCGGCATCCTCACTTGGCAGTGAACCTTGTGATAAGTTTCTTGAATTACCTGGTTGGCTGTCAACAGACTGATGCCTGATCTCTTCTGGACCACTTTGAACTGGCGTTTGACCTCCAGGGTTATGATTAAAAGAGAAACCAGATGGTATATTAGGCTGAACAAATGACATTGATTGAGGACCCAGAGGCATTATCCCCTGTGATATGGGAGATGTATACCTTAGCTGGCCAAATTGAAACAAAGGAGGCTGTGACGGGTGCATGTGAGAGAGGGGTGCGCCAACCTGTGGATGCAGATGCAGTGGCATCTGTATAGAACCAATCTGTATGGCTGGTACTGGTGATGGTATCAAAGATGGACCAGAAAAAAGACCAAACTGAAGCTTAATGGGTAACTCAGTTTGACTTGGAGCAGCAGCTACATTAGACGTAACAGTTTGACCTGAAGATGCGTAATGAGGAGCAATGGTAACCGAAGTTGGGGTACTATAATGGGTAGACAGGCCATTAGAAGCTTCCACATTACCAAAACTCTCAGATGCCTCAGACGAATGAGCATTACTAGGCTGAATGACCAAATCTGGAGTTGGCTTCTCTGATTCCTGGAACACACTAGAAGAATTATTAAGATTCACCTGAGAGGTATCATCAACAGGTTGGAGGGCTTGGCCATTATTGTGTGCATTGTCAAAAGGTACATGTTCTTCTAGAGTGATGCCAGGAGCCTGTTGTGCCATAAATGTAGTTTCTTCATTTTTCAAAGTCCTTTCAAACTCATCATTGGGCATCCCAACCTGAACACCCTCATCAAATCCTAACACTAGATTATCCATCAAGTGGGGCAATCCTTTCTCCTGCAAATGCATATCTTCAAAATCCTGGTGGAGGTCAGCATTATCATCTCCTTCATGCACTTCATCTTCTTCTCGATAATCTTCATCTTCATCATATTCTTCTTGTTCCTGGAACTGCTCATTATTCTCAGTAGTCCATTCATCATCATCGCCACTGGAGACAGCACATGAAATAACTGCATTCTCATTTCCCATTGTGGCAGGTGTTGCAATGGATTCATTATCTGGAGCTGAGAGGGGGCCATTTTTGCTTTCTTCAGAAGCCAATATCACAGGAGAATCTCCAGAGTCATCTAAGTCATCATGAGAGAGATGTGTTGGAGAACTAGGGGGGCTTGAAACAGAGAGTGAAGACTGAGAATCACACCTCGGTGTTGTCTCCACTTTATGGTCCTCATTCTCGGTAGTCTCTTGCAGGGCATCCACCATTTCAGGTTGTCCACGATTCCCATTATCATAACTAGTTGGCCGGGTAGAGTCACTCCTTGTTGCCTGATTATATGGAATCTCATTTTCTAGGAAAGCAGAAGGACCAGGGTGTCCATTTGCATTCCTGTAAGTTCTGTGAGCAGAACCTAGTGAAGGAGGGGGAAGAACTCGTGGCTGCCTAACAGAATACCGTGACCTCCCCAAGGCATAAGGTCCATCTGATTCAGAATGTGGATAAGTACGTTCAGGGAAGGGAGGAAATGCATTAACACGGGGACGGCTCTGTGTCCAACCATCGCCAAATCTTTCAACAAAATTCTCATGAAAATCAGAATCTATCTCTGTATTTCTGCTAAGATGATCTCCATCTGCAGATTGATTCCACCTCTGCGCTTTCACATGAGCATATTCATCCAAATGAGCCTCCGAATTTCCTCCTTTATAATAAGTCCTTGAAGACCCAAATCCAGCACCACCATTATATTCTTTCCTCATAAATGGCTTTCCACCAATTGATGAATCTCTATGGGGACTGTTGGGATTATACTCCTGGTCTGGTGGATAGAATGCTGAGCTGCTCCAATTCTCATATGTATCTCTTCTCCATGAATTAACGGGCTTTCCTCGGTCCACAAAGGAGGAAGATAGATCTCTAGAGAAATGAGACCTAGAGCCCATCTCCAATGGCCTATTCACACTTGATGAATCAGAAGATGCTGAAGTTAATATCCTGTCAACCATTCTTTCACTATCCTCCCAATCACCTACATCTGTATTCCTGGATGCATCTTTTTCATTCACTATTCCAGGAATTTTCTCATCCACGACAGCTGGAGCATTATTACCACCTTTGGCTGCTTCAGCCTGCCTCCTAGCAATCCTTTGCTCCAATTCTAAAAGTTTTTGTTTGGCAGCTTGTTTCCTCCTCTCCTCTTCTAAAAGGATCCTTTGTTTCTCTTCTTCCCTTGCTAACCTCTGCTCTTCAGCCTTCCGCAAAGCTTCAATCCTTTCTTGTTCTGCCCTCCATGCTGCCTCTCTCGCTTCTTCTTCCAATCTCCTCTGTCTCTCTTCCTGTTCTCTAGCGAGTCTTAATCTTTCCTCCTCTTCTCTGCGAGCTAACTCCAATGCCCTTTCCTGCTCTTCAATGATTCGTTGCCGCTCCTGTTCTTGCATCCTCTGAACCCTTTCAAGCTCAGCCTCAAAGGATTCTCTAACAGGATCATGGAAATCAGTTTGTTTGAGTACATCCTTCTTCTTCTTGACCACCCCAACAAGACCAGCTGAAAAAAGATCCCTTCCATCTCCAAAATCTTTCATAAATGGATCCTCCAAGAAAGCCTTTTCAGTCTTTGGCAAAGTGCGCTTTTCTCTGCCAAAATTAAGCAGAGGATCATTAACAGGAAGCCCTTTACCACCCAAAGAAAATGAAGATTTCGACACTGAGCTCTGGTTAGAATCAACTCTATTCCTGTTATGTTGTTCATTACCAACATGACGATCCCGTGCATTTCGTTCATTCCCACGGTCATTGTATGATTCCATCACATTATTCCAAGGCTGTTTCCCACCCTGACCATACCCAACACCCCTCTTCCCTGGATCATCATGAACAATGTCTCTGAAGGGGGATCGAACATACTTGTTATCCTTCCCTACATCTCTATTCCCACTGGATGGCCTTGCAACAACACCATTTCTCTCATTCCCCGCATCTTTTACACCAAAACCATCCTTAGAAAAAGTTGAATTCCTCCAAGAATTCCCTTCCCTTCCCTCTCTAACAGCCATTCTATCAAAAGAATCCAGCTTAGGAACTTCACCGGAGGAAACCTTTCCAGCTTCATTGCCCCTCAGCTGTACCCTCTTATCAAAACCATGAGCACCATGCTTCTGTGGCAAAACACCAACCCTGGGCATATCAAAATCCCAAAAAGCATCACCCTTTGAAAATCCATGATCCCTCCCCTCTCTACTCCTCTCCAAGAAACCATGACCAGTATCACGCTCATCATCAGCCCAATCAGATCTCGGGTTCACCCGAACCAGAGGCAGAGGACCCGGAAAAAACTCTTCCTGATTCATCCTACCATGATTCTGGTTCATCCCACGAGAACCAACGAAACCACGAGTCTCACGGAAATTCTCACCAACAACATTCCCAGTTCGGGGAACGTTGAACCGAGAATTCACATCAACAACAAGTGAAGATGAAGAAGCATCCACATCAGCATCCTTCTTCTTCTGCTCACTCTCATCACCAGAATTCCTCTGTTTCTGATTCAAATTCTCATGGATCTTCTGATTCTGACCCGACCCGGGTGCAGACGAAGGCACCAACGTTGCCCGCAGAGAAGGGAAATCCTCACCTCTTAACACCGCCGTTGAAACAGGCACCAAGGCAGGCCCAACCGCCTTACTGGGCCGGCCCAGCCCATCAACGGCCCGCGATCCATCATCTGTAAATGGTTCTTTCTCTTGCAGAGCCACTGCAGCCGGCTTCGTCCAACCCATCCCGGAAGATGAGGGTCTCGAACCGCTTCCCGAACCGGCTCCACCGGCCGGACCACCACCCGATCCAAGCGAATCGAACCGTTCATGCTCTTTCCGAAGCGAAGGGAGGTTCAAGGGGGGTGGAACCGAAAGCTTCGGCCCAGCCTTTTGCGAACTCCGAGGCCTCGAAAGCACCGCCATGCCTCCGCCGCCATGACCGGCGGGTCTCGCCGTTCGGTTGGATCCGAAAGAGGTGGAATGTTGCCCATAGGATTTGTTGAGATTCACAGACACATATTTGGTGCCGGAATTTGCCATCGCAGTCGCACCACAAGTGCCAATCGCCAAAGCATGAAAACCCTAACCCTTTTTTGATTTTTTTTACTGTTTTTTTTTTCCAGAGGCTGATCTCGATCGAATTGAAACCCTCTAACCCTAACCCTAACCCTGATGATCAAAACGACGACGCACAAGGTCGAACGAAGAAGGTTTCAGCTCTGATCGATCGATCGATCTCGGTTTCTGTTTCTTTTTAATTATTTTCAATTAGGGTTTGAATGGGAAGGGGGGTGTAGGATAAGAGGGGTCACCTCCGAGGGCGTAAAATAGAGTGCGGCGGTGGTTAGGAGCGCGACCAATAAGAGAAGCCCTACTCTTTGTAATATGTGTATGGTCTGATAGAGAGAGAGAGAAAGAAACAGAA

>Rp-30

TTATTTTTATTTTATTTCCTTCAACAAATTAATAAATTTAGAAACTCCCAGGGGGTGAATTAGAAAACTGATGTCCGGTTGCATGTGAACAGACTGTAATGATAGTTTAGGAAATCATTCCAAGATAACAACAGTACACAAATCTATTATGAGCACAATTACTCCTTAGTTCACTCCCACACATGAGCAGTCATTTTATGTTCTCAAGATCTTTTATCATTTTGTTCGGCTGCTTCAGCAGGAGTTTTGGTAGTCATCTGATCAACTGCATGAACTGTACTCTGAAGCTCCTCCCATATGGCTTTATAGACCATCCTTTGCCTATTCACGGCTGATTGTCCCTCGAAGGCTGAAGATATAACGTCAATGCTAACATGCCGACCATCACCATAAGCATCTTTTACGATGACTGATTCTGCATTGAGTTGTTCCTTTATCTTTTTCTCCATGGATTGCATCAGAGGTGAGTCGATAGAGCCAGCGTCATTGACATGGGTGGCGCGTGAACTAAACTTGCGAAGAGAACTCATGGTGAATAATCTGTCCTGTCTATGAAGACCACCATAGCGTAGCAGAGTTTGTGAATTAGATGTGGATCTGAGGAGTAACATATGATTAGGAGCAGTTCGAAAGAGAATGGATTTGGAGGGTTGAACCAAAACTGGAAGAGCTTGCCGCAGCAAGTTAGTGTTGACTGAGAGCGACGACACCTGAGGGCGCATTGCTTGGATATCAACGCTGCTGCTGCTGCTGCTGCTGCTTCCCCGATACTAGGAGATGGATAGGGTAACTGTACGGTGCCGAGGTTGTCC

>Rp-31

GACCCCATTTTTCTCAAGGACGAAACTCTCAGAAACTCAGCACTAAGACGTGGCTGGTGATTATTATTATTATTATTATTGTGCTGACCACCAGGTGCTGCATTCTCCGACACCTTCCACACCTTCACCGACTTATCCAAACTCCCACTATACAAAATCCACCGCCGCTCATTCAACATCGCATCCGGATCCTTCTCGGCGGCCAGACACTTCACCGGTCCGGTATGTCCCGACAACACTGAAATGCACGTGTGATCATTATTCAACGACCTCTTCCACACACAAATTGCCATATCAGCTGATCCACTGAACACCAAACTCCCCGCTGTTGCCAAACACAAAACCGCCAACTTGTGACCCCTCAAAGCGCCACCATGTTCCAGATTCGTTTTCTTAACCCAATAATTCACCAACCCATCAGAGGAAGCACTGTAGAGAAACGTTCCTTCCTCGTTTATCGCCAATGCCGTAACCGCACACTCTTGTTTCAAAAGCGTCAGTGAGAAGTGATGTTTTGTTCCCTTTCCCTGCACCTCCCTCCGCCATATCTTCACCGTGCCGTCGGCGGAGCCGGTGAAGACCAACCCTTCGAACCCAACAACCAAAGCGTTAACGGCATCATCATGAGCA

>Rp-32

CCGGAATATCAACATCTCTCCCATGTTAAGAAGGTGATCATCATCATCATTATCTCATTAATTAACAAAAACTAAACAATTAAATGAAAAAAAAAAGAGAAAAGAAAAATCAAGAAACGACTAAAATTAACTAGGACCACCTCTTAATTACTCTATCTAAACATATCATTATTCTCTCTCTCTTGTTTTGTTTTTTCTTTTTCTTTTTTTTTACTCTCAAGAAGAAGAAGACGACCCATTAGTAGCAACGTTAGTCCCACTATCATTCTCGTAATGGTTCGGATCCTGAGATACGAGGTTGTTGTCGTCGTTCGCGTTACCGTTGGTTTCACGGCCACCGGCGCCGGCGTTGAAATTAACATGATCTTTCTTCCCAAGAGTGTTCTTATTGTTGTGCATCCAAACTTTAAGAACCCCTTTTTCAACACCAACCTCATTGCAAAACTCCATAACCATTTCTTCATCTCTCTTCTGCATCTTCCACCCTACTCTTTCAGCAAACTCATGCATCTTCTCCTTCTGCTCCTGCGTGAACTTCGTTCTGAAACGCTTCCTTGTGTTGGTTCCCGGAGGCGAGGTGAGGTTTGCCGGAGCTGCTGTGTTTTCCGGTGGGACTGAAAGACCTCCACCGGCGCCGGAGAGTGCTAAGAGCATGTGGGGTGCTGAAGGGTAAGACGAAATCGGCGGCGGCGATGCTGAATTGGGACTCCGGTTAAACGGCGGCAGAGGGTGGTGGCGGTGATGGGGTTGGTATTCAAAAACATGGGTTGCTATTGGTTCCTCTGGTTCCCGGCGGTGAAAATTCCGGTGACAGCCACAAGCGGCGCATTTGATTGAACTTGGGTCGTCGGCGGTGGTGTTTGGTGATGGCATGAACTCGCCGCAACCATCAACGGCGTGGCCACCTAAATTCGCCACGTGGTTCTTCAAACATTCTTTATAAGTCACCGCCACCGTGTGAGGGTAGTGGTGGTGGTGGTGGTGGTGAGGGTGGTGACGCTTGAGAACCCCATTTGAAAAAGACAAAGGCTTTGTGTTTGTGTTTGTGTTGATGTTTGTGTTTGTGGGTTGTTGTTGTTGGATCCGGGTCGGTGTTTCGGATTCGGGTTCGGGTGATTTTGCACTTGTTTGAGTGCTATTGTTGTTGTTGTTGGTGGTGGTG

>Rp-33

ATCTTCTTATTCCTTCTTCCTCTTTCTCAATTTCTCAACTTTCCAAACAGTCTCATGGAAATGGAGGATCATCATCATCATCATCATCACCATCATGAACAGCAACAACAGCAGTATGGTATCGCTGATCTTAGGCAGCTTGTGAATGGAGCACGGTCGACCCATTTCCCAACAATACCAACCCAACCCACAGCGGAGCTCTTCCCCGCCGGCCACCCGAATCTGACAGTGACAGCCACTCATCACCAACAACAACAACACTATGAGATGATGATGTTTGGTCGTCAAGTAGCAGATATAATGCCTCGTTGTCTTCATGACTTTGCTTCCACCGATTCTGGTGCTGCTGCAGCTACCAATACTGGTATCACTGTTGCCACTCCCACCACAAACAGTGCTTCAACTCCTCCACTTAGTGGCTTAGAGGCTGAGACTGTGGGTTGCATAGGAGGTGTTGATGCCTCCACTGGAAGATGGCCTAGACAGGAGACTCTTACTCTTCTTGAGATCAGATCTCATCTTGACCCTAAATTCAAAGAGGCTAATCAAAAGGGCCCCCTATGGGATGAAGTTTCTAGGATCATGTATGAAGAACATGGATATCAAAGGAGTGGGAAAAAGTGCAGGGAGAAGTTTGAGAACCTGTACAAATATTACAAGAAGACAAAGGAAGGAAAAGCAGGAAGACAAGATGGGAAGCACTACAGATTCTTCCGCCAACTTGAAGCCTTATATGGTGAAAACAGTAACCAAGCTTCAGTCCCAGAAACCAATTTTCGTTTCCAAACAAGCAGCCATGCCCCCTCTCAAACTAATCAAGAAATGTTTCAGTCTCAGAAGCATTGTGATAGCCTCAGCCTCACTAACTCCACTGAATTCGACACGTCATCTTCAGATGACAATGATCGTGACACTGAAGGACTCAAGGACAACGACTCCGTGGAGAAGAGGAGAAAGAGGATGAGTGGAAGAAGCTGGAAGGTTAAGATAAAAGACTTCATTGACTCACAGATGAGGAAGCTGGTAGAGAAGCAAGAGCAATGGTTGGATAAACTCACCAAGACACTGGAGCAGAAGGAAAAGGAGAGGGTTTTGAGAGAGGAAGAGTGGAGGAGACAAGAGGCAGAAAGGTTGGAAAAGGAACACAAATTTTGGGCTAAAGAAAGGGCATGGATTGAAGCAAGGGATGCTGCTCTAATGGAGGCTTTGCAAAAATTAACAGGAAGTGAGATAAGGACTCAATCTCATGAGGGTCTAATGCCAGCTAGATTTCAAAACCACAGTGAAAACCAGAATGAAGATGGAAGTGAAATACTGAATAGCACAGCCAGAGGTGCTGAAAGCTGGCCAGAATCTGAGATTACAAGGCTTTTACAGTTGAGAGCTGAGATGGAGACAAGGTTTAGGGAAAGTGGGTGTTCAGAAGATGTTATGTGGGAGGAAATAGCAACCAAAATGGCTTGTTTTGGTTATGAAAGGTCTGCTTTGATGTTCAAAGAGAAATGGGAAAGTGTCAGCAACTGCTCAAGGAATGCCAAGGAGGGAAACAAGAAGCGCAAGGAAAACTCAAGAGGTTGTTTCTACTTCGATAACAATGACCAATCTTCTCTATATAATCAAGGAGGTGGTGCCTATTGTGATATCAACGACCAAAGACGAGAGAGACTACAAACAAATGATGGTTCTTCACCTTCAAATTCTAATGTGGGAAATGCAGTTGCTGCTGAGAGCTGCTTTCCCTTCTTGATGACTGAGGGAGGAAATTTGTGGGAGAACTACAGCTTAAAGGTAAATAAAGCAAACCAAAACCAATGAATGAAATAAGGTTGACTCATAAGAGGCACGAATATTTCATAGCCTTTTATCTGAAGTGATCTCACACTATGTATGCAAGAGCTAACTGAGGGTTCATGTATAGAGACTGATGATGATTAGGTGGTCAATCTTATTATATTTATGGGACTTTAGGAGAAAAATAATGTATTCAAGTCACATAAGTTTAATGAGGGAGAGACACAATGGCGGATATCACTATGACACTACTTCAGGTGAGTTTCTCTCTTGCCAGATTATCTTTTATGCGTGAAAATGGAATTGCATTTGTATTACTTCATATGTTGCATCTTCAGTGATGCTTAAATCAGTTGGTCACTGTCTTTCTCCTTCCTCCTCCGTTC

>Rp-34

TATAAATCTTGTCTAATGACCATAAAATGTACGTGCATATTCTTCCTTGTATGCCTTCTTTGTTATCCATTTGAAAGGCAAAATGAAGGTTAAAGAAAGGGAAACTAACCTCAAATAACAGGGAATAATAAATTTTCTGGGCTGAAAAAGATGCCTACAGATATACAAGGGATATTGACATTTTTCTCAATAAACACAATATAAATCTGCAATACACTACTTTAAACAACACTAAGTCACAAATTTGCCGCTGTGATGCGGCTTGAGCCCGTGATATTTGTCCTGAGAGATAGGAGTCTAGATTTAACATTGCTAACCAAATGTGATATATCCTGCTTCCAGGACCAAGATGGTTCATTTAAAGCACGTATGGAATTGAGACAAACTAGCAGTGTTCCACCTTCATGTAAAAGAACCGTTAACCACAATGGCAGAAATCCCAAAACTGACGGAAGAGAAGCCATGATAATGCACGACAAAGCCAGGGCAACATTTTGTTTAATCAGTGATGTAGTTTGGCGAGACTTGGCGATACAAAATGGCACTGCAGAAATATTTTCTCGAAGCAACAACACATCTGCAACAGCTATGGCAGTTGCACTAGCACGATGAGCAAGCACAATCCCAACAGTTGCAGCAGCAAGTGCTGGGGCATCATTGATACCTTCACCAACCATGATTAAGCCTCCCCCCATATCTCTTGAAATATCCTTTACATGTTTCAGCTTATCTTCAGGCTTCAGGTTGCAATAGAACTCATTGATGCCCACAGCACTTGCAACTCTCCTAGCACTAGACTCATGATCACCGGTCAACATCATTACACGAAACTTTGCTTCATCTTGCAATTCTTGAATAACATTAAAAACCCCAGGACGAGGCCTATCCTCTAGGTGAATCAAGGTTACCTTCTGATTAACTGAAAGGGCAGCATGAACAAATTCACTTCCATAAGAAGATGTATTAGCAGCTTCCTTGATCTTTTTTGATTCATCTTCAGATTGACAAAATGAAGTGATGAAATCTATAGACCCAAGTGATGCTTTGATTGGTTTAGCACCTCCAGTTCCTGACTCAATGCTGTTTAAAGTAGCAGTAAGGCCTCTACCAGGAAAGTACTCAAAACTTTCAACAGAAACAGACGGGAGATCTTTGCCTTCGCTATGGTCAACCACAGCCCTTCCAATGGGGTGAGTAGTGCCCTTCTCCATTGCTGCAGCAACAGCAAGAGCTTCTTTTTCACAGGTTGGAATGCAACAGGGAGGAATGTTAGATTCATTGTTTCCAAGGTGATGACCATAAATGGGCTCCATTGCCTTGAATACAAGTCCACCAGTTGTTAATGTCCCTGTTTTGTCAAATGCAATAGTATGACAAGAAGCTAGAGCATCCAGAACATGTCCACCTTTGAGTAATATCCCCTTTCTTGCACAGGAGCTGACGGCTGTAGCATATGCCAATGGGGCCACAGCCAAGGCACACGGTGATGCTGCCACCATGAGCCCTAGGGCTCTGTAAACTGAACCTCTACAAGCTGGCGTACTAATGAATGGCCACTTAAATACAAGTGGCCCAATAACAGCAATAGCAATTGATAGCACCACAACAACTTTGCTGTAACGTTCACCAAATTCATCCAGCCATCTTTGAAGTTTAGGTTTATTTAACTGTGCTTCTTCAGTCAATTGCACAATTCTGCTGAGAGTTGATTCCTTCCATGTCTTTGTTACCTTCACAATTATCCTCCCATCTAAGTTCCTTGCACCCCCAGGAATTCTATCTCCAACTTTGGCCTCCAAAGGTTTGACTTCCCCTGTCAAGTGCTCAATGGTAATTGTGGCACAGCCGTGAAAAACTTCGCAATCTACAGGCACAGACTCACCAGCACCAACAAGGATATATGATCCTACAGCTACATCATGCACAGGAACACTTTTGTATGCCAAGTCAAATGTATTAGGAAGTTTATCATCATTCGTATCAAGAACAAGAGCAAATTCTGGATGATTCTCCTTCAACTCTTTGACATCGACCATTGAACGGCTGGTGAAATACTCTTCGGCTATATGCGCCAGATTAAACATGGCTAGAAGCAACCCTCCTTCCAAAGAGTTCCCCATAAATACTGATGCAAAGGCTGCCATTGCCATTAATACATGGATGTTCACTTTTCCACTACTAATTTCAAGAAGAGCATCAAGTGATGCAGAGACCCCAACCAAAGGAAAGGCGATGACGATGAGAGTGTTTTGAAAGGGTTTGATGAGGGGTTTGGGCAAGGTATGAGGGCAAATGGCTGCGGCCACAAATAAAGCAGCGGAGAAGCAGCATAGATGCAAATGCTCCCTCAGGATATTAGCCAAGTCCATCCATCTTGTGGCCTTGGCGAATCCGATTATGGCTCTTTGAGGTCCGGTGAGATTAGCAACATCGATGCCGTGGTGATGGTGATGGTGATGGTGATGGTGGTCGTGGTCGTGGTCATGATCGCGGTGATTGTGGTGATGGTTACTGGTGGATTCAGCGACAGAACGAAGTCTGTGGTGATGGAGAACTAGGAATTTGGAAGAATAGAAAGGTTTGATTGAAATTGGGGGTGGCCGAAGAGTAAGCTTGGAGGATCGAATTATTGTAGCTCGGGAGTAAATATGAAGTGATTGGAATTTGGTAGTGGGGATTGAATATGAAACTGCTTCCATTTTGAGATTGGAACTTCAAAGAGGAGCTTCAAGCCATCGTGGTTGAAAAGGAGAGGAAAAGGATCGGAGTTTGTAGGAAACTATGGCAAGTACCATTCAGTTC

>Rp-35

TCTGTCAATAAAGAACATAGCCGAAATTATAATCAATACCCCTTCTTTAAGTTGACTGAATCGAAATAACTTGATATCTATCAAGGATCAGATCAGGGTAGCCACATGAGCACGAAATTAATAGAAACAAAGGTAGCACCAGTTCGTTTTTTTTTAATTTAAATCCCCGCTTTAATTAATTCTATCCACCGATGAAAGCAATATCATTCATTTTTATTGATTACTAAAAGTAAATTGATA

AATGATACCTCATTTGATCGGAGATGGAGAAAACCCATTGATGTATCCTTGACGAGCAGTGTCAGACGTGGTAGAGCAGTGTTGTTATACGTGGTAGAGCGATAGATAGGCAATAAAGAAAAAGAAAGCAGAGAGAAAACGAGCACACCACACCACACCACACCAGAGGGAGATCCCGGGCAATTCCCCGTGCAATCTCCCCCAAAAACAAATAATCTTTTTTTATTTATTTTTTTGACCTGCCATGCAAGCGCACCTGTCATGGCAAAGGTCGGATGACAAAATCAATCAGGACTCAAAACCTCAAACCGCACAGAAAGGTTTTTCATCCCGAAATCTTCTACATCCACATACTTTCCCCATCAAAGCTTCCCTTCATAACCTAATTATACAATATCTGAGATCTGCAACATATGTAGCTGATGGAACACAATAATCCATAACAATCAAGAGAAATCTGAGGCTGAAAATCCATAGAATCCAAGATCGCAGCATCGCTTGAATTTAACGGTGGATCTGCTTCCTGATCATCTCTTCTCCGTCGTACCTATGTACCGCAGATCTGATGCACCAAGCCTCAGATCCATGAAAGACCCGAACGAAACCACCATCGGCGCGTAGATCTCAATCAGATCCATACAGATCCGGTTACGAAAAACGTGAAAAAGAAAGAAGAAGAACAAGATCTAGATCTCTTTGGGAAGAGGAAGAACGAAGAGTGAAGAAAAAGATGAAACCCTACAAG

>Rp-36

TGCTGCTGCTGCTGCTGCTGAGGCTGCTGCTGCAGTTGCTGTTGCTGCTGATGCAGCTGAGGCTGCCCCATTGATTTCTGTTGCTGCATCAGCTGGGACAGTTGCTGTTGGTTCAGGGTCATGGAGTTTTGCTGCAGTTGAGTAGATGAAGCCAGCTGCTGCTGCCTCTGATGTTGAAGACGCAACGGTGAGGCAGGGCCAGACATTGCTGGCATTTGCTTAAGCCACTGTTGCTGTGTTAAAGAATTATGAATCATTGCAGGCTGGCCATTCTGAGACAACCCAGACAACTGGGAATTCATAAAAGCAAGTGATGATGACCTTGGTAAACCTTGAACCTGCGACGAATTAAGTGAATTCTGCTGTGATAGCTGCTGCCTGATTTGTCCCTGAGTCATTCGCAGCTGCTGAGCATAAGCAAGTGCTCCATTGGCTCTTAATTGCGAGCTGAGATTCGGCGACCCCATCATCCCCATTGCTTGCATTCCTTGCAGTGGCTGTCCAGGATTATTAGCCGAAGAGAATTGAGATGGCTGCACTAACCCACCTTTCTGCCTCGGCGATGACAATAGGTTAAACTGTGCTCCGGCGCTGGACAACATGGGGAAATGACTATTCTGTCCAATCAAGGCCGACCGCGACAAATTGGAACCACCTAATTGCTGCTGTTGACCGGAGGCAGAGCCACCGAAATTCATCTGCGCGCCGTAAAGTCCGGCCTGCTGTCTGGCGACGTTGAACTGCTGCTGTTGCTGTTGATTGAGGCGTGACATGGAAGGGGAACGCTGTAGGGATTGCTGGAGTTGAAAGGTGGAGACAGAATTAGGGTTAATGTTCATGGCTTGGCCTTGTTGTTGGGTTTGTTGTTGTTGAGCCTGGTCGATTTGAGGGAGTGAAGGGATGTTGTGGATTGAAGGGGATTGTTGTTGCTGAGAGAGGTTTGAAGAAGGGGATTGGATTGTATTAGGGTTTGGATTGGGATTTTGGGGTTGAGGGTCCATGGTTGAAGGTGATGCCGTTTCAGCCATTGATTTTGTCGTTTCAGCTCTCTCTGTTATGCTGATTACTGCTGCATTGGCGAACTCAGCAACAACGATGATCAAAGCTTCGTTGAATCGAATCGAATCGAATCGAATCGAAGGTGGACGGTGATGATCCCCGA

>Rp-37

AAAATAAAAATTTGATCAATCTTGATAACAATTTGGCTATAGCTCATCCAAAATACGCGGTTTGAATGTGGAAGCAAAAACCAAGTTACACATAATGTAAATACTACACTTAACGGCAACATGAAAAAACAGATCTGCGAGTCAGATTGTAAGGGCTAAAAATATATTTTCTTTTCCTTTTTAATTTCCTTGTATTTCTTTCAATCTTTTTGAACTCCAATCCTTGATGTTTGTCTACCTAATAGATCCACTGCGTATGGATGAAACCTTGCAAATGCCAGCTGATGTGGTGAGTGGAACATTTTTGTAGCATGCCACTAATTCCTCATTGGTGTGCAGATCGACTTGCAATGTCTCCCAAACTTTCTTCTTTGGATTTAAGAGTTCATCAGGATCACCTTCATACCCTGGGAATGTAATTCTTTCTCCAACATTAGCAGAACTGGGAGGTTCAACCAACTCGACCTTGGTATGATCATCATTGGAAGCAGCAAGAACCATTGCGTGGGATTTAATGCCCCTCATGGTTGCTGGCTTTAGGTTGCAAAGAACACAGACCTTTCGGTTCTGCATTTCATCAAGTGGTATATACTTGACAAGTCCGCTGACAACAGTTCTGGTCTGTGCTTCACCAACATCAATCTCTTCAACATATAGTGAATCTGCATCAGGATGTTTTTCAGCTTTTTTAATGAGACCAACCCGAATATCAAGCCTTGTGATGGTAATATCTGGTTCAGCAGCAGCCTTGTTTTTGGCTTCATTTGATGATTTTGCTGATGATTTTTTCTTTGCATTGGCATCTGAAACTTTTGTTTTCTTCAGTTGGTCAGCAAGATTTTTAGCTTCCGCCTCTTCACGCACAATCCTGTCAGCTTGACTTCCTGCGAACTTCTTCCTGTAGAACTCCACTTCTTCATCTTTCAGCTCCCTGACCAATGGCTTGGGTGTTCCGATTTTATGACCAGCACTTAAGATGTCCCAGGGTTTTTTTACCCTATCAACATCTCCCTTATCATCACAAAGTGAAAGATGTTTCTCCACGGACAAATTTAGCTGCTTAAATACCTCAAGAGTGAAAGATGGCATAAAAGGTTCCAATAAACAAGCAAGAAGATAAACGATCCCAGCTGCAGTCTTCATAACAAGGGAGCAGAGAGATTGGTTTTCCTTGTAAAGGCGCCAGAATTCAGTTTCCTGCAGATATGCATTCCCCTCGCCGGATATGCTCATTGCAATTTTCAACCCTTGCTTTAGTTTAACCTTCTCCATTGCTTCTATGTATTGCTCCAGATATGCAGTAACTTTATCAGCCAATTTTTTGGTTGGATCATGGGTGTCACCACTTACATCATCAGGAACAGTGGGAATAGTGGAATCATATCCTTGACCTGCAGGTTTGGCAATAAAACTCAAAACTCGGTTGATGTAGTTGCCCAAATTATTCAGCAACTCACTATTTAATTTTGCTTGCAAGTCAGGCCATGTAAATAATGTATCAGATACCTCAGGCCTATTTGTGAGCAAGTAATACCTCCATGCTTCAACTGGAATATTAGTCTCTTTTGCATCGTTTCCAAATACCCCAATGCCTTTGCTCTTAGAAAACTTCCCTGCTTCATAGTTCAAGTATTCAGTAACACTAATAGTCTTCATTAAAGTCCAATTTTCACCAGTTCCAAGTAGAGTAGATGGAAACATCACAGTGTGGAATGGCACATTGTCCTTGCCCATAAACTGATACAACTCCACGTTCTCTGGGTTTTTCCACCATTTCTCCCAATCAGGTGTGTAGCATGAAGTGATTGAGACATATCCAATAGGTGCGTCAAACCAAACATAGAAAACCTTATCACTGTATTTTTCATGTGGAACACGAACCCCCCACTTCAGATCCCTGGTAATACAACGTGGCTTTAATCCTTCCCGAAGCCATGAATTTGTTGTTTGGATAGCATTCTGACTCCACGATCCAACCACAGACATCTCGTTGATGTATTTTTCCAATTTATCTTTCAATAGAGGGAGTTCAAGAAACAAGTGGTCCGTATCACGAATACGAGGACTTTTTTGACAAACCTTGCACCTGGGATTTTTCAATTCTGTTGGATTTAGAAGCTTTCCACACTTCTCACATTGGTCTCCTCGAGCAGAATCATACTCACACCCTGGAGTTGGGCACGTACCCTCCACGAGCCGATCAGCTAAGAACCTTTCGCATGTATCACAGTAAAGCTGCTGCATTGTGTTCTCGGAGAGCCAATTGTTCTCAAATACTTTATGAAAAATTGCCTGGCAAACTTCAGTTTGCTGAGGGGAGGAGGTCCGCCCAAATTCATCGAAACTTATATTGAACCAATTGTAGACCTCCTTATGAATAGCATGGTATTTGTCACAAATCTCTTTGGGAGAGCAATTCTCTTCCATGGCTTTAGTCTCTGTTGCTGTTCCATACTCATCAGTACCGCAGATATAAATAGTATTATAACCCCGAAGCCGACAGTAACGTGCAAACACATCAGCACTCAACACAGATCCAATAATGTTGCCGAGATGAGGGACGTTGTTGACGTAAGGTAAGGCACTGGTGATGAGGATGTTGCGCTTTCCTTTCTCTGGAAGCTTCGGCACTTTCCGATCCTCTGTCGTCATTTTATTTTACCCTTTCTAATTTGGTACGGTGGCTCGCCGGAGAAATTAAGCTGCTCAGCCGCCAGAACCACCACGAGAGTGAGAGTGAGAGTGTGAACGAGAACGAGAACGAGAACGAGGGTAATGGAAATAAAAAGGGTGAGGAGT

>Rp-38

CTGATACATAAAACTGCAAAGAAAAAGGGATCAGAAAAACAAAGCAGAATCATGAGGCAACTATGGATCCATTAGAACAGTAATTCACCTCCCATCAATTCCAAAATTTTTACAGCATAGATATCATTACGACCTAATTAATTATCATTATTCATAAATTACCCCTCCCCCGCACGGTGGAGAGAGTCATGATTAAGCCTTTATCGTCATCAACCTCTCATCTCATACCCCACACCATGCGAGCTCCAACCCTTCTCTCTCTCAATCTCTCTTCAATTTGGCTCTCTTCTTCACCATGCAATCCAATTCATTGTTCCATTTTTACAAACATCTCCATCTCTCCATTTCTATACACCGTGCAAAATGTCTTATTTTTCCATTCCCTGGTTTCTTCTTCTTCTTCTTTTATTTCTCTCTCTC

TCTCTCTCTCTTAACTCCACTCTTCTGAACAATACCACTGAAGGGTCGGTGCTTGTTCATCTGAGAAGAAATTGCAGGCCTCATCTGCACTGAGGAAATTGTGCTCTTCCATCTTCACGTAGTGTGTCTGGTACTGCTTCTGAAACTGGAAGCAATTCATGGAAGATGGCGATGACGATGAAGAACAATTGAACTTGAGAGAAGATGACCCAGGAGACATCAAAAGGTTGTGACTTTGAAGAACCCCAGATGAAGAAACAGCCACGTTAGGACTATTGTTGTCTTCGTTCAAGATTGCGCTAGAGTCGCTATCTGAAGAACCGTCTTTGAAATCTGTAGGGAAGAGCGAAGCAGCTCCCACCCCATCACTGTTGTTGAAACACTCATAATTCAAGTCCTTTGAATCGGATCCAGGAATCACGGTTTCAGCTGGTGGGTTCATTTTGTATTCGGAGTCTTGCAGGGTTATCATCTCTTCTTTCACTGAAGCATCGCTTTCAGTGTTCTCTTCTTGCAACCTTGATTTCAATTCCTTAACCTCTTTGAGTAAGGCTTCGTTGTCATTTTTGAGGGTGTCATAGTTAAGTTTAAGTGCTTCATAATTGGCTTTGAGAACACCATAATCTCTCTCCAACTGCTTGGTTTTCCATCTAGCTCGACGGTTTTGGAACCAAACAGCTACTTGTCTAGGTTGCAAGCCAAGTTCTTGTGCGAGCTTCACCTTTCTGTCAGGTTCAAGCTTGTTCTCTACCTCGAAGTTCTTCTCCAAGGCCTTCACCTGATCCACACTTAATCTTCGCTTCTTCTCAGTGTGATGTCCAGATTCTTCCACACAACCTTCCTCATCAAGTCCTTCCAACATGGATTGGAACTCCCTGCCATACATGTGGTTGTTCCTCGGACTGTGTTCGTCTGTTGGTGGACAAATCGTCATGAGAGCACCCAAAGAATCAGAGCTTCCAAGTCTCTTCATGAGTACTAAAGCACGAATCTTATTAGATCAAGGTCCTTAAATTTGATCCCTTTATTGTTAGTCAATGTATATTAAGATCTCAAGAAAAAGCTGCGAGGACAAAGTTTTTCACTTAAAAGGGAGCCACTGAATAGGAATTTTGGTATCTTAAACAGCGCCAAGTTGCACAGTTCTTTAAACAGCCACCATACAACCACTTATCTCCATTAACTCCCCCGTGTTCCTTCTTTCTCAACACTACTACTCCCTCTTCACCCATCAAACACCCAGAACAAAACAAACCCACACAACACAACCAAGCAAATATCTCTCTACAAACCCACTAATGACCAATATCTTTCTGGTCTCTTTTTATTCTTCTTACATCATTACACAATCAAATTTATAGCCTAGGAAGGAAAATAAGACAGGGAGGGAGAGAATGGAACCGAAGAGAGAGAGAGGATTTTGGGAAGGAGATTAAAGAGAAGGGAGAAGTGAGACTAGAGGGTGAACAAG

>Rp-39

CCGTGAAGTCCGATTCAGGCATTGAGACAGACCTTTAATATTGTATTTGTCAAATGCATTTCTAAGTTCTCTTGCGTTTGTGAATTCCCAAAAATGAGGAGATTGAAGTCCGTGAGGTGCATCGGAAGCAGTGATAAGTTGCGATTGGTGCTGCAATTGCAATTGTGGGAGTGATAGAAATGTAGGACGATTGAAGAATCGCCCACGTTGCACAATCTTATGCATATCCTTTGGTGATGAGGAGAGAAAGAGAATAGCTCCGATTAAAGAATGTTCCGTTCAGAAGAAGGAAAAGGAACCTCTCTATGGTATTGCGATGAGTGTTGTTTGTTGGATTAAGGAGGGAAGAAAACAAAGAGGAAGAGGAAGAGGAAGATAAAGATGTTGAGGGTTGCAGCTAGGAGGCTATCTTCTCTGTCGTCATCTCCATGGAAGTCCAACCACGCCGCTTCTGCCTACGCCTGTCGGAACACCGTCGTGGATGATGACCGCAGATCTGCTCCTTCCCACGACGTTCCTCATTTCCTTCTTCCCTTTAGAGGTTTTGCTACTGGATCACTGATCGACTCAAAAGAAAACAGCCTTATTCCTGAGATTCCAGCAACTATTGCTGCTGTTAAGAATCCTACTTCTAAGATTGTTTATGATGAGCACAACCATGAAAGATTTCCCCCAGGTGACCCAAGCAAGAGGGCATTTGCATACTTTGTCCTTACTGGTGGTAGGTTTGTTTATGCCTCTTTGATCCGTCTCCTTATACTCAAGTTTGTACTCAGCATGTCTGCTAGTAAGGATGTTCTTGCTATGGCTTCACTTGAGGTGGATCTCTCCAGCATTGAGCCCGGCACTACGGTAACCGTTAAGTGGCGTGGAAAGCCAGTATTCATCAGGCGTCGAACAGAGGATGATATCAAACTGGCAAACAGTGTTGATGTTGGATCTCTTCGTGATCCCCAACCTGATTCAGATAGAGTCAAGAACCCAGAATGGCTCATTGTGATTGGGGTTTGCACACATCTGGGTTGCATTCCCTTGCCAAACGCCGGTGACTTTGGTGGATGGTTTTGCCCATGCCATGGTTCACATTATGATATTTCTGGCAGAATTAGGAAGGGACCTGCACCATACAATCTGGAGGTACCAACTTATAGCTTCATGGAGGAAAACAAGTTGTTGATTGGTTGAAAACCTCAGGCTTCTCCCTGGTGGAATGAATCCACAATTTTTTTGATTTATTGGATAATGTCGAAATGCACCATGGACATCTTTGGTCTTGGATTCCTTATTTGTTATTTTAGCAAGTGTTTATGTAACTCGGAAATTTAAGTTTTGCTGTTTGACAAGACTTGTATCAATAATCGCCTGGCTTTTTGCTTATCATGAATGTTCGAAAGCTGATAACTTAAGGCATATTTCTTTGTATTATGAATTATGAATACCATGAATAACGTTTGGAAATCTGCTTAGCAAATGAGTTGAGGAATATGGCATTTTGCTTATGCGTACAACATCATATTGGGTATCCTACGAATCAAACAGTAGCCACTGAACACATTGGAAACACATTTCTTCAAAAGTGGGAATCATCATTACTAGATTGGTGTGCCTGAGGTGAATCTCAAAACTGTCAGCTGGCTGGTTGAAAATTCTGGTTTACAACATTTGTTCCAGGGGACCAGTTGTGGCAATTTTTTATAGGTCCAGTCGATCATTCCTTTACATTCTGTGCTGCCATCTGGTAAAATGGGTGATCATCTTTCGAAGAGGCCTTTAACATAAACCATGCCCTTTTCACTAAACTTGAGGTCCTTTCTCAGTTGCTCGACTTGCTGGTCATCACCTTGAACAATGATCTCTGATAAGGTTCCATCATCTTCAACCATCAGTTTCACCTTAATCTGCTCCTTTCTGGATGCTCGAGTCCAGTAATAAGCACCTGCCAATGGACTTAAGATGGTAATCCAAAACCAGTTATTGCCAACATCAGGGACTGTTATGGTGAGCACAAGGGCCACACTTGCTAGGCTGATGCAAGTGCAGAAAGTTAATAGCGCTGCTTGGCCCTTGCTGGGAACCATCATTCCTTCAAAAGTGATTGTTTCTCCTCTATCAGTCACTTCAAAGTTGTTACGAGTGAAGAATGATAGCATCTCCCCTGCAATTTGATTTGGTGCCTTCTTTTCACCTTCATTTATAAATGTCTTCTTCACAATTTTGGATTTGACAGAACGTTTGATGAGTGACCAAAGGCCAGGTACAGAGATGACAAAAAGACCCAGAGAAGTATAATAACTGGCCATAGAATAACCAGCACTCTCTGTAAGCAACAGAAGAGAATTGGGGTCTAGTTGTTGTTGTTGGGTGAGGTAGTCCACAAGAGCTGAAGTTGAGGTGGCAGGGTCATGAAGGGACACATGAAGCTTCTTTGGTCTTGAAGAGTGTTGCTGCCATCTCAGCCATGGTTGTTGTACTTTGTGGAAGTTGGCATGAAATGGGAGAGAACTGAGAGGGTGTGGATGTGGAGATGACAACATCTTTGCTGCCATAGCCTCTCTTCCAAAGAATGGAT

>Rp-40

AGTCATTGGACATCCCTCCATAATGTTGTTCTAACTACAATTATATCTCAATGCTAATAATAATAATAATAATAATAACAACCCACCATTACTACTATTGCCCTCTCTCTCCCTCACTAAAAAAATCAACCATGTCGAGCCCCGACGGTACCGCTAACGGGTCAATGATTGACCCACAGCGTCAGCAACAGTTGGCCGTCAAGAAACCCCCCTCTAAGGACCGTCACAGCAAGGTTGACGGCCGTGGACGCCGTATACGCATGCCAATAATATGCGCCGCCAGGGTGTTCCAGCTCACGCGCGAGTTAGGCCACAAGTCCGACGGTCAAACCATCGAGTGGCTCCTCCGTCAAGCCGAACCCTCCATCATAGCCGCCACCGGCACCGGCACCACCCCCGCCAGCTACTCCTCCGTCTCCGTCTCCGTCCGTGGCGCTAACTCCAACTCCCTCCCCTCCCCTTCCTCCACCTCTGACCACAAGCCTTCCTTGCTCGCTCCCACTCCCTTCATTCTCGGCAAGCGTATTCGCACCGACGATGATTCCTCCCCCAAAGACGACGCCGTTTCTGTTGGTTCTCTCGTTGGACCCGCTACCCCTGCTGGCTTGTGGGCCCTTCCTGGTCGACCTGATTTGGGACAATTTTGGAGCTTCGCCGCCGCCGCCGCTCCTCCGCCGGAAATGGTGGTGCAGCCTGCCGCCATGTCGGTGTCTCAGCAACAACAGCAGCAGCAAGCGGCGTCTTTGTTCGTTCATCATCATCATCATCATCAGCAACAGCAGCAACAAGCCATGGGTGAAGCTTCGGCGGCGAGAGTGGGAAATTACCTTCCAGGGCATCTGAATTTGCTTGCTTCGTTGTCCGGTGGACCTGGAAATTCTGGTAGGAGAGACGATGAGACTCGTTGATTGGTTGTTATGGTCTCAAGTTCAGGTTATGTTGGTGGTGGATGGTGAAAGGTGGTGGTGTTTTCTTCGTATATATAATATATATATTCTGTGTTATGGATGCTGCTTTGTTGTGTGTGGGAAGAAGAAGATTCTGAGTATAATTTTAGGGTTAGGTTTTTTTCTTAGCTTTGAGTGGAGAAGTTCCATCAATTAGGGCTTTCATTACTTCCTTCTGGTTTGGAAAATTAATTAGGTTTTTAGAAGTTCCTAAATTTGTTTAGGTTGTTCTGGGGTTTTTGATGGAGGAGAGAAAGGGGTGTTAATGTTTGTGATGGTAGTGGTGATGATAAATTGCTACAGATTGAGGGCTGGATTACCTTGATCAGTGTAGTGTTATTGTGCAAGTTGAAGACCAAAGCTTGGAATGATACGGGAGATTGTATTGTTGCTTGCTTCTGTTTAGTGGGTTTTTGAGTTTTGACTGACAGTGCTAGTCCTAATTGGGTGGGTCTAGAAAAGTTTGGCGCTTATTGCTGCAGTTGAAGTAATTAAGGTTTCTTAGCTGAGTGAATTGTGTTACACTCCCAAATTGTTGGAGTACCCTACTACTACTTTTTCAAATTCATGTCTATTATGGCTGCAATTGGGGCATCTTTTGCGGGATTGAGGTCCATTTAAAGCGGCCATGTTGTTATGAATTTGGCACAGTTGCCGGTGAGTCATGACCTCTCTATTTCTCTCTCTCCTCCTCCACTTTCACGCGCTCCTTGTTGGTCTTAACTTGTTTCAGAGGTGATGATTGGTGTGTGAAAGATAAAAGCTCTGGTTGATTGTACTCAGTATGACCTTTGTTTTGAGATTTTTATCTACAATTCTTCTTAGCCATTTCTTTAGATTGATCAGCGTGAGAATAATGTTTTCTTGTATGTAAGTGGATTGTTTTGGGGTGCATGCCAGTGATGTTAATAAAAAAACCC

>Rp-41

ATTAACTCACCCAATTGCACACTCTACCCTCATCTGCAGCATATATAACAACACAGCTAAGAATACCAAAATAGATCACAACAATCAAATGGCCACCACCACCACCACCACCATAGTGGTAGCTATTGCTGCTTTAGCTCTTGCTCTAACATGTCATGGTGTGTCTGCTCAGTCCCCGGCACCGGAAGCCGCCATGGCCCCATCTCCGGCGGGCAGTGACTGCTTAACAGCACTAACAAACATGTCTGATTGCCTAACCTTTGTGGAAGATGGGAGTAATCTAACAAAACCAGACAAAGGGTGTTGCCCTGAGTTTGCAGGGCTGTTAGATAGCAAGCCCATATGTTTGTGTCAGCTGCTAGGGAAATCTGAATCTATTGGCATCAAGATTGATCTGAACAAAGCTCTTAAGCTTCCTTCTGCTTGTGGTGTGGATACTCCCCCTGTTAGCACCTGCTCAGCTATTGGAGTCCCTGTTTCCTTGCCTCCATCACAAAGTGAAGGTTCACCAAGTATGGCACCAGAAGGATCAGCCACCAGTCCTTCAAATGCAGCTGCCGTTCCTAGTTCGGGTGGTCCTAGCCCTTCTTCTGACGAAGCTACTGCCAGCCCTTCTAACAACAAAAATAGGGCTTCAAGTATTCAAGCCTCTGCCACGAATTTTATTTTTGGCTTGTCTACTCTTTGTGTCTCCATATTCTTCTGATTTACACATAATCCTAAGTCATTAATTTCATAGAGTCTTTCACTTCCTTATTATTTATATGTTGAAAGCTGCTTTGACTGTAGCTTTTGTTGTAATTTGATGCACTCTGCCAATCATTATTACTACTACTATTATTAGTGAATGACAAACTTTAATGACGACCTTCTTGCTACATTACAAGGTTTATTCATGAGAGTTGGTGATAAACTTTGATTCGAAAACTTTAGATTTAATTAAATTGATCTTCAGACTCGATGCCTTACAAAACTTCTCCAGGAGGCTAGACATGTAACTAACTTGGGAGGATTTAGTCGTAGTAAAAAGAGAAGCACGTCATCTGCAAAAACAAGTGGAATAAACTTAGACCGTTGTTAGAAATTTGAATATGTTGCTAGAGGCCCAAATACACTACATCTGAGATAGCAAGGCTAAGTCTTTCCATGCAGAGAACAAATAA

>Rp-42

TCACCTCCATTTAAAGGACGACAAACTCCAATCTTCACACTCTTCGCAATCCTCACTCTTTGCTTGTAATCAATCAATCAATCATGATGTCGGTTTCATGTATCATGACCAAAATAACCACTCCTATGTTATGATGTTAGCACTGAAAGTAGGCCAGGTCAGACATTGTAGTTATTTGTTTGTTATTTTCATTGTTGGCTTCTGGGTAAGCATTTCAGTTGTGTAATATGCGTTCTCAGGCTACTCTCTCTAACCTGCATGGTTGTAATCATCGACAAAAGATCTTTACACTCTTCAAGCAAAACACGCTCCTTGGAACTTAAGTTGGCAACTTCCACCACCAGAGAATTTACGTGTCCAACCTTTGGTGACAATAGGCATATGGAGGATGCCATTGCCTGCATCACATCCATAGCTGAACAGATGGCCTCCTTCAAATTCAGCAGGTCTGCCTTGGCCCCACCAACAACAGGAAGGCGAAGAGTGCTGGCCCTTAGTGCTTCAATAGCCCCTGAAAGTGAACCAGAGTACACCCGATCCAAAATAGCCCAATCTTCCAAATAGATCATTTGCTCCTTCAGGATGGATATTAGCTTCAACCGCTGCTTCAGCAACTGTAACTCTGTTCTTTTGGCTCTAACTGATTCTCGTAGTTTCGAGGTAGCCACCCATGCATCGTAGAGGCTTTTCTCGGCATTCAATGTTTGTGCAGAGAGTGCAGCATCTGCTCTGGCGTTCACAAAACGCCATTGCAATAGCCTATTATGAAGGAGTCTCAATAAATGCGCATCAACAATCCTGTTCTCCCCTATCTTCCCCCTCGGAACATCAACAGCAAAACTCAAAACAGAAGGCTCATTACCGAACCTGCTACTCAGATTCCCTGCGACGCCGTTTCTGGCACGTGACGGACTCACCCCTCGCGAAGGTGACCACGTGGACGGCGTGGCTAACTTACTCGGAGAAGCAGGACGAACCGCAGAACGAAGAGGAGAACCTTGATGTCGGTTGTTCACAACCCCACGTGGCGACGAAACAGGACTATCAAACGTTGACTTTTTAGGCGCTAGAAGCTTCGGAGGAACCGTTGGTTTGTTACTAATTCCGTTTCCATTGTTTCTAGAAGATGGAGGATCCGTTTGGCGCCGTAACCGATTGTTAGCCTCTTGCCAGAACCTCGCTGGAACCACAATTGAACGAGACGCGCGTTGCGTTTGTCCTCCTCCTGAGGAACTACCAGAAGTCACACTTTCGTTATCGGAAAGAACAGGTTCGGGCTCAGGCTCAGGATTCAACTCAGATCCGCCATTTTTGTTGTTCTCTGATCTCAATGTTGACTCATGAGAAGCTCGAACATCTATCATTAAATTCTGCAACGACCTAACAACGTTCCCTTTTTCATTCGATTTTCTCACGGAATCAGTACAATCCAAGCTTCTATTCATGAAATTCGCTTGCTGTTGCAATTTCCCTGGCCATCGGTGTTGATCCAGTGACCTCGAATTCTCTGTCTGATCGGAACTTCCGTTTCCTCCTCTACCGGCCGTTGGCGTCGTCGCCGTTTTTCTCCTCTCCGGCGTGCTCTTCCTTACACTCTGCGGCGGAGGTGTTGGCTTCACCTTACTCACCTGAATCGAGAACGACTCTCCCTGGAACGAAACCGACAAACTCCGTGTCGAAGTGAAAAGCATTTTCTGAGCTGCCGGAGCGTCATTTCCGGCGCCGTTAGGTCGAGGAGTTGCCTGCCGCCGCCGCTCCGCCGACTGAGACCGTTTCAAAGTCGGCGTCGGAACCGGTGTCGACTTTTGTCTGGTTGAACTCACCGTTCTGGACACCAGAGGCGAATTACACCGTCTCGGAGGAGACGAAACGGAAGAAGAGGACGAAGAAGAAGACATGTAACGAGAAGTAACTTCTCGAGCTTTCGGCTTTCGAGGTGGAGCAAGTGCATTGTCCGATTCAGAAGGTAACAATGGAGGCCTCGTTGGTGTTGGTGCTCGCTTTGGGTTTATCGTCGTAGAAATTGCAGCAACCATTGTTATTCCAATTTTCCACTAATTCAAATTGGAAACTCGAAACTCATAGTTGGAATTCACGCTCAGTTTCAGAACCCTAAAAATTTGATTACGAAAAATTAAACTAATCGTAACAGAATTCAGAGTCACGTTGCAG

>Rp-43

TGGGAGGTGGCGGGAGGGGAGGGAGGTCACGCACTCAAAGAAAGCATTTCCGTCAAAGCAGAGAGAATGTATGGAAGCGTTCTAAATCAGATCCAGATCCTTCTTCAAACTCTGAAAACCAAAATCAAAATCAAAATCAAAATACTACGTGGACTCCATTCGCCACTCAGAACACTGCTTTCGATGCCTATTACAAGGAGCAAGGGATAGTTCCCTCTCAAGAGTGGGATGAATTCGTTGCTCTTCTCAGAACTCCATTGCCCGCTTCCTTCAGAATCAATTCGAGTAGCCAGTTTTCTGACGACATTCGTTCTCAACTGGAGAATGATTTCGTGCATTCTCTTCGTTCCGAGGTTGTTGAAGGGGGAGAGACAGAGGCTATTAGGCCATTGCCGTGGTATCCTGGGAACTTTGCTTGGCATTCAAATTTTTCTCGGATGCAGTTGAGGAAGAATCAAACACTTGAGAGGTTCCATGAATTCTTAAAGCTAGAAAATGAAATTGGAAATATCACAAGACAGGAAGCTGTCAGTATGGTTCCTCCTCTCTTCTTGGATGTGCATTCAGATCATTTTGTACTTGATATGTGTGCTGCTCCAGGTTCCAAAACATTCCAGTTGCTTGAGATTATACACCAATCAACTACAGCAGGATCACTACCTGATGGAATGGTTATAGCAAATGATCTTGATGTCCAAAGATGTAATCTTCTCATCCACCAAACAAAAAGAATGTGCACGGCCAACCTAATTGTTACCAATCATGAAGCGCAGCACTTCCCAGGATGCCGTTTAAACAGGAATCATGAAAGGATGGAGTTTGATCTCAATATTGGCCAACTGTTATTTGATCGTGTTCTGTGTGATGTCCCGTGTAGTGGAGATGGTACTCTTCGCAAGGCACCTGATCTCTGGAGGAAATGGAACACAGGGATGGGACATGGGCTTCACAGCCTACAAGTTTTAATTGCTATGCGAGGTTTATCTTTACTTAAAATTGGTGGAAGAATGGTTTATTCAACTTGCTCAATGAATCCTATTGAGAATGAAGCTGTGGTTGCAGAGGTTTTGCGGAGGTGTGGAGGGTCTGTTAAACTTGTTGATGTCTCTAGTGAGCTTCCACAACTTATTCGTCGGCCAGGTCTGAAGAGATGGAAGGTATATGACAAGGGCACTTGGTTAGCCTCTTACAAAGATGTTCCTAAGTTTCGTAGAAGTATAATTCTTCACAGTATGTTTCCTTCTGGCAGAGGCTGTCATCAGGATCTTGTTGACAGTAATTGTAATGTTGACATGGGATATGACATTACTGATGGTGTTAATGGAAATGCTGAAGACGATATTCAAGAAGCAGAGAATCCTGTGATGCCTGAGTGTGCTGAGGAAGTTTCTGATTTCCCTCTAGAGCATTGCATGAGGATAGTGCCTCATGATCAGAACACTGGAGCCTTCTTCATTGCTGTCCTGCAAAAAGTTTCTCCTCTGCCAGCTATTCCCGAAAAACAAAAAATAGAAATTGATGAGAAATATGTAGAACCGGCAAACCAGAGTCTTGAGAATGCACAAGTACTGCAGATTAATTCATCAGAAAGTTCTCATGAAGAAGTCTTAAAAGCAGTTTCAGAGGAAAATGTAAATGACAATGAACCCAAAACAGAAGATTTGGAAGTTGGTCCTGTTACATGTGAAGAACAGAATTCCGAGGAAACCGAGGAGCCCCATAATGTACAAAACATAGCAAAGAAGATTCCAGGTAAAAGAAAGCTACAAATTCAAGGCAAATGGAGAGGTGTTGACCCTGTTGTCTTTTTCAAAGATGAAGCAATTATTAATAGTATAAGGGATTTTTATGGAATCAATGAGCATTTTCCGTTCGATGGTCACCTTGTTACAAGAAACAATGATACAAGTCATGTGAAAAGAATTTATTATGTATCCAAGTCAGTCAAGGATGTTCTTGAGTTGAACTTCTCAGTTGGGCAGCAACTTAAAATAACCTCAATTGGCCTGAAGATGTTTGAAAGACAAACAGCACGACAAGGTACCTCTGCACCATGTACTTTCCGGATATCGTCTGAAGGATTGCCCCTTATTCTCCCATACATAACCAAACAAATTCTACGGGCATCTCCCGTAGTCTTCAAGCATCTTTTGCAGAACAAAGATGTAAAATTTGAAGATTTTGATGATGCCGAGTTTGGTGAAAAGGCAGCAAACCTACTGCCAGGCTGTTGTGTGGTGATTATGGATAAAGGAAACACAGTTGCTTCAGAGCCCCTCAAAGAGGATGAGTCAACAATAGCCATTGGATGCTGGAAGGGTCGGGCGAGATTGACAGTGATGGTTACTGCAATGGACTGCCAGGAACTGCTTGAAAGGCTTTTAATACGTCTAGACACAGAAAAGGGCTCCTCTGGGCATGTGGACAAATCCTCTAATAATAATGTAGAAGGTGAAGGACAGCCTGTACATGAGTTGAACGGTAAAAATGATGATGATGTGAAAGCTTCTGTGTGTTAATA

>Rp-44

AAATACAGTGTTTCCAGAACCTCGCCTCACACGCACTATCTGGGAGAATCGAGAGCAGTAGTTGAAGATCATATTCATAGTTAGCGCAGGATTCTCGATCATCAAGCATCAATCAATCAATCAATCACGAGTTTGATGAATCCAAAAACTGACAAGCTGGTTAGAAGGACAACCATGGTGGCAACCGTTACTGCTTCCTATTTTCTCTTAACCGCTGACTATGGCCCTCAACCCAATGTTCTCGACCCTATCAAGAAGAGAATACTTTCAGCAGAGAGCACTGTGAAAGAGTATATTTTTGGATCAAAAACAGAATCTCAAGAGAAGCACATAGAGAAAAAATTGGACAGCGACAAGGATCATCCATAAATTTATGGTCATCTTGTACAGGGATGTTATGCTGAGTCAAGGTTTTCTTTTTAGCCGTGTGAACTCGTGAATGTCTCTAGTCTAATGGATTGGTTAACTGACTAGAGGCTGAGAATGTATAAATGGTAGAAGCTATCTTGATGGACTTATCTGCACTATCAATGCCATCAATTAGACAATAACATCATGATCATGTTTTCTGCTGCTTCCCTATTCCCCCACCTCCCCCCCTTCTCTCTTAAACTATGTATCTGGC

>Rp-45

CTAAAATTCCAGGGTAAGATTATTTATGATATAATTTCAACATTTGCACTGAAAAACGGAGAAAATGTATACATACAGAACAGTGAAACACAAGGGCTGCAGCTACATACATACGGAAACTTTTCATATTGCAAACTATGTTTCAATGGAAGTTCATATAATTACAGCATCGTCCTCAAATCACCATCCATATTTCGCTTGTGTTTCAGCTTTGGTAAGCATGCCCTTTGAACGCCGCTCGGCGAGTTTCATTTCATTTTGTTTCATATCAAAGTACAACGAACCAATTTGATGCTTCCTCTTGTGCAGCTTTGTAGGCTTCCCCTTTGTCGAAACAGGCTGGTAAGAGGGTCCAAAAGCTATCCCAGTTAGCTTAGCCTGATCTTCTCTTGGCCTGTTCTTAATCAACTCATCTTGCTTCACCTCAATCACTTCCATGGGAACCTCATTCCTACCTCTCTTCCCAGGAATTTTCACCACACTTTCACTAATCCCGGAAGCCTCTTGCACCGACCCATCAACCCAGTTATTCCCATACTGTCCATAATCCCCATAGGCACCACCATAGCCACTATCATTATTTCCATAACCAGAAGTACCAACACCAGCACCAGCCTCTTGCTCAATACCCCAACTGTAATTGCCATAACCATCATACTGCTCAGTGGCATATTGGTAATTCTCATAATCCCCTGCATTATTTTGATCAACTGGGACACTATCCTCTTCGGCGGAACCACCAGAATTTGACGCCGGTGCAGGTGTATCGGTTTCAATGATGGATCTCCGGCCAGAACCGGAACTCGATTTAACACCAAGAGTGGCGGAGTTCTTTGGTGCTGGAATGCTGGACAAGAATGATTTCACCGATGGGGTTTGAATGGGAGATTCCAACTTTCTTCTTCTGTTTCGTTCTTCTTCTTCTTCGTCGTCGTCATCATCTTCGAGTTCGGTGGGCTTCGGCAAAGGGATGATTGGGGGTTTGAATAGCACAACCCTTTTGGGTTGTTTATGTTCTTCGATTTGGGATTTAGGGTTCGGGAGGGAAGCGCTTAAACTCGAATTCGACAAAGTCCCAGAACGTGTATCTTTCGCAGTTGCAGGGGAAGGTTGTTTGGGTTGAGGAAGAGATTGAAAGAGCGAAGAAGAAGATTTGGGTTGAGGAAGAGAGGAGAAGAGGAAGGAAGAAGACGACGTCGTTTTGCATGGGATTGTTGGTTTCTGTGGTTGTGGTTCTTCTTCTTCTTCTTCTTCTGAAGAAGCGTAGTTTGCTAGTAGAGAATCCATGATCACCAACACGATTCGATTTTTGCACTCTTCTCTGCTCGATGAGATGACGATGACCCACCCAAACCCTAGCTCCCTT
