# Supplementary material for: Development and Evaluation of a Novel Set of EST-SSR Markers Based on Transcriptome Sequences of Black Locust (Robinia pseudoacacia L.)
Source: Genes (Basel). 2017 Jul 7;8(7):177. doi: 10.3390/genes8070177 (PMC5541310; doi:10.3390/genes8070177)
Supplement: Supplementary file 1 [file genes-08-00177-s001.zip › Table S1.docx]

**Table S1. List of the experiment individuals of *Robinia pseudoacacia* L.**

| NO. | Varieties | Origin |
| --- | --- | --- |
| 1 | 12 | Henan |
| 2 | 39 | Henan |
| 3 | 82 | Henan |
| 4 | 139 | Henan |
| 5 | 174 | Henan |
| 6 | 195 | Henan |
| 7 | 221 | Henan |
| 8 | 261 | Henan |
| 9 | QG46 | Shanxi |
| 10 | QG111 | Shanxi |
| 11 | WL15 | Shanxi |
| 12 | WJ20 | Shanxi |
| 13 | WA8 | Shanxi |
| 14 | WT8 | Shanxi |
| 15 | B3 | Shanxi |
| 16 | B122 | Shanxi |
| 17 | Lu-068 | Shandong |
| 18 | Lei-02 | Shandong |
| 19 | Lie-09 | Shandong |
| 20 | Xing-08 | Shandong |
| 21 | Xing-24 | Shandong |
| 22 | K2 | Shandong |
| 23 | Jiangan-1 | Shandong |
| 24 | 8062 | Shandong |
| 25 | 227 | Hebei |
| 26 | 152 | Hebei |
| 27 | 96 | Hebei |
| 28 | 32 | Hebei |
| 29 | 8 | Hebei |
| 30 | 11 | Hebei |
| 31 | 34 | Hebei |
| 32 | 46 | Hebei |
